# Barrier island geomorphic complexity drives storm and post-storm response

**DOI:** 10.1038/s41467-026-77143-6

**Published:** 2026-09-24

**Authors:** Jennifer L. Miselis, Noreen A. Buster, Daniel J. Ciarletta, Julie C. Bernier, Emily A. Wei, Rose V. Palermo

**Affiliations:** 1 U.S. Geological Survey, St. Petersburg Coastal and Marine Science Center, 600 4th St. S, St, Petersburg, FL USA; 2 Louisiana State University, Department of Geology and Geophysics, E235 Howe Russell Kniffen, Baton Rouge, LA USA

**Keywords:** Ocean sciences, Natural hazards

## Abstract

Barrier islands are at the forefront of climate-driven changes, with their resiliency threatened by rapid fluctuations in the physical drivers that shape them. Quantifying magnitudes and directions of storm-driven sediment fluxes and rates and modes of sediment recovery is essential for predicting barrier island trajectories. Yet scarce shoreface observations have limited such efforts. Here, shoreface-inclusive measurements of barrier sediment fluxes over five years following an extreme storm reveal distinct sediment redistribution patterns that vary with island geomorphology. Overwash fluxes were low compared with seaward fluxes, inlet-related landward transport, and previously modeled estimates. Without these observations, models may overestimate island elevation-building capacity that is key to sea-level adaptability. Post-storm recovery was incomplete despite human intervention, altering barrier sediment distribution from pre-storm conditions. This may increase vulnerability to future storms and rising water levels and suggests management approaches tailored to island geometry may better compete with climate change effects.

## Abstract (web summary)

The study shows that barrier-island geomorphology shapes sediment redistribution during and after storms, and that incomplete post-storm recovery and overestimated elevation-building capacity may affect predictions of island resilience to climate change.

## Introduction

**Fig. 1:**
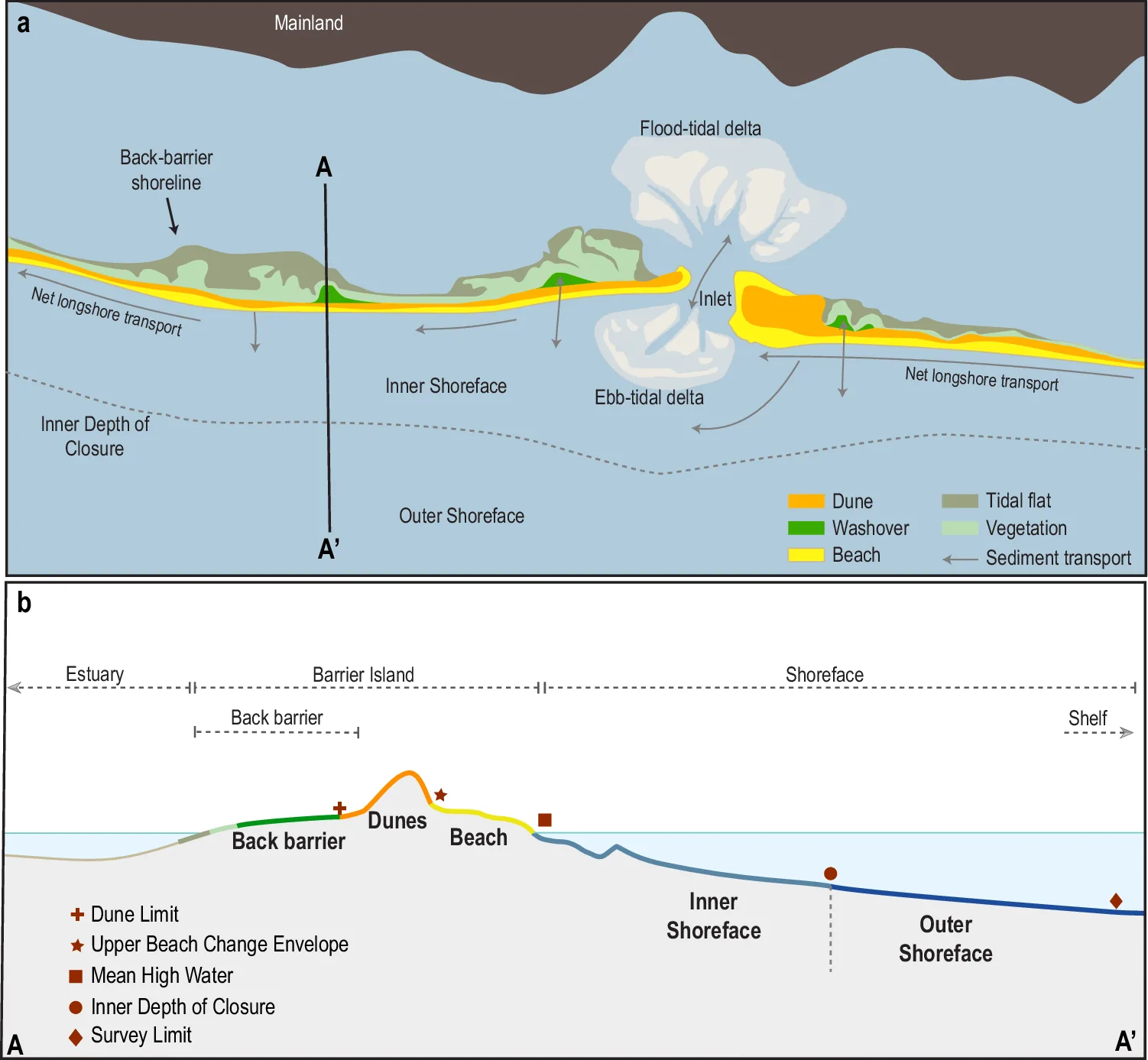
A barrier island system, its environments, and sediment transport pathways. Modified from Miselis et al., 2021^25^. Plan view (**a**) and cross-section (**b**) of a barrier island system, its primary sediment transport pathways (gray arrows, panel **a**), and the general location of the boundaries used to separate barrier island environments (red markers, panel **b**). In this study, the outer shoreface boundary is limited by the extent of the pre-storm survey, which corresponds to an average water depth of − 8.6 m.

Barrier islands and spits, collectively known as barriers, are globally distributed coastal landforms fronting 10% of open-ocean coastlines^1^. Their form and dynamics reflect a balance between wave and tidal energy, sediment supply, and rate of sea level rise (SLR)^2^. In natural barrier systems, storms drive exchanges of sediment to and from the shoreface, beach, dunes, and back-barrier to maintain island heights, widths, and cross-shore positions in response to changes in sea level^3,4^(Fig. 1). However, the drivers of barrier formation and resilience over geologic timescales are changing in two critical ways.

First, the combined impact of more extreme tropical cyclones^5^, possible increases in global extreme wave events^6^, and an increase in mean sea level since the late 20^th^ century^7^ is threatening to significantly change the global hydrodynamic climate, likely increasing sandy beach erosion^8,9^, including on barrier islands, and possibly changing the likelihood of whether overwash or breaching dominates storm response^10,11^. Also, recent work suggests geomorphologic complexity will promote alongshore-variable barrier responses to changes in SLR, storm climatology, and sediment supply, delaying the landward translation of barriers over decadal or longer timescales^12,13^. To predict the benefit of this hysteresis (e.g., how long barriers can continue to effectively provide natural and human services), a better understanding of the feedbacks between barrier sediment redistribution and geomorphology, particularly during and after storms, is required^14^.

Second, humans are actively altering sediment transport pathways on barrier islands^15–17^. Over the last several decades, populations and development in coastal areas and on barriers have increased dramatically^18,19^ and future projections indicate more of the same^18,20^. Fifty-nine percent of the world’s barriers have some type of development, two-thirds of which are in the United States^21^, and this has increased human exposure to coastal and climate change hazards^18,20^. To protect lives and livelihoods, coastal communities alter barrier islands by strategically adding and removing sediments (e.g., nourishment and dredging). This changes barrier morphology in ways that alter future vulnerability to storms and SLR^16,22–24^. But the scale and impacts of these interventions have not been adequately quantified^17,25^ and may manifest as near-term stability in geomorphic state at the expense of dynamic behavior that builds long-term resilience^26^. With global sand supplies waning in response to rapid population growth and associated development^27^, it is unclear how long holding the line will be economically viable and desirable, particularly if the short- and long-term benefits of doing so are uncertain. To predict barrier behavior over short and long timescales, human alterations to barriers and their sediment supplies must be accounted for and quantified^15^.

The intersection of increasing human risk and uncertainty in future barrier behavior has motivated many modeling investigations seeking to predict barrier behavior due to SLR and natural and human-mediated variations in sediment flux, or the exchange of sediment along barriers and between barrier island environments over time^4,12,15,23,28–32^. These models explore when and how barrier islands will reach morphologic tipping points, at which time they will no longer provide the economic and ecosystem services of the past and present. It is unclear if the range of values driving these models is realistic because few observational studies have quantified all relevant sediment fluxes, let alone over both storm and non-storm periods^33^. For example, overwash flux parameterization often relies on localized field measurements that are difficult to extrapolate regionally^34^. Recent laboratory and desktop advances in quantifying overwash fluxes over large spatial domains have yet to be applied broadly and have often assumed uniform barrier morphology^34^. Fluxes to and from the shoreface, a primary driver of modeled barrier behavior^4^, are even less constrained. Recent work demonstrates that fluxes from the barrier to the shoreface are common^35–37^ and may be capable of offsetting decades of SLR-mediated shoreline retreat^38^; how the magnitudes of those offshore fluxes vary spatially, how much subsequently moves back onshore, and the impact of these processes on future barrier behavior are unknown^39^.

Despite the clear need for better sediment flux estimates along barriers and between their environments, quantifying the magnitudes of these fluxes in the field is challenging. Barrier responses to storms and SLR are not uniform^12,31,32^ due to alongshore variations in water levels related to tides, surge, and wave runup^40^; shoreface, dune and beach morphology^40,41^; sediment availability^42–46^; vegetation^47^; presence of coastal infrastructure^15,22,23,48,49^; and human behavior^16,50^. Therefore, measurements made over small spatial scales (e.g., 1-2 km) may not represent conditions in geomorphically distinct parts of the barrier system. Seaward fluxes are difficult to quantify at any spatial scale, since rapidly deployable techniques are often limited to the terrestrial portion of barrier islands^36,37^ or are not yet capable of covering large spatial scales that would span different barrier geomorphologic configurations^51^. Recent work from headland-beach systems highlights the importance of quantifying storm-related shoreface fluxes for understanding coastal response to SLR^38^, though it is unclear whether similar rates of post-storm recovery would be observed on barriers, where extreme storm impacts can persist for several years^52,53^.

**Fig. 2:**
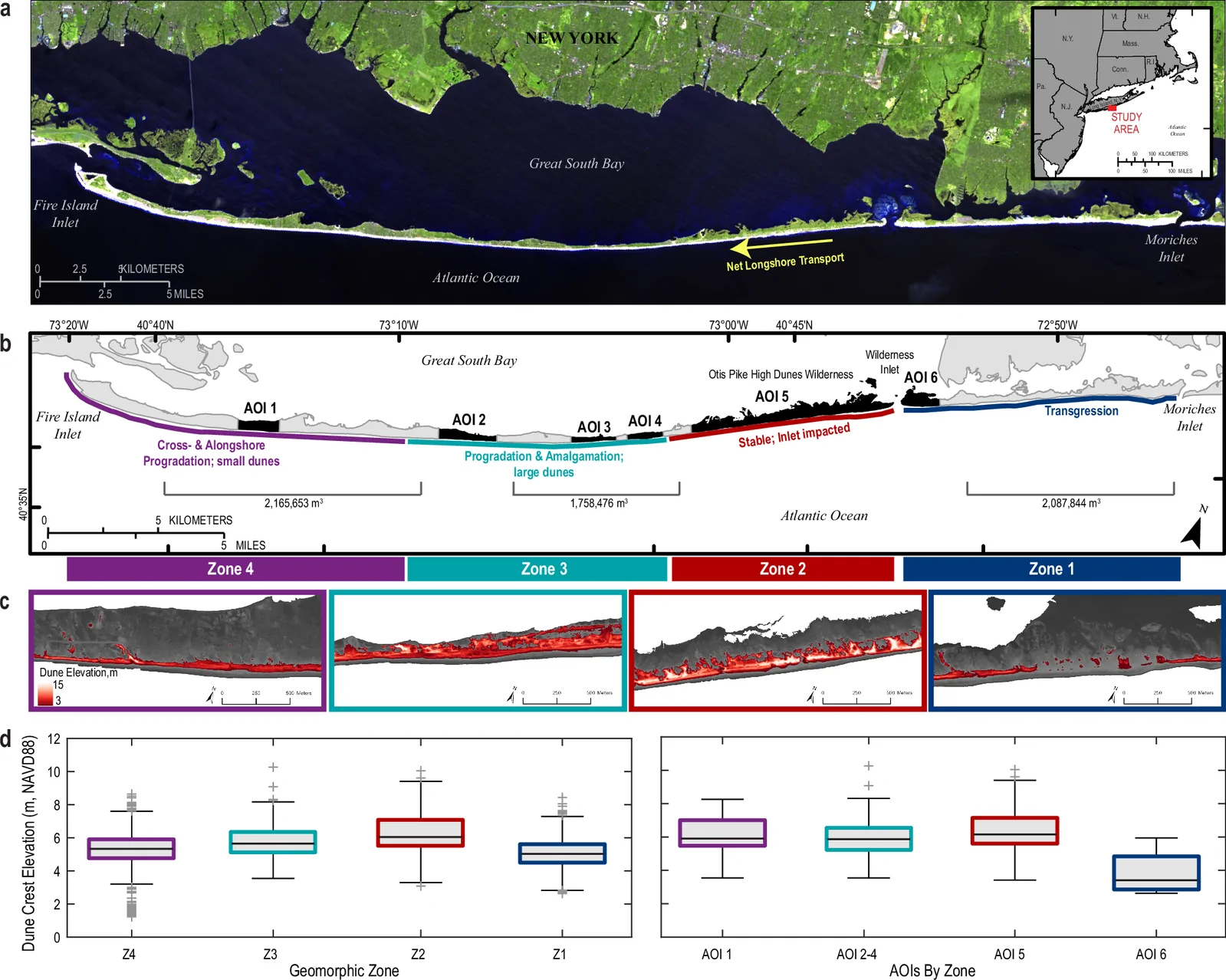
The study area and its geomorphology. **a** The study area location in New York, United States.(Landsat 8 image acquired 04-May-2017; false natural color image displays bands 7,6,4; https://www.usgs.gov/landsat-missions/landsat-data-access). **b** The geomorphic zones of Fire Island, based on the work of Ciarletta et al., 2024^26^, the locations of areas of interest (AOIs; black polygons), and volumes of placed sediment added during the post-storm period^59^. Basemap modified from New York State Office of Information Technology Services GIS Program Office (1:24,000 scale; https://data.gis.ny.gov). **c** Characteristic pre-storm dune morphology in each of the geomorphic zones with elevations in NAVD88^76^. **d** Box plots of pre-storm dune elevations in each geomorphic zone. The central mark represents the median value, whereas the top and bottom of the boxes represent the 75^th^ and 25^th^ percentiles, respectively. The gray crosses represent statistical outliers, and the vertical bars extending from the boxes extend to the most extreme values that are not outliers. **e** Box plots of dune elevation in each AOI, grouped by geomorphic zone.

Here, we quantify storm-period and post-storm period sediment volume changes and calculate sediment fluxes using observations from Fire Island, New York (NY), a wave-dominated barrier system on the Atlantic coast of the United States (Fig. 2a). The dataset encompasses the island’s alongshore-variable geomorphic zones^26^ (Fig. 2b). This allows us to quantify how barrier geomorphology influences storm-related sediment flux magnitudes and directions that may ultimately drive longer term behavior^12,29^ and to extend the observations made here to other barriers with similar morphologies. Importantly, the observations span all sandy cross-shore barrier island environments, including back-barrier, dune, beach, and shoreface, the latter being critical for helping to explore the shoreface knowledge gap^39,54,55^ and for quantifying the shoreface contribution to barrier behavior relative to other barrier sediment reservoirs, such as dunes^56,57^. The data capture the impacts of an extreme storm, Hurricane Sandy, and a prolonged post-storm period of almost five years, intentionally extended to capture realistic recovery timelines^52,53^. Although the analysis does not include developed parts of the barrier, the post-storm period includes natural and human alterations to barrier sediment supply, furthering the portability of the results.

## Results

Hurricane Sandy made landfall on 29 October 2012 causing extreme waves and water levels along the coasts of New Jersey, New York, and Connecticut on the United States east coast (Supplementary Figs. S1, S2; hydrodynamic details in Methods)^58^. The affected area included Fire Island, NY, a 51-km, sparsely populated barrier island (Fig. 2). The island displays a geomorphic gradient, following the east-west direction of net longshore sediment transport and is divisible into four zones representing different geomorphic states: Zone 1 is transgressional; Zone 2 prograded historically ( < 150 years ago); Zone 3 prograded over geologic timescales ( > 600 years ago); and Zone 4 is elongational (Fig. 2; detailed descriptions in Methods)^26^. Hurricane Sandy breached Fire Island in several places although only Wilderness Inlet, at the downdrift boundary of Zone 1 and updrift boundary of Zone 2, was left open to evolve naturally (Fig. 2). To limit the influence of infrastructure and direct human modifications^22,23^ on measured volume changes, our analysis focused on six predominantly natural areas greater than 1 km in alongshore length, resulting in at least one area of interest (AOI) in each geomorphic zone (Fig. 2b). Since the relationship between dune elevation and total water levels is a good predictor of coastal storm impacts^40^, similarities between zone- (Fig. 2d) and AOI-averaged (Fig. 2e) dune crest elevations suggests that changes observed in the AOIs are representative of the zonal geomorphology, allowing exploration of how alongshore variations in island morphology alter storm and post-storm sediment fluxes.

### Storm-period volume changes

**Fig. 3:**
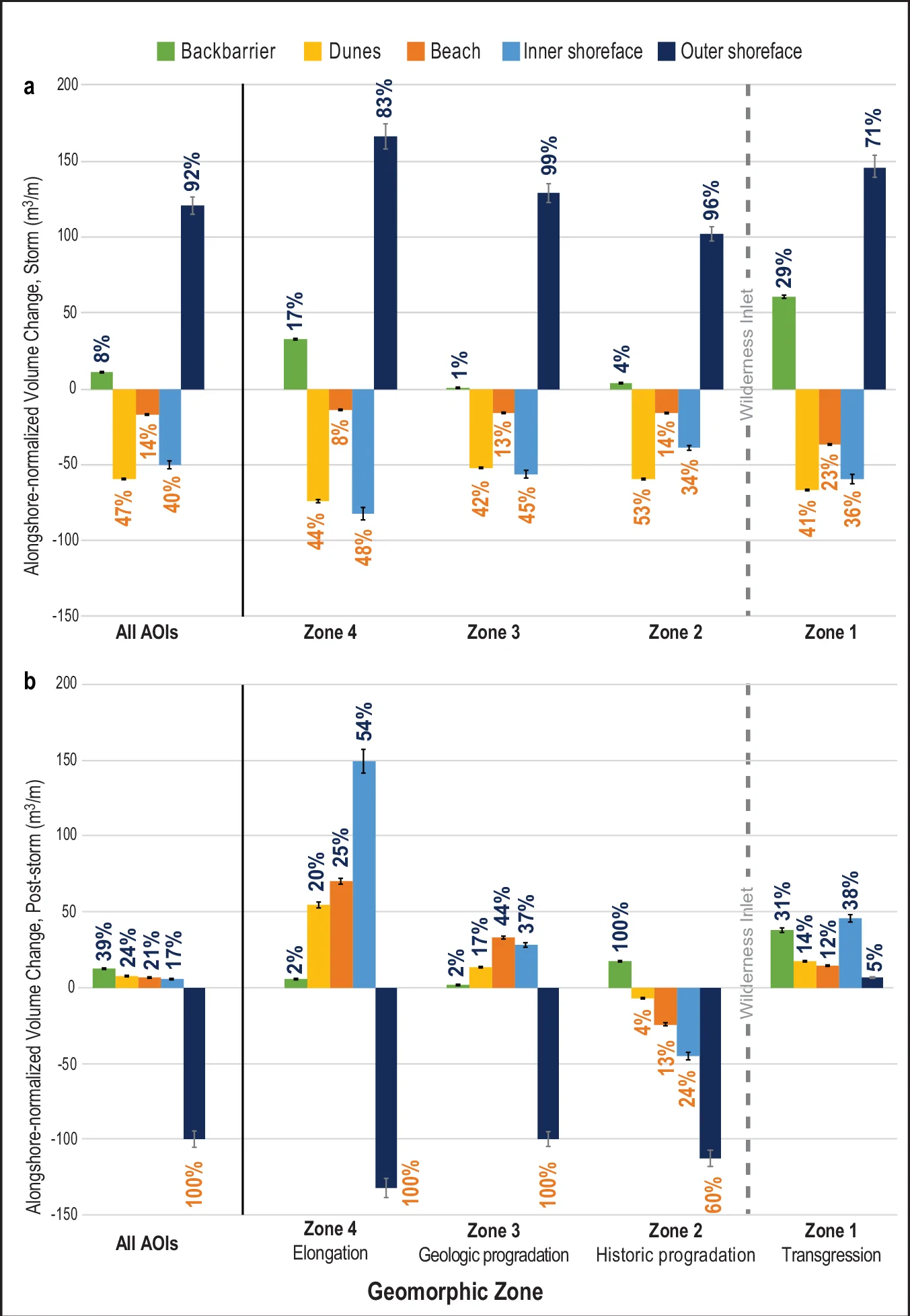
Storm and post-storm period volume changes. Alongshore-normalized changes in sediment volume in outer shoreface, inner shoreface, beach, dune, and back-barrier environments during the storm (**a**) and post-storm (**b**) periods. Dark blue numbers at the ends of the bars indicate how volumes were distributed among environments in which net accretion was measured, whereas orange numbers indicate how volumes were distributed among environments in which net erosion was measured. Note that some totals may not add to 100% due to rounding to avoid decimal percentages.

**Fig. 4:**
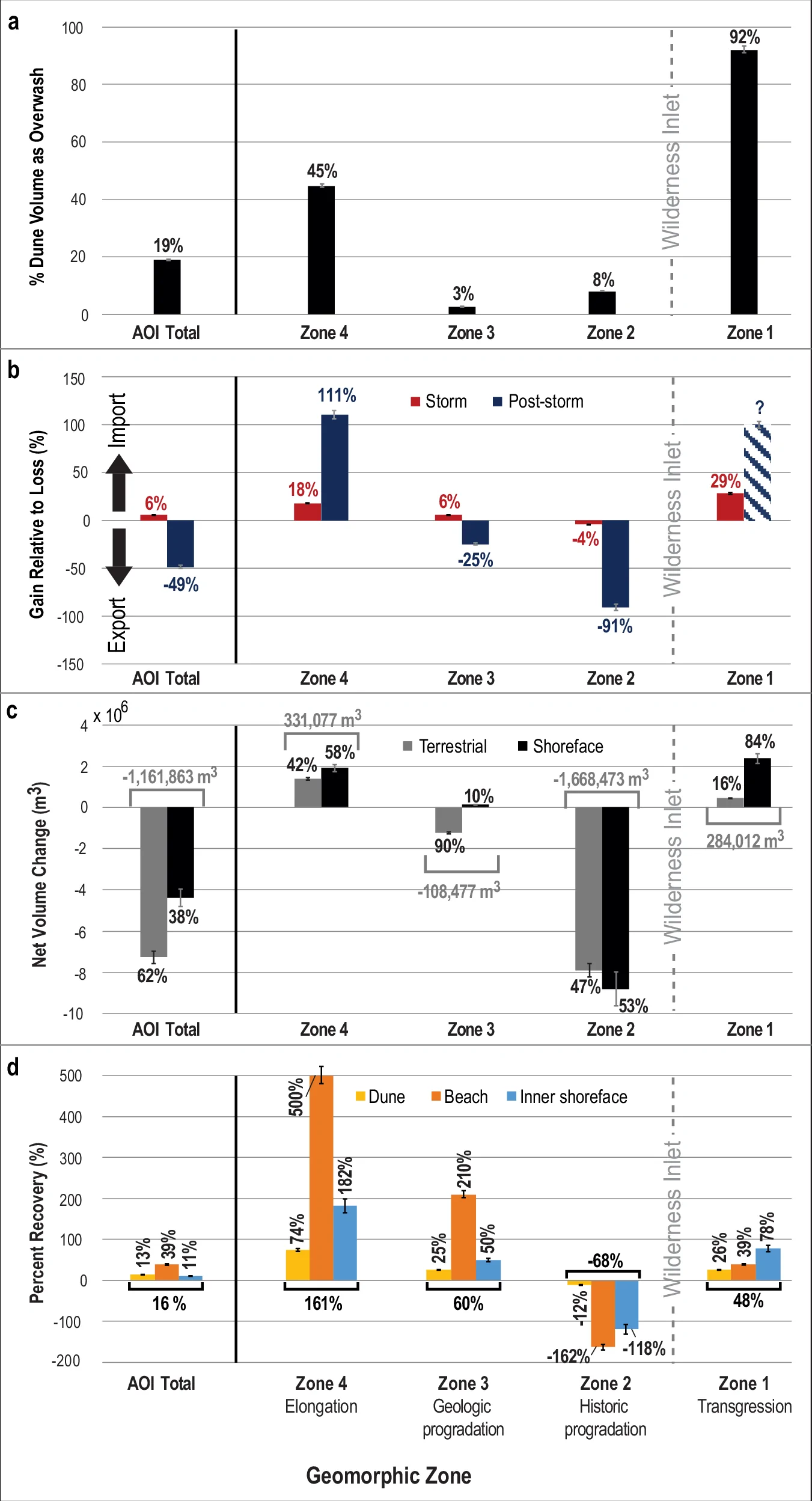
Differences in storm and post-storm responses between geomorphic zones. **a** Percentage of dune volume loss that could have contributed to washover deposition in the back-barrier environment. **b** Percent volume gain relative to volume loss for the storm and post-storm periods. Positive values indicate import (zone is a net sediment sink) and negative values indicate export (zone is a net sediment source). Percentages close to zero indicate a better balance between volume losses and gains. Zone 1 experienced no loss in the post-storm period and therefore a value could not be calculated. **c** Comparison of terrestrial and shoreface net volume changes during the study period. Numbers adjacent to brackets represent the net volume change over the period of observation, whereas percentages indicate the distribution of the net change between shoreface and terrestrial environments. **d** Percent recovery in inner shoreface, beach, and dune barrier island environments. Percentages at the end of the bars represent the percent recovery for that environment, whereas the percentages adjacent to brackets indicate the percent overall recovery. Negative numbers indicate net losses from these environments for the study period.

During the storm period, all AOIs experienced erosion in dune, beach, and inner shoreface environments, whereas deposition occurred in back-barrier and outer shoreface environments (Fig. 3a). Of the total volume deposited during the storm period, 92% occurred on the outer shoreface and 8% occurred in the back-barrier (e.g., washover; Fig. 3a). Dunes contributed significant volumes of sediment to the outer shoreface since only 19% of eroded dune sediment could be accounted for by washover deposition (Fig. 4a). Accumulation in the back-barrier and on the outer shoreface exceeded losses from the dunes, beach, and inner shoreface by about 6% (Fig. 4b), suggesting some sediment was contributed from outside AOI boundaries.

Relative magnitudes of storm-related volume change varied with alongshore differences in island geomorphology (Fig. 3a). For example, the extent to which dune environment losses translated to washover volume varied (Fig. 4a). In transgressive Zone 1, washover volume accounted for 92% of dune volume losses whereas much lower percentages were observed in Zones 2-4 (Fig. 4a). Zone differences in the proportion of storm-period washover deposition ranged from 29% to 1% of total deposited volume (Fig. 3a). Differences in the balance of storm-period erosion and deposition along the island also reflected barrier geomorphologic variability (Fig. 4b). Historically progradational Zone 2 experienced the closest balance between storm-period erosion and deposition volumes, with minor net export (Fig. 4b). Every other zone experienced net import, or sediment transport from outside the AOIs, the highest of which occurred in Zone 1 (Fig. 4b). These results demonstrate that alongshore variations in barrier geomorphology influence how sediment reservoirs in barrier environments behave and interact during storms.

### Post-storm period volume changes

Summed across all AOIs, post-storm period volume changes were characterized by accumulation in all barrier island environments except the outer shoreface (Fig. 3b). The magnitude of accumulation volume increased from the inner shoreface to the back-barrier (Fig. 3b). Total post-storm deposition represented only 33% of the volume eroded from the outer shoreface. Of the total volume of sediment deposited in the outer shoreface across all AOIs during the storm, 18% remained at the end of the period of observation. This suggests that almost half of the outer shoreface storm deposit was transported beyond the analysis boundaries and not back to terrestrial and inner shoreface environments within the AOIs.

Post-storm volume changes did not manifest in the same way across all geomorphic zones (Fig. 3b), with behavior reflecting varying influences of natural post-storm adjustments and estimated anthropogenic sediment additions of up to 6,000,000 m^3^ (Fig. 2b)^59^. For example, the natural equilibration of Wilderness Inlet influenced post-storm responses in Zones 1 and 2 in very different ways. Updrift of the inlet, deposition was measured in all environments, whereas downdrift of the inlet, erosion was measured in all environments except the back-barrier (Fig. 3b). Since no post-storm losses were measured in Zone 1, all sediment had to be sourced from either updrift nourishment or otherwise beyond the AOI boundaries. Furthermore, ebb tidal delta growth during inlet equilibration likely helped enhance accumulation in Zone 1, trapping sediment that might have otherwise been transported alongshore. Inlet tidal delta growth reduced alongshore sediment supply to Zone 2, resulting in post-storm volume losses 11 times greater than measured accumulation (Fig. 3b). These losses coincide with the landward translation of the barrier in the section of Zone 2 adjacent to the inlet. Zone 2 was the only zone in which the storm-period shoreface deposit was completely eroded; in fact, post-storm erosion exceeded storm-period deposition by about 10%. If we attribute post-storm accumulation in the AOI to erosion of outer shoreface sediment, it would have represented only ~ 15% of the eroded volume. This means 94% of what was eroded from the outer shoreface during the post-storm period is unaccounted for in Zone 2, likely being transported landward (e.g., into the inlet) or alongshore outside of the AOI boundaries.

Outside of inlet influence, post-storm volume changes in geologically progradational Zone 3 and elongational Zone 4 followed the trend for all AOIs, with deposition measured in all environments except the outer shoreface. Assuming again that outer shoreface erosion contributed to post-storm accumulation in the AOIs, 75% and 211% of eroded shoreface volume remained in the AOIs for Zones 3 and 4, respectively. Accounting for the remaining outer shoreface storm deposit, only 1.4% of the eroded outer shoreface volume is unaccounted for in Zone 3, whereas about 53% of the post-storm accumulation in Zone 4 was sourced outside of the AOI boundaries. Importantly, the magnitudes and proportions of accumulation varied, with the greatest accumulation in the beach environment in Zone 3 and in the inner shoreface in Zone 4 (Fig. 3b), where it represented more than half of all post-storm period volume accumulation (Fig. 3b). This resulted from direct nourishment^59^ and its position near the littoral end point of the system where it was more likely to benefit from sediments lost from other zones. Lastly, the post-storm distribution of accumulation across terrestrial environments varied spatially. In Zone 1, the back-barrier environment experienced the highest proportions of terrestrial accumulation, whereas in Zones 3 and 4, it was the beach (Fig. 3b). This suggests that in addition to anthropogenic influences, barrier geomorphologic history and the resulting shape and extent of barrier environment sediment reservoirs, are a control on the style of post-storm recovery and the environments in which post-storm sediments accumulate.

In contrast with the storm period, dramatic imbalances between erosion and deposition in each zone characterized the post-storm period (Fig. 4b). Net sediment export was observed in Zones 2 and 3 (Fig. 4b). This is expected in Zone 2 due to inlet-mediated decreases in updrift sediment supply that caused extensive post-storm volume losses (Fig. 3b). Despite being downdrift, volume losses in Zone 3 were not offset by Zone 2 losses (Fig. 4b). They may have contributed to an increase in Zone 3 beach volume, however, since it was the highest proportional post-storm volume increase in the beach environment (44%; Fig. 3b). Conversely, extreme net sediment import was measured in Zone 1, where all environments recorded accumulation, and in Zone 4, where accumulation volumes were more than two times eroded volumes (Fig. 3b). These trends were likely driven by varying influences of direct (Zone 4) and indirect (Zone 1) nourishment, inlet equilibration (Zone 1), and naturally cascading longshore sediment transport, in which downdrift zones benefit from updrift losses (Zone 4).

### Net volume changes & post-storm recovery

**Fig. 5:**
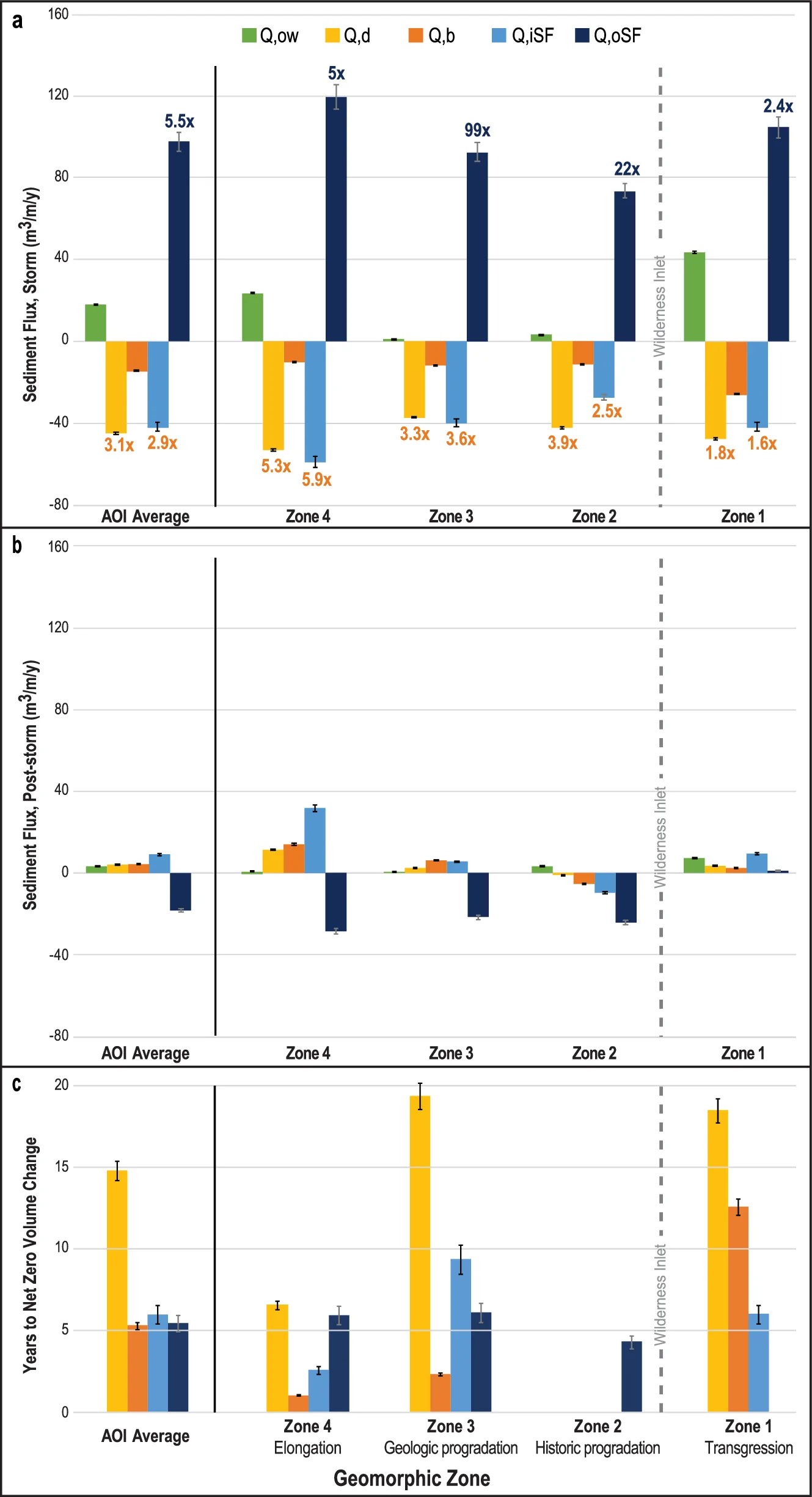
Differences in storm and post-storm period sediment fluxes and recovery rates between geomorphic zones. Estimated sediment fluxes into (positive) or out of (negative) each barrier island environment in each zone for the (**a**) storm and (**b**) post-storm periods. Text in blue indicates how many times greater the magnitude of the flux to the outer shoreface (Q,_oSF_) is compared to the magnitude of the flux to the back-barrier (Q,_ow_). Text in orange indicates how many times greater the magnitude of the inner shoreface flux (Q,_iSF_) or dune flux (Q,_d_) is compared to beach fluxes (Q,_b_). **c** Length of time required to achieve net zero volume change in barrier island environments based on post-storm sediment fluxes. The back barrier environment is not included since it is expected that sediment transported there is unlikely to move elsewhere over the time scales considered here.

The combined impact of storm and post-storm periods on sediment distribution along and across the barrier is explored by examining net volume changes (Fig. 4c). Further, to assess how barrier island configuration related to post-storm behavior, we quantified recovery in three ways: (1) overall recovery, or the extent to which the AOIs recover sediment lost during the storm period (Figs. 4d), (2) recovery distribution, or how overall recovery is distributed amongst the various barrier island environments (Figs. 4d), and (3) rates of recovery, or the rate at which net zero volume change would be attained based on measured post-storm fluxes (Fig. 5c). Despite human interventions, there was a net loss of ~ 1,200,000 m^3^ across all AOIs, 62% of that from the terrestrial component of the system and 38% from the shoreface (Fig. 4c). Post-storm accumulation and placed sediment were not enough to offset storm-period erosion within the AOIs, with overall recovery of only 16% across all AOIs (Fig. 4d). Net recovery distribution of 13% of dune volume, 39% of beach volume, and 11% of inner shoreface volume was measured (Fig. 4d). These results underscore the lingering impact extreme storms have on barrier islands and provide insight into the scale of sediment resources required to offset them.

As with storm- and post-storm responses, net volume changes and recovery patterns in barrier island environments varied with alongshore changes in geomorphology. Transgressive Zone 1 and elongational Zone 4 experienced net sediment accumulation in both terrestrial and shoreface environments but in different proportions (Fig. 4c). In Zone 4, net volume changes were almost balanced between the shoreface and the island, whereas in Zone 1, net volume increases on the shoreface dominated, likely due to the growth of the adjacent ebb tidal delta and associated trapping of updrift sediment (Fig. 4c). When only considering where erosion was measured in the storm period in Zone 4, 161% of the sediment lost was recovered, owing to wildly disproportionate recovery distributions in beach and inner shoreface environments and the highest percentage of dune recovery (74%), all likely driven by direct nourishment (Fig. 4d). In contrast, just under half of the overall eroded sediment volume was recovered in Zone 1, owing to accumulation on the inner shoreface rather than significant beach or dune recovery (Fig. 4d). These contrasts suggest that barrier morphologic state and sediment placement influence the efficiency of post-storm cross-shore sediment fluxes when sediment supplies are positive.

Net volume losses observed in Zones 2 and 3 had very different magnitudes (Fig. 4c). Historically progradational Zone 2 experienced a net volume loss of ~ 1,700,000 m^3^ which was distributed almost equally between the island and the shoreface (Fig. 4c). Post-storm volume losses in Zone 2 were 1.7 times greater than storm-period volume losses (Fig. 3b). This drastic loss of sediment, driven by the equilibration of Wilderness Inlet, was characterized by extreme net volume losses in beach and inner shoreface environments and continued erosion in the dune environment (Fig. 4d), which combined to alter the downdrift barrier morphologic state. Modest net overall volume loss in geologically progradational Zone 3 (Fig. 4c) contrasted with beach recovery that exceeded 200% of storm-eroded volume, likely owing to large increases in updrift sediment supply from Zone 2 (Fig. 4b, c); however, recovery was incomplete in other environments (Fig. 4d). Taken together, these data demonstrate how both pre-storm morphology and natural and anthropogenic perturbations complicate recovery and alter the distribution of sediments along and across barrier islands.

### Sediment fluxes

Though exploring volume changes and their relative balance are helpful for understanding how storm and post-storm periods alter sediment distribution in barrier systems, sediment fluxes tell us how quickly sediments are lost and replenished (Fig. 5). On average, storm fluxes to the outer shoreface (Q,_oSF_) were 5.5 times the magnitude of fluxes to the back barrier (Q,_ow_, Fig. 5a). However, the difference in magnitude between seaward fluxes and overwash fluxes varied alongshore, with Q,_oSF_ being as little as 2.4 times Q,_ow_ for transgressive Zone 1, to 99 times for the geologically progradational Zone 3 (Fig. 5a). On average, storm-period fluxes from the dunes and inner shoreface were about three times the magnitude of fluxes from the beach (Fig. 5a); however, these proportions changed with barrier island morphology. For example, in Zone 1, the beach is a more substantial reservoir, with dune and inner shoreface fluxes exceeding beach fluxes by factors of 1.8 and 1.6, respectively (Fig. 5a). In contrast, sediment fluxes from the beach environment were less substantial in elongational Zone 4, where dune and inner shoreface fluxes exceeded beach fluxes by factors of 5.3 and 5.9, respectively (Fig. 5a). These data highlight how the relative importance of barrier island sediment reservoirs differs with barrier island morphologic state.

Post-storm-period sediment fluxes were, on average, 10 times less than storm-period fluxes (Fig. 5b) helping to explain the lack of overall recovery observed in many barrier island environments and alongshore variations therein (Fig. 4d). The somewhat landward decrease in post-storm sediment fluxes from the inner shoreface to the back-barrier in Zones 3 and 4 was not observed in Zone 1, where the second highest flux was to the back-barrier instead of the beach (Fig. 5b). These observations suggest that for some barrier morphologies, sediments move landward successionally from one environment to the next whereas in others, this succession happens faster or not at all.

Post-storm fluxes can be used to estimate the time it would take to reverse storm-period changes and restore pre-storm island configurations (Fig. 5c) or rates of recovery. Averaged across AOIs and consistent with slower aeolian processes, dune volume recovery would take the longest at 14.8 y (Fig. 5c). Consistent with hydrodynamic process drivers, average inner and outer shoreface recovery times are shorter, at 5.4 and 5.3 y, respectively. Notable deviations from these averages occurred within certain zones. For example, recovery times in Zone 4, which was directly nourished and is near the littoral end point of the island, were well below average in most environments. Interestingly, the longest time to full dune recovery was measured in Zone 3 (19.3 y) despite updrift nourishment and enormous volumes of sediment lost from inlet-impacted Zone 2 (Figs. 3b, c, 5c). Beach volume recovery times ranged from 1 y near the littoral end point of the system to 12.5 y in Zone 1. Finally, with the exception of Zone 1, comparisons of eroded storm volumes and post-storm fluxes revealed that outer shoreface storm deposits would generally be eroded and reworked back into the system in ~ 5.4 y (Fig. 5c), or just longer than the post-storm survey interval of ~ 4.75 y. Importantly, this sediment does not necessarily end up in the zone or the environment from which it originated (Fig. 4d). These data demonstrate that variations in barrier island geomorphology and human interventions contribute to non-systematic patterns of post-storm behavior. Moreover, recovery processes proceed slowly despite significant additions of sediment.

## Discussion

Volume changes and sediment fluxes varied between storm and post-storm periods and with barrier geomorphology, demonstrating that antecedent barrier geomorphic state influences system response over interannual time scales. At the barrier-system scale, storm fluxes were 10 times greater than post-storm fluxes, meaning, in the absence of human interventions, recovery timelines across dune, beach, and inner shoreface environments could exceed 10 y, far longer than typical post-storm observational efforts^25,38,53^ and, in some cases, storm return intervals^60^. This makes storm recovery challenging to quantify along and across barrier systems, particularly in the absence of sustained monitoring, and underscores the utility of the observations made here.

**Fig. 6:**
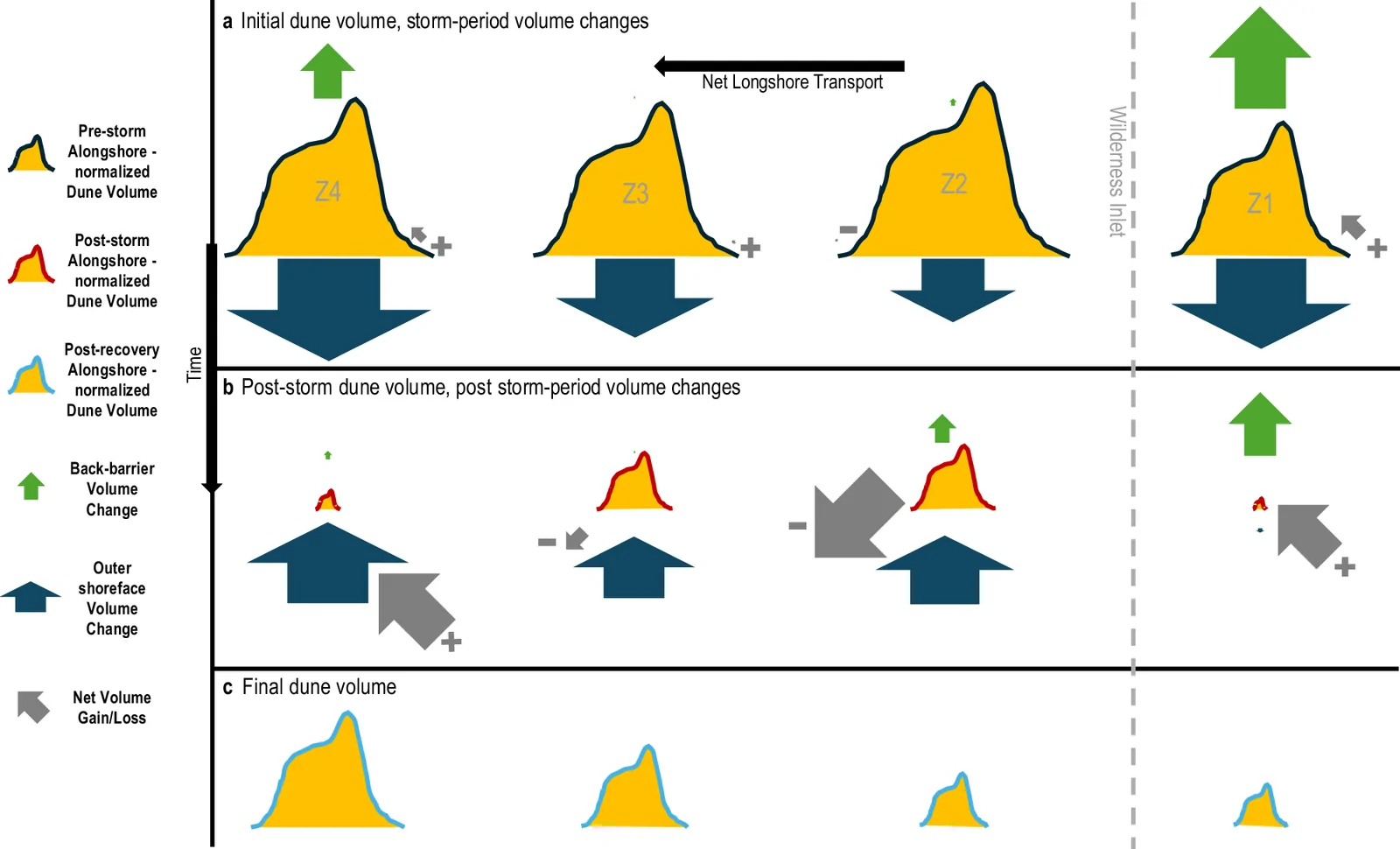
Summary schematic of storm- and post-storm period responses and geomorphic impacts. Alongshore-normalized dune-environment volumes (yellow shape) prior to Hurricane Sandy (**a**, black outline), after the storm period (**b**, red outline), and at the end of the period of observation (**c**, light blue outline). Initial sizes (**a**) are proportional to the observed alongshore geomorphic variability in dune environment volume. Changes in size between panels (**a** to **b** and **b** to **c**) are proportional to measured alongshore-normalized volume changes in the dune environment during the storm-period and post-storm period, respectively. The magnitudes and directions of back-barrier and outer shoreface volume changes are indicated with green and dark blue arrows, respectively. The magnitudes and directions of net volume gains (+) and losses (-) are indicated with gray arrows. Arrow sizes are proportional to the magnitude of the volume changes, whereas arrow orientation indicates direction.

Critical to predicting long-term barrier island longevity is understanding the relative contributions of landward sediment fluxes, which are episodic but maintain barrier resilience over millennial time scales^3,4^. Our observations show that overwash fluxes, with some modulation by pre-storm dune crest heights in the study area^40^, were not the dominant storm flux (Figs. 5a, 6). Even in Zone 1, with the smallest average dune heights and where large washover sheets were observed, washover accumulation accounted for only 29% of all storm period volume deposited. This suggests that overwash fluxes measured in barriers with transgressive morphology should be considered maxima. Even when significant overwash is observed, less than a third of eroded sediment contributes to the constructive washover process that promotes long-term barrier resilience.

In addition to storm-driven washover, our approach captured the inlet-mediated landward flux contribution after the storm in two ways. First, dramatic post-storm losses downdrift of the inlet (Zone 2) did not result in similar magnitude post-storm sediment accumulation in Zone 3 (Figs. 2b, 3b–d, 6). This, coupled with the fact that a large flood tidal delta formed during the period of observation, suggests that some sediment volume released from the island and shoreface in Zone 2 (Fig. 4c) moved landward^61^. Based on modeled updrift and downdrift partitioning of sediment fluxes around inlets^61^, we estimate that ~ 40% the sediment lost from the barrier and shoreface downdrift of the inlet (Zone 2) contributed to the growth of the flood tidal delta. Comparing the estimated flood tidal delta contribution to measured inlet-adjacent washover volumes (Zones 1 and 2)^31^, we find that ~ 67% of the volume transferred landward in the post-storm period was contributed by the inlet, much greater than modeled estimates for wave-dominated barriers^31^. This inlet-related transport is two times the net volume of sediment contributed via washover in adjacent island environments and 1.6 times the net washover volume measured across all AOIs. Second, inlet-induced downdrift dune erosion in Zone 2 resulted in almost four times as much washover volume in the post-storm period compared to the storm period (Figs. 3, 6). This demonstrates how changing barrier geomorphic state dramatically shifts the relevance of barrier-system sediment reservoirs. The combined result of inlet-related landward sediment transport and increased post-storm period washover is an investment in the longer-term resilience of the barrier that would not have happened via washover alone. The large, stable dunes in the modern system, which have historically reduced back-barrier sediment supply, reflect the indirect influence of humans and represent a shift away from the island’s dynamic, inlet-dominated past^26^. These results suggest that when possible, allowing storm-generated breaches to grow into inlets—especially where downdrift geomorphic capital is high^12^—may help offset deficits in landward fluxes generated by unnaturally large dunes. Further, these observations help quantify why suppressing inlet formation on barriers increases their vulnerability to SLR^26,31^.

The unexpectedly small contribution of washover to landward fluxes during the storm period was accompanied by relatively sizeable seaward fluxes even when storm size and relative island position were optimized to drive overwash (see Methods). This was somewhat surprising, since previous work focused seaward of the 10 m water depth showed little storm-related change on the shoreface outside of bedform migration^62^. Measured volume changes in this study were significant, highlighting the critical need for observations that span the upper and lower shoreface to fully understand sediment exchanges between coastal environments^38,63^. Across all geomorphologies, sediment fluxes to the outer shoreface were more than five times the fluxes to the back barrier, exceeding reported volumes lost from outwash events^36^ (Fig. 3a). In the transgressive end member of the system, where the highest overwash flux was observed, flux to the outer shoreface during the storm period was still ~ 2.5 times the flux to the back-barrier, demonstrating that events that induce washover are also very efficient at moving sediment seaward. This suggests that shoreface storm deposits and how quickly they are reworked back into the terrestrial system could significantly alter post-storm barrier behavior over annual to decadal time scales^64^.

Importantly, our results indicate that shoreface storm deposits are not moving directly onshore efficiently as has been observed in other systems where, over a range of time scales, storm-related shoreface deposition returns to the terrestrial system^38,63,65^. After almost five years and across all AOIs, 18% of storm-period sediment remained in the outer shoreface. Assuming all of what eroded was transported landward, only 33% of the post-storm outer shoreface sediment eroded could have resulted in deposition in other barrier environments. Since post-storm period fluxes indicate that the outer shoreface deposit should have completely eroded in ~ 5.4 y (Fig. 5c), this is not wholly related to survey timing. Rather, this suggests that almost half of the storm-period sediment deposited in the outer shoreface in our AOIs was lost to alongshore transport or beyond our boundaries (e.g., further landward or seaward). Our results cannot speak to whether, over longer timescales, these losses will eventually feed barrier environments downdrift of our AOIs or if these sediments will ultimately be permanently sequestered offshore as shelf deposits^66^, representing a net loss to the system. But these results do suggest that barrier system recovery after extreme storms differs from that of lesser storms^65^. It is possible that extreme storms are drivers of net offshore barrier losses, similar to observed post-outwash barrier behavior^36^, since larger waves and higher water levels may transport sediments further offshore, unable to be remobilized by subsequent smaller, more frequent storms. Regardless, these losses further extend post-storm recovery lags induced by inherently large differences in flux magnitudes between storm and post-storm periods, potentially making the barrier less resistant to storms in the short term^36^ and more vulnerable to SLR in the long-term^64^. Taken together, these results suggest that the effects of sea-level rise in geomorphically complex barrier systems may not be offset by shoreface contributions in the same way predicted for embayed coastal systems, where storm erosion and post-storm accretion and decadal trends have been shown to be more reciprocal and net positive^38,63^.

Apart from the spatial variability in storm-period back-barrier and outer shoreface volume changes, there was another notable deviation from AOI-wide relative sediment flux contributions during the storm period, which cannot be explained by dune height variations alone^40^. First, the greatest flux from the beach was measured in transgressive Zone 1 (Fig. 5a). Conversely, in elongational Zone 4, we measured the lowest storm-period flux from the beach and the highest fluxes from the inner shoreface and to the outer shoreface (Fig. 5a). However, we don’t see similarly elevated shoreface fluxes in Zones 2 and 3 (Fig. 5a) despite the similarity in dune heights between the AOIs representing Zones 2-4 (Fig. 2e). Previous work has shown increasing shoreface sediment volume from east to west along the island (Supplementary Fig. S3)^67^. Though this applies to the Holocene package (Supplementary Fig. S3a), more relevant to this discussion is a unit overlying it, which is thought to represent reworked Holocene sediments and for which sediment volume also increases from east to west (Supplementary Fig. S3b and c)^67^. In combination with our observations, this suggests that higher shoreface sediment availability is related to higher storm-period shoreface fluxes. Additionally, our results suggest that localized deficits in the shoreface reservoir can contribute to more dramatic changes in the terrestrial component of the system during storms, since we measured higher beach fluxes where shoreface sediment supplies are lower. These observable connections between shoreface sediment availability and the magnitude of sediment fluxes into and out of barrier environments extend knowledge about connections between shoreface geology and long-term barrier island response^42,43,46,68^ to shorter, storm-related time scales and may help explain previously identified correlations between decadal to centennial-scale shoreline erosion trends and lower shoreface sediment availability^43,69^.

Shoreface contributions were much harder to tease out in the post-storm period since morphologic trajectories were highly influenced by human interventions and losses to alongshore transport or beyond the survey boundary, highlighting how connectivity in the alongshore domain dominated in the post-storm period (Fig. 6). One way this manifests in our observations is as lags between erosion of the outer shoreface and recovery of inner shoreface and island environments, confirming suspected differences in the timescales governing terrestrial and shoreface processes^55,70^. This finding, combined with differing flux magnitudes between the inner and outer shoreface, demonstrates that linear-style shorefaces favored in some reduced complexity models of barrier response to SLR^4,29,31,56^ could overestimate shoreface response rates and underestimate barrier vulnerability. Approaches that treat the upper and lower shoreface separately may better reflect observations and more accurately represent known lags in upper and lower shoreface response^42,54,55,70^.

Across all AOIs, net losses were driven by terrestrial deficits (Fig. 4c) and overall recovery was low, amounting to only 16% of what was lost from beach, dune, and inner shoreface environments during the storm period (Fig. 4d). This was driven by redirection of sediment supply into Wilderness Inlet and to the back-barrier in Zone 2, arguably an investment in long-term barrier resilience^14^. If Zone 2 is removed from the analysis, there is an increase in percent overall recovery from east to west (Zone 1 to Zone 4; Figs. 4d and 6c) following the net longshore sediment transport direction, transitions in terrestrial geomorphology^26^, and shoreface sediment availability (Supplementary Fig. S3)^67^. Furthermore, recovery in dune, beach, and inner shoreface environments would have measured just over 80% instead of less than 20%. This highlights the scale of the geomorphic cost inlets extract from barrier islands in the short-term as they help build barrier resilience for the future. Still, the fact that 100% recovery of storm-eroded sediment wasn’t achieved despite sediment additions four times greater than net AOI losses underscores the scale of extreme storm impacts and brings into question how we will prioritize coastal sediment management efforts in the context of diminishing^27^ and increasingly necessary^71^ sand resources.

Perhaps informative to that end, we found that position in the alongshore barrier geomorphic continuum relative to natural and anthropogenic sediment supplies influenced the extent of overall recovery and its cross-shore distribution. Even without human intervention, the elongational zone may have been best poised to recover quickly because alongshore and shoreface sediment supplies are maximized. Direct nourishment likely hastened the process. The combination resulted in net sediment accumulation and overall recovery of 161% (Fig. 4c, d). In contrast, the transgressive zone had the lowest overall percent recovery despite anthropogenic increases in updrift sediment supply and reduced downdrift losses due to the growth of the Wilderness Inlet ebb tidal delta, which demonstrates how barrier morphologies respond differently to increases in sediment supply (Fig. 4d). This zone also recorded the highest flux to the back-barrier environment (Fig. 5b), which suggests that, for transgressive systems, rapidly building artificial dunes after storms may put natural sediment transport pathways at risk^72,73^. Taken together, these insights show how understanding feedbacks between barrier geomorphic state and recovery timelines may better help coastal managers strategically place valuable sediment resources in barrier systems to maximize near-term storm resilience while still maintaining sediment transport processes that promote barrier island longevity in the face of rising sea levels.

Finally, we do not observe an island-scale transition toward one new geomorphic state from this extreme storm (Fig. 6)^14^. Instead, our work captures a reconfiguration of barrier sediment reservoirs, particularly the dunes. Using dune environment volume as a proxy for morphologic resistance^14^, we find resistance to landscape change is reduced and proportional to the alongshore morphology gradient (Fig. 6c). At the zone scale, however, the event forces a crossing of landscape thresholds, from barrier to inlet, causing downdrift sediment distribution and flux changes (Fig. 6, Z2). Zone 2 storm and post-storm back-barrier fluxes are approximately equal (Fig. 5b), so an extreme storm flux now happens annually. This sediment storage transition from the dune to the back-barrier indicates an inlet-related downdrift geomorphic state change from historically progradational to transgressional. Despite this local geomorphic state shift, the general function of the barrier is unchanged, demonstrating how zone-scale geomorphic capital can absorb perturbation impacts and contribute to short-term island-scale barrier resilience^12^. The implications of these findings for longer-term barrier behavior remain to be tested. Modeled^12,64^ and observed (this study) system lags, combined with further reductions in resistance resulting from predicted increases in storm intensity^5^ and rates of SLR^7^ could propel the system to an island-scale state shift similar to observed local-scale behavior. Sustained measurement of barrier island sediment partitioning around and after extreme events provides critical context for modeling efforts that seek to predict when feedbacks between barrier morphology, process lags, and human interventions could lead to island-scale geomorphic shifts. Both are invaluable for informing coastal barrier adaptation strategies for an increasingly uncertain future.

## Methods

### Storm characteristics & geomorphologic setting

Hurricane Sandy made landfall on 29 October 2012 as a post-tropical cyclone, approximately 190 km southwest of Fire Island, NY, a barrier island on the east coast of the United States (Fig. 2)^58^. Extreme water levels and wave energy associated with the storm breached Fire Island in several places although only Wilderness Inlet was left open to evolve naturally (Fig. 2). Fire Island is a wave-dominated barrier with monthly wave heights and periods averaged over the last 40 years of 1.1 m and 7.2 s, respectively (ST 63114; https://wisportal.erdc.dren.mil; accessed 3/3/2026) and a tidal range around 1 m (Leatherman, 1985). However, the island often experiences wave conditions that exceed long-term averages during extratropical storms that occur during the months of November-May and sometimes during tropical cyclones that occur in the Atlantic basin during the months of June-November (Supplementary Fig. S1, S2). During extratropical cyclones, significant wave heights can exceed 5 m but rarely for more than about 15 h. Though winds at landfall did not exceed Category 1 on the Saffir-Simpson Scale, the size of Hurricane Sandy generated significant wave heights that exceeded 5 m for more than 40 h causing catastrophic storm surge along the New Jersey and New York coastlines^58^. The combination of extreme surge and runup and the fact that Fire Island was in the northeast quadrant of the storm affected profound geomorphic changes, including breaching in three places and washover deposition along nearly half (~ 47%) of the island^74^.

A substantial amount of preceding research on Fire Island, particularly concerning alongshore variations in subaerial geomorphology^26,75^ and offshore geology^46^, provided an opportunity to quantify the effects of island geomorphic variability on sediment exchanges between and within different geomorphic environments. Combining three topographic and three bathymetric surveys collected over five years, we explore the storm and post-storm behavior along and across the barrier island. Terrestrial elevation changes were captured using airborne lidar collected in May 2012^76^ (pre-storm), November 2012^77^ (6 days post-storm), and September 2017^78^. Shoreface elevation changes were captured with topo-bathymetric lidar in October 2012^79^ (pre-storm) and acoustic, boat-based soundings in October 2013^80^ and June 2018^81^ (post-storm). Though the timing of terrestrial and submerged elevation surveys were not exactly in phase, the second post-storm surveys were conducted at points in time consistent with patterns of upper beach recovery as measured from beach profiles from Fire Island^53^ and within the ranges for barrier recovery for U.S. east and gulf coast barriers^52,82^.

The spatial breadth of data coverage in the alongshore domain allowed us to quantify changes in both terrestrial and submerged environments representing four different geomorphologic configurations, or states (Fig. 2b). Based on the interpretations of Ciarletta and others^26^, the easternmost geomorphic zone, Zone 1, is transgressional, having migrated landward over the last several hundred years. It is bound to the east by Moriches Inlet and to the west by Wilderness Inlet, which opened during Hurricane Sandy. This part of the island features an often discontinuous foredune and the lowest average dune crest elevations on the island (Fig. 2c, d). Zone 2 includes the Otis Pike High Dunes Wilderness at Fire Island National Seashore, and its eastern boundary is Wilderness Inlet. Here, the island was historically progradational and has a continuous foredune with some of the highest elevations on the barrier (Fig. 2d). It is backed by geomorphologically complex, often discontinuous secondary and tertiary ridges (Fig. 2c). Zone 3 is interpreted to be geologically progradational, meaning that it has been relatively stable recently, but experienced a period of progradation > 600 years ago. It has a wide, amalgamated, and continuous foredune in the eastern part of the zone and a narrow (Fig. 2c), continuous foredune in its western reaches. On average, Zone 3 dune crest elevations are not as high as those in Zone 2 but still exceed the island average (Fig. 2d). Lastly, westernmost Zone 4 is elongational, having grown westward over the last several hundred years^26,75^. Because of this extension, average foredune elevation in Zone 4 is the second lowest along the island (Fig. 2d). Due to varying degrees of direct human modifications of island morphology within each zone in the form of infrastructure and nourishment, we reduced the scope of the analysis to focus on predominantly natural areas greater than 1 km in alongshore length, resulting in six areas of interest (AOIs). This resulted in at least one AOI in each of the four geomorphic zones (Fig. 2b).

### Digital elevation models & survey and volumetric uncertainty

Digital elevation models (DEMs) were created from each topographic and bathymetric dataset using Blue Marble Global Mapper (v.23.2) software. Because bathymetric coverage was less dense than terrestrial lidar coverage, bathymetric DEMs were interpolated using a Triangulated Irregular Network (TIN) with a grid-cell size of 50 m, whereas topographic DEMs had a grid-cell size of 1 m. Though higher resolution shoreface data would be better, these data are sufficient for the analysis presented here since we are primarily concerned with whether volume generally increased or decreased in one environment or another, which does not require detailed morphologic information that would necessitate higher resolution data (e.g., sand bar and trough shape). So, the nature of the analysis lends itself well to sparse data, potentially increasing the portability of the methodology. To quantify uncertainty, we first calculated the survey uncertainty for each DEM modified from the methods of Maloney and others^83^. For the terrestrial data, the Fundamental Vertical Accuracy (FVA; Supplementary Table S15) of each DEM is reported in the associated metadata for each lidar survey as:

1 $${FVA}=\,{{RMSE}}_{z}\times \,1.96$$

where *RMSE*_*z*_ is the vertical root mean square error in meters determined using calibration control points^76^. For submerged data, we calculate the Survey Uncertainty (SU; Supplementary Table S16) of each dataset as:

2 $${SU}=\sqrt{({({{E}_{{acq}}}^{2})}+\,{({{E}_{{grid}}}^{2})})}$$

where ${E}_{{acq}}$ is the acquisition error reported in the associated metadata for the 2012 and 2018 surveys in meters and ${E}_{{grid}}$ is the grid error in meters (Supplementary Table S16). Grid error is the root mean squared error calculated by gridding elevations with 10% of the soundings removed and then comparing the elevations at those points to the interpolated value at that same location as follows:

3 $${E}_{{grid}}=\sqrt{\frac{\sum {({z}_{{obs}}-{z}_{{interp}})}^{2}}{n}}$$

where ${{z}_{{obs}}}$ is the elevation of the removed soundings, ${z}_{{interp}}$ is the interpolated value at the location of the removed elevation, and n is the number of points removed. Metadata available for the October 2013 dataset was limited, and it was also the survey with the sparsest coverage in the undeveloped sections of the island. Therefore, ${E}_{{acq}}$ and ${E}_{{grid}}$ were estimated differently. ${E}_{{acq}}$ for October 2013 was calculated as the average reported ${E}_{{acq}}$ for three post-storm acoustic bathymetry surveys (0.15 m). ${E}_{{grid}}$ for October 2013 was estimated by using the 2018 data to simulate the sparse coverage of the 2013 survey. First, elevations from the denser 2018 survey were extracted from along or near the 2013 transects. Next, a 50-m grid was created using this downsampled 2018 data. Then, this grid was used to extract interpolated elevations (${{z}_{{interp}}}$) at all 2018 measured elevation locations (${z}_{{obs}}$). Finally, an estimate of ${E}_{{grid}}$ for the 2013 survey could be calculated using Eq. (3). FVA and survey uncertainties for each DEM are shown in Supplementary Tables S15, S16, where survey uncertainties for the 2013 survey are reported for each AOI as detailed above.

Since we difference individual surveys to calculate volume changes (Supplementary Figs. S4–S9), we need an estimate of volumetric uncertainty (VU; Supplementary Tables S17, S18), which we calculated as follows:

4 $${VU}=\sqrt{\left(\left({{{FVA}}_{t1}}^{2}\right)+\left({{{FVA}}_{t2}}^{2}\right)\right)}-{{\rm{OR}}}-{VU}=\sqrt{\left(\left({{{SU}}_{t1}}^{2}\right)+\left({{{SU}}_{t2}}^{2}\right)\right)}$$

where *t*_*1*_ and *t*_*2*_ indicate surveys from two different time periods. To determine the fraction of the total elevation range that can be attributed to uncertainty, we normalize the volume uncertainty by the average elevation range surveyed between the two surveys^83^:

5 $${NVU}=\frac{{VU}}{{\bar{z}}_{\max }-{\bar{z}}_{\min }}$$

where VU is the volume uncertainty from Eq. (4) and ${\bar{z}}_{\max }$ and ${\bar{z}}_{\min }$ are the average maximum and minimum elevations between the two surveys measured in meters, respectively. To calculate the percent volume error, we multiply the normalized volume uncertainty by 100:

6 $$VE_\%=NVU\times100$$

Volumetric uncertainty, normalized volume uncertainty, and volumetric percent error for each survey pair are shown in Supplementary Tables S17, S18, and survey uncertainties for the 2013 survey are reported for each AOI. Volumetric percent error for terrestrial environments was 1.2% and 3.1% for the storm and post-storm periods, respectively (Supplementary Table S17). Maximum volumetric percent error for submerged environments was 4.9% and 5.1% for the storm and post-storm periods, respectively (Supplementary Table S18).

### Delineating barrier island environment boundaries

To quantify how volume changes varied in the cross-shore domain, each AOI was partitioned into five barrier island environments: outer shoreface, inner shoreface, beach, dune, and back-barrier (Fig. 1). Since an inlet formed during the period of observation, shoreface observations include any deposition associated with the formation and growth of the ebb tidal delta. The offshore limit of the outer shoreface was defined as the shallowest offshore survey extent, which corresponds to the pre-storm topo-bathymetric lidar footprint (October 2012) and was, on average, − 8.6 m. This offshore boundary did not always extend to the estimated depth of closure (DOC) when calculated based on a 30-yr time series of wave data (− 9.25 m^84^) nor the estimated DOC during Sandy (~ − 10 m). However, our measurements do not span 30 years, and our analysis explores the post-storm period as well, so a more representative metric was used. To determine this, we calculate the DOC for the post-landfall period of observation (e.g., 30 October 2012 to 1 July 2018) using the record of hourly waves measured from NDBC buoy 44025 (https://www.ndbc.noaa.gov/station_page.php?station=44025; Supplementary Fig. S2c, S2d). First, we determined when waves exceeded 2 m, when wave conditions are more likely to move sediments in deeper water. We then used the Hallermeier, 1978 equation^85^ to calculate the DOC for each wave measurement and then calculated the average and standard deviation. We found that the estimated DOC plus one standard deviation^85^ is − 6.6 m for the period of observation. The average offshore data limit (− 8.6 m) exceeded this estimate by about 2 m, providing greater confidence that any underestimation of outer shoreface volume changes was minimal. Furthermore, if we didn’t have this limitation, outer shoreface volumes would be likely to increase even beyond what we already measured during the storm period, which would reinforce the patterns we are seeing with the data limitations we have and would be unlikely to change the observed trends.

Elevation contours were used as the boundaries between outer shoreface, inner shoreface, beach, and dune environments. The boundary between inner and outer shoreface environments was the DOC plus one standard deviation calculated from all wave conditions spanning the period of observation^85^ (as described above; Supplementary Fig. S2c, d), or the − 4.05 m contour (Fig. 1). The shoreline, defined here as operational mean high water^86^ (MHW; 0.46 m, NAVD88), serves as the landward limit of the inner shoreface and the seaward limit of the beach environment. The boundary between beach and dune environments was the upper beach change envelope (UBCE), which represents the landward limit of wave incursion during five storms between 2005 and 2012^53^ (2.9 m, NAVD88).

Because of the difficulty in readily defining geomorphic boundaries across large spatial domains in back-barrier environments^87^ (e.g., the dune heel) and increases in airborne lidar elevation uncertainty related to back-barrier vegetation^88^, we did not use single elevation contours to define the landward limits of dune and sandy back-barrier environments. First, the landward boundary of the dune environment for the storm period was extracted from topographic change DEMs as the boundary between measured erosion and accretion (Supplementary Fig. S4–S9), what we refer to as the dune limit, similar to an approach used by Bauer and others^89^. Using ESRI ArcMap, an elevation change map was created by subtracting pre- and post-storm lidar (May 2012-November 2012). The resulting map was contoured, and the − 0.15 m elevation change contour (seaward) was chosen to conservatively account for the average reported error of the lidar datasets (±0.13 m) and decrease the possibility of overestimating dune loss. Using this methodology allowed us to consistently capture pre-storm maxima in dune crest elevations (Fig. 2 and Supplementary Figs. S4–S9).

Second, to define the landward limit of the sandy back-barrier environment for the storm period, we extracted storm washover extents from SPOT 5 satellite imagery (©Center National d’Etudes Spatiales [CNES]) acquired on 1 Dec. 2011 and 12 Sep. 2012 (pre-storm) and 3 Dec. 2012, 8 Apr. 2013, and 3 May 2013 (post-storm). We knew that post-storm back-barrier volume changes would be difficult to resolve given lidar elevation uncertainties related to vegetation^88^, so this approach allowed us to tailor our analysis extents to unvegetated areas, thereby maximizing the change we could resolve. After being clipped to the study area, image-to-image geo-correction was performed using ERDAS IMGINE® Autosync Workstation using National Agriculture Imagery Program (NAIP) reference aerial imagery acquired May-2011 and Jun-2013. The root-mean square error (1 standard deviation) for the geo-corrected images ranged from 0.13 ± 0.067 pixels (3-May-2013) to 0.154 ± 0.090 pixels (3-Dec-2012). Modifying the workflow from Bernier and others^90^, back-barrier sand extents (e.g., washover extents) were mapped by: (1) calculating the normalized difference water index (NDWI)^91^ using bands 1 (green) and 3 (near infrared); (2) applying automatic thresholding^92^ to each NDWI image to create a binary land-water raster; (3) masking water areas from SPOT 5 band 2 images and again applying automatic thresholding^92^ to separate the images into sand and non-sand extents; and (4) extracting landward sand extent (e.g., the sand-vegetated boundary) by contouring the masked band 2 images. Sand extents were visually inspected, and minor manual editing was performed to remove spectrally bright vegetated areas. Washover extents related to Hurricane Sandy were identified by comparing differences between pre- and post-storm sand extents.

Because barriers are dynamic, barrier island environment boundaries were not static between storm and post-storm periods, apart from the seaward limit of the outer shoreface (e.g., the smallest survey extent). For this analysis, the position of elevation-based contours such as the inner depth of closure, the MHW shoreline, and the UBCE were updated based on the average elevations from post-storm surveys. The post-storm dune limit was based on dune geomorphology and targeted the part of the barrier in which dune building was most likely to occur, or some distance landward of the UBCE, allowing us to capture any early or intermediate phase of dune building in the post-storm period^93^. First, we calculated the average cross-shore width of the storm-period dune environment from 10-m spaced transects (${x}_{d}$; Supplementary Fig. S10.). To define the dune limit for the post-storm period, we applied a buffer of that same average width (${\bar{x}}_{d}$; Supplementary Fig. S10) to the post-storm period UBCE. Where the post-storm period dune limit did not exceed the storm-period dune limit (e.g., where washovers projected into the back-barrier), the dune limit was extended to match the storm-period boundary (Supplementary Fig. S10). This ensured that foredune development or elevation gains in any part of the area that had been eroded during the storm was captured. A similar approach was used for the back-barrier environment. The average cross-shore width of the storm-period back-barrier environment was calculated using 10-m spaced transects (${x}_{{bb}}$; Supplementary Fig. S10), and a buffer of the same average width was applied to the storm-period back-barrier boundary (${\bar{x}}_{{bb}}$;Supplementary Fig. S10). Where no sand extent was identified in the storm period, the back-barrier buffer was applied to the post-storm period dune limit (Supplementary Fig.S10). The application of our post-storm boundaries worked well in capturing post-storm changes except for the part of the island immediately downdrift of the inlet, which occupied a more landward footprint relative to the pre-storm period. This led to an underestimation of deposition in the back-barrier environment. Therefore, for the eastern 1.3 km of Zone 2, we extended the limit of the back-barrier environment to include any deposition above 0.41 m, which was the volumetric uncertainty for the differenced lidar surveys (Supplementary Table S17).

### Calculating storm & post-storm period volume changes

With alongshore and cross-shore boundaries defined for each barrier island environment for the storm and post-storm periods, volume changes in each AOI and environment could be quantified. Volumes were calculated with the ‘Measure Volume Between Surfaces’ tool in Global Mapper software and simultaneously clipped to barrier island environment extents for each AOI (Supplementary Figs. S4–S9).

We used several calculations to characterize volumetric changes. Since the AOIs varied in alongshore extent, we compared volume change magnitudes between geomorphic zones using alongshore-normalized volume change (m^3^ m^-1^; Supplementary Table S3, S4) which we calculated as:

7 $$\frac{{{Volume}\,{Change}}_{{z_x}_{,e}}}{{{Alongshore}\,{length}}_{{z_x}}}$$

where ${{z_{x}}}$ represents the AOI or AOIs in the geomorphic zone and *e* represents one of the barrier island environments (e.g., outer shoreface, inner shoreface, beach, dune, back barrier). Zone 3 encompassed three AOIs, so the volumes and alongshore distances across all AOIs were summed. We also calculated the distribution of volume accumulated in or eroded from various barrier island environments (%; Supplementary Tables S5, S6) which we calculated as:

8 $$\left(\frac{{{Volume\; deposition}}_{{z_x}_{,e}}}{{{Volume\; deposition}}_{{z_x}_{,total}}}\right)\times 100\,{{\rm{ -OR- }}}\left(\frac{{{Volume\; erosion}}_{{z_x}_{,e}}}{{{Volume\; erosion}}_{{z_x}_{,total}}}\right)\times 100$$

where ${{z_{x}}_{,total}}$ denotes the total accumulation or erosion in a particular AOI in the geomorphic zone.

For the storm period, we were interested in what percentage of washover volume could be attributed to dune volume loss (%; Supplementary Table S7), and we calculated this as:

9 $$\left(\frac{{{Volume}\,{deposition}}_{{z_x}_{,back barrier}}}{{{|Volume}\,{erosion}}_{{z_x}_{,dune}}|}\right)\times \,100$$

For a given AOI, we wanted to understand the extent to which accumulation was balanced by erosion to determine where there had to be a net import or export of sediment to arrive at the observed volume changes. We calculated percent sediment gain relative to loss (%; Supplementary Table S8) as:

10 $$\left(\left(\frac{{{Volume}\,{deposition}}_{{z_x}_{,total}}}{{{|Volume}\,{erosion}}_{{z_x}_{,total}}|}\times 100\right)\right)-100$$

The closer the percentage is to 0, the better the balance between erosion and deposition in that AOI. We interpret negative percentages (erosion > accumulation) as losses to longshore transport or beyond the AOI extent. We interpret positive percentages (accumulation > erosion) as net import, with the addition of sediment from somewhere outside the AOI boundaries.

We calculated net volume change resulting from the storm and post-storm periods as:

11 $${{Volume}\,{Change}}_{{z_x}_{,e,storm}}+{{Volume}\,{Change}}_{{z_x}_{,e,post-storm}}$$

and then summed those values for terrestrial (beach, dune, back barrier) and shoreface (inner, outer) environments to understand how net changes over the period of observation manifested in subaerial and subaqueous components of the barrier system (Supplementary Table S9).

To understand to what extent barrier island environments that eroded during the storm period accumulated sediment during the post-storm period, we calculated percent recovery (%; Supplementary Table S10) for each environment in each AOI by dividing post-storm net volume change by the net volume change observed during the storm period:

12 $$\left(\frac{{{Volume}\,{deposition}}_{{z_x}_{,e,post-storm}}}{|{{Volume\,}{erosion}}_{{z_x}_{,e,storm}}|}\right)\times 100$$

To calculate sediment fluxes for the storm period (m^3^ m^-1^ y^-1^; Supplementary Table S11), we divide the alongshore-normalized volume from Eq. (7) by a storm-return interval. Brenner et al. ^53^ identified 5 storms of record (including Sandy) over 7 years, resulting in a storm-return interval of 1.4 y. The storms of record included hurricanes and large extratropical storms, accounting for any event that could generate overwash and offshore-directed fluxes. To calculate sediment fluxes for the post-storm period (m^3^ m^-1^ y^-1^; Supplementary Table S12), we divide the alongshore-normalized volumes from Eq. (7) by the survey interval. For terrestrial environments the survey interval was 4.83 y and for shoreface environments the survey interval was 4.68 y.

13 $$\frac{{{Equation}\,7}_{{z_x}_{,e}}}{{Time}\,{interval}}$$

Finally, we wanted to understand how long it would take to achieve net zero volume change in outer and inner shoreface, beach, and dune environments based on measured post-storm fluxes. We calculated these recovery times (y; Supplementary Table S14) as:

14 $$\frac{\left|{{Net}{Volume}\,{Change}}_{{z_x}_{,e,storm}}\right|/{{Alongshore}\,{length}}_{{z_x}}}{{{Equation}\,13}_{{z_x}_{,e,post-storm}}}$$

### Reporting summary

Further information on research design is available in the Nature Portfolio Reporting Summary linked to this article.

## Supplementary information

Supplementary Information

Reporting Summary

Transparent Peer Review file

## Data Availability

All source data are publicly available via the citations provided in the methods. All data generated from those source data and analyzed in this study are included in the supplementary information.
