# Supplementary material for: Barrier island geomorphic complexity drives storm and post-storm response
Source: Nat Commun. 2026 Sep 24;17:10172. doi: 10.1038/s41467-026-77143-6 (PMC13612595; doi:10.1038/s41467-026-77143-6)
Supplement: Supplementary file 1 [file 41467_2026_77143_MOESM1_ESM.pdf]

Barrier island geomorphic complexity drives storm and post-storm response

Jennifer L. Miselis[1a], Noreen A. Buster[1], Daniel J. Ciarletta[1], Julie C. Bernier[1], Emily A. Wei[2], Rose V. Palermo[1]

[1]U.S. Geological Survey, St. Petersburg Coastal and Marine Science Center, 600 4th St. S, St. Petersburg, FL 33701

[2]Louisiana State University, Department of Geology and Geophysics, E235 Howe Russell Kniffen, Baton Rouge, LA 70803

[a]corresponding author, jmiselis@usgs.gov

Supplementary Information

Disclaimer: Any use of trade, firm, or product names is for descriptive purposes only and does not imply endorsement by the U.S. Government.

Table S1. Dune Crest Height Elevation Statistics – By Zone (Figure 2d)

Zone	Average Dune Height (m, NAVD88*)	Standard Deviation in Dune Height (m)	Minimum Dune Height (m)	Maximum Dune Height (m)
1	5.0	1.0	2.6	8.4
2	6.3	1.2	3.1	10.0
3	5.8	1.0	3.5	10.3
4	5.2	1.3	1.2	8.6

*North American Vertical Datum, 1988

Table S2. Dune Crest Height Elevation Statistics – By Area of Interest (AOI) (Figure 2e)

Zone (AOIs)	Average Dune Height (m, NAVD88*)	Standard Deviation in Dune Height (m)	Minimum Dune Height (m)	Maximum Dune Height (m)
1 (6)	3.9	1.1	2.6	5.9
2 (5)	6.4	1.1	3.4	10.0
3 (2-4)	6.0	0.9	3.5	10.3
4 (1)	6.1	1.1	3.5	8.3

*North American Vertical Datum, 1988

Table S3. Alongshore-normalized Volume Changes, Storm-period (Figure 3a)

Barrier Environment	Zone 1 (m^3 m^{-1})	Zone 2 (m^3 m^{-1})	Zone 3 (m^3 m^{-1})	Zone 4 (m^3 m^{-1})	All AOIs (m^3 m^{-1})
Back-barrier	60.7	4.6	1.3	32.9	99.6
Dune	-66.1	-58.7	-52.0	-73.7	-250.5
Beach	-36.3	-15.1	-15.6	-13.9	-80.8
Inner shoreface	-58.4	-38.0	-55.5	-81.9	-233.9
Outer shoreface	146.3	102.7	129.2	167.0	545.1

Table S4. Alongshore-normalized Volume Changes, Post-storm-period (Figure 3b)

Barrier Environment	Zone 1 (m^3 m^{-1})	Zone 2 (m^3 m^{-1})	Zone 3 (m^3 m^{-1})	Zone 4 (m^3 m^{-1})	All AOIs (m^3 m^{-1})
Back-barrier	37.5	17.2	1.4	5.1	61.2
Dune	17.3	-6.8	13.0	54.6	78.2
Beach	14.0	-24.4	32.6	69.3	91.5
Inner shoreface	45.7	-45.2	27.8	149.0	177.24
Outer shoreface	6.5	-112.3	-99.2	-131.9	-337.0

Table S5. Proportion of Deposition or Erosion, Storm-period (Figure 3a)

Barrier Environment	Zone 1 (%)	Zone 2 (%)	Zone 3 (%)	Zone 4 (%)	All AOIs (%)
Back-barrier	29.3	4.3	1.0	16.5	8.4
Dune	-41.1	-52.5	-42.2	-43.5	-46.8
Beach	-22.6	-13.5	-12.7	-8.2	-13.5
Inner shoreface	-36.3	-34.0	-45.1	-48.3	-39.7
Outer shoreface	70.7	95.7	99.0	83.5	91.6

Table S6. Proportion of Deposition or Erosion, Post-storm-period (Figure 3b)

Barrier Environment	Zone 1 (%)	Zone 2 (%)	Zone 3 (%)	Zone 4 (%)	All AOIs (%)
Back-barrier	31.0	100.0	1.8	1.8	38.9
Dune	14.3	-3.6	17.4	19.7	23.8
Beach	11.6	-12.9	43.6	24.9	20.6
Inner shoreface	37.7	-23.9	37.1	53.6	16.7
Outer shoreface	5.4	-59.5	100.0	100.0	100.0

Table S7. Percent Contribution of Dune Erosion to Overwash (Figure 4a)

Zone (AOIs)	Zone (%)
1 (6)	91.9
2 (5)	7.8
3 (2-4)	2.5
4 (1)	44.7
All AOIs	19.0

Table S8. Percent Net Import (+) or Export (-), Gain relative to Loss, (Figure 4b)

Zone (AOIs)	Storm-period (%)	Post-storm period (%)
1 (6)	28.7	No loss measured
2 (5)	-4.1	-90.9
3 (2-4)	6.0	-24.6
4 (1)	18.0	110.8
All AOIs	5.7	-48.8

Table S9. Terrestrial (back-barrier, dune, beach) and Shoreface (inner and outer) Net Volume Changes and proportions (Figure 4c)

Zone (AOIs)	Terrestrial (m^3)	Shoreface (m^3)	% Terrestrial	% Shoreface
1 (6)	46242.3	237769.4	16.3	83.7
2 (5)	-788621.4	-879852.5	47.3	52.7
3 (2-4)	-122407.6	13930.2	89.8	10.2
4 (1)	139498.5	191578.0	42.1	57.9
All AOIs	-725288.2	-436574.9	62.4	37.6

Table S10. Recovery percentages (Figure 4d)

Zone (AOIs)	Inner shoreface (%)	Beach (%)	Dune (%)	Overall (%)
1 (6)	78.2	38.6	26.2	47.9
2 (5)	-118.8	-162.0	-11.6	-68.4
3 (2-4)	50.0	209.6	25.0	59.6
4 (1)	181.9	500.1	74.1	161.0
All AOIs	10.9	39.4	13.2	15.8

Table S11. Sediment Fluxes, Storm-period (Figure 5a)

Sediment Flux	Zone 1 ($m^3 m^{-1} y^{-1}$)	Zone 2 ($m^3 m^{-1} y^{-1}$)	Zone 3 ($m^3 m^{-1} y^{-1}$)	Zone 4 ($m^3 m^{-1} y^{-1}$)	AOI Average ($m^3 m^{-1} y^{-1}$)
Q,ow	43.4	3.3	0.9	23.5	17.8
Q,d	-47.2	-41.9	-37.2	-52.7	-44.7
Q,b	-25.9	-10.8	-11.1	-9.9	-14.4
Q,iSF	-41.7	-27.2	-39.7	-58.5	-41.8
Q,oSF	104.5	73.3	92.3	119.3	97.4

Table S12. Sediment Fluxes, Post-storm-period (Figure 5b)

Sediment Flux	Zone 1 ($m^3 m^{-1} y^{-1}$)	Zone 2 ($m^3 m^{-1} y^{-1}$)	Zone 3 ($m^3 m^{-1} y^{-1}$)	Zone 4 ($m^3 m^{-1} y^{-1}$)	AOI Average ($m^3 m^{-1} y^{-1}$)
Q,ow	7.8	3. 6	0.3	1.1	3.2
Q,d	3.6	-1.4	2.7	11.3	4.0
Q,b	2.9	-5.1	6.8	14.3	4.7
Q,iSF	9.8	-9.7	6.0	31.9	9.5
Q,oSF	1.4	-24.1	-21.3	-28.3	-18.1

Table S13. Storm-period Sediment Flux Comparative Ratios (Figure 5a)

Sediment Flux Ratios	Zone 1 (non-dimensional)	Zone 2 (non-dimensional)	Zone 3 (non-dimensional)	Zone 4 (non-dimensional)	AOI Average (non-dimensional)
Q,oSF /Q,ow	2.4	22.4	99.2	5.1	5.5
Q,d/ Q,b	2.0	3.9	3.3	5.3	3.1
Q,iSF /Q,b	1.6	2.5	3.6	5.9	2.9

Table S14. Recovery timelines (Figure 5c)

Barrier Environment	Zone 1 (y)	Zone 2 (y)	Zone 3 (y)	Zone 4 (y)	All AOIs (y)
Dune	18.4	n/a	19.3	6.5	14.8
Beach	12.5	n/a	2.3	1.0	5.3
Inner shoreface	6.0	n/a	9.3	2.6	6.0
Outer shoreface	n/a	4.3	6.1	5.9	5.4

Table S15. Terrestrial Datasets-Fundamental Vertical Accuracy

Survey Timing	Survey Date	Fundamental Vertical Accuracy (m)	z_{max} (m)	z_{min} (m)*
Pre-storm	05/2012	0.08	15.1	0.46
Post-storm	11/2012	0.15	14.8	0.46
Post-storm	09/2017	0.38	13.0	0.46

*z_{min} adjusted to analysis boundary of operational mean high water (Weber et al., 2006)

Table S16. Submerged Datasets-Survey Uncertainty

Survey Timing	Survey Date	Acquisition Error, E_{acq} (m)	Grid Error, E_{grid} (m)	Survey Uncertainty (m)	z_{max} (m)*	z_{min} (m)
Pre-storm	10/2012*	0.05	0.25	0.26	-0.59	-20.3
Post-storm	AOI 1, 10/2013^	0.15	0.25	0.29	-0.12	-9.87
Post-storm	AOI 2, 10/2013^	0.15	0.29	0.33	-0.38	-9.7
Post-storm	AOI 3, 10/2013^	0.15	0.27	0.31	-0.36	-8.5
Post-storm	AOI 4, 10/2013^	0.15	0.31	0.34	-0.28	-8.5
Post-storm	AOI 5, 10/2013^	0.15	0.45	0.47	-0.50	-10.9
Post-storm	AOI 6, 10/2013^	0.15	0.58	0.60	-0.71	-7.6
Post-storm	06/2018	0.15	0.20	0.25	-0.43	-18.8

*lidar bathymetry; adjusted z_{max} to average of other surveys
^error was not reported for this survey; E_{acq} estimated as average of all acoustic bathymetry surveys

Table S17. Volumetric Uncertainty-Terrestrial

Period	Survey Pair	Volumetric Uncertainty (m)	$\overline{z}_{max}$ (m)	$\overline{z}_{min}$(m)	Normalized Volumetric Uncertainty (non-dimensional)	Volumetric % Error
Storm	05/2012-11/2012	0.17	15.0	0.46	0.012	1.2 %
Post-storm	11/2012-09/2017	0.41	13.9	0.46	0.031	3.1 %

Table S18. Volumetric Uncertainty-Submerged

Period	Survey Pair	Volumetric Uncertainty (m)	$\overline{z}_{max}$ (m)	$\overline{z}_{min}$(m)	Normalized Volumetric Uncertainty (non-dimensional)	Volumetric % Error
**Storm**	**AOI 1; 10/2012-10/2013**	0.39	-0.36	-15.0	0.027	2.7
**Storm**	**AOI 2; 10/2012-10/2013**	0.42	-0.49	-15.0	0.029	2.9
**Storm**	**AOI 3; 10/2012-10/2013**	0.40	-0.48	-14.4	0.029	2.9
**Storm**	**AOI 4; 10/2012-10/2013**	0.43	-0.44	-14.4	0.031	3.1
**Storm**	**AOI 5; 10/2012-10/2013**	0.54	-0.55	-15.6	0.036	3.6
**Storm**	**AOI 6; 10/2012-10/2013**	0.65	-0.65	-13.9	0.049	4.9
**Post-storm**	**AOI 1; 10/2013-06/2018**	0.38	-0.27	-14.3	0.027	2.7
**Post-storm**	**AOI 2; 10/2013-06/2018**	0.41	-0.40	-14.3	0.029	3.0
**Post-storm**	**AOI 3; 10/2013-06/2018**	0.40	-0.39	-13.6	0.030	3.0
**Post-storm**	**AOI 4; 10/2013-06/2018**	0.42	-0.35	-13.7	0.032	3.2
**Post-storm**	**AOI 5; 10/2013-06/2018**	0.53	-0.46	-14.9	0.037	3.7
**Post-storm**	**AOI 6; 10/2013-06/2018**	0.65	-0.57	-13.2	0.051	5.1

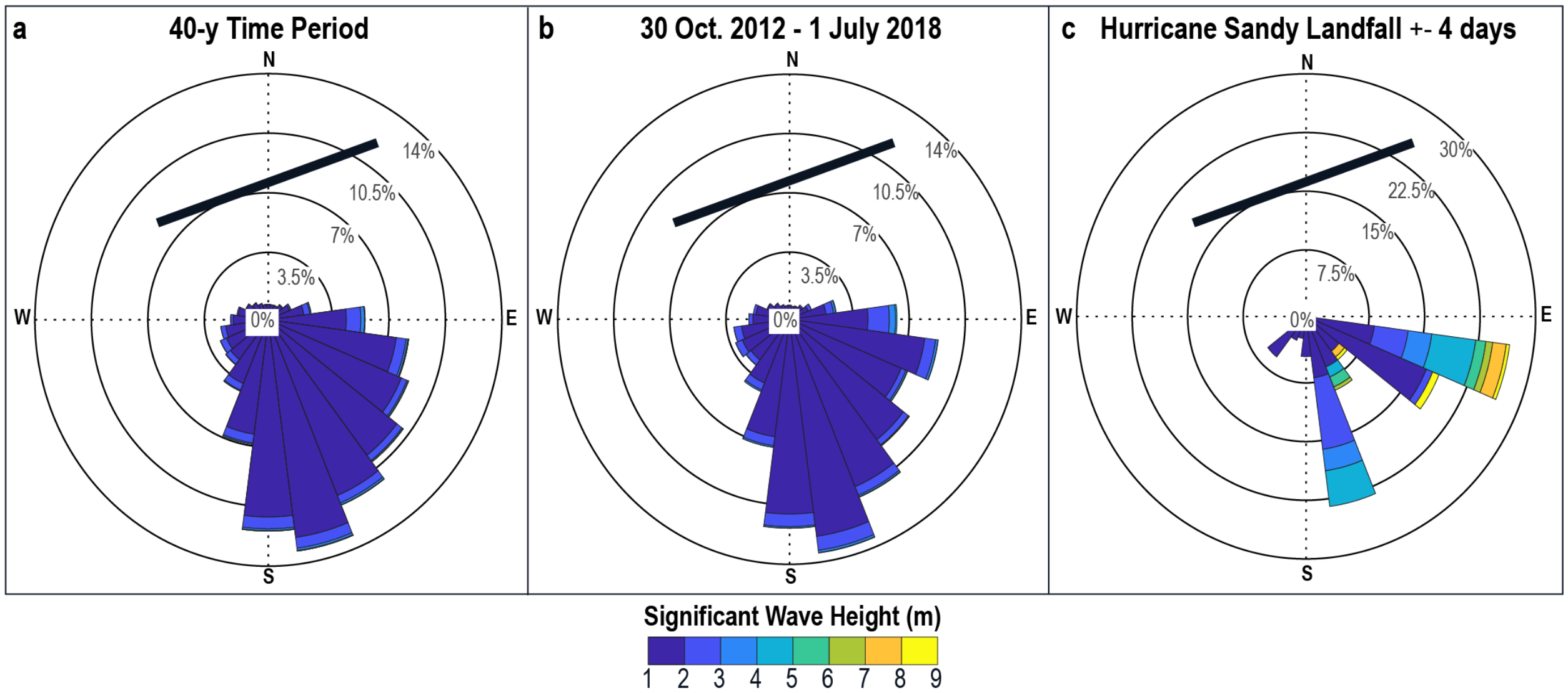

Figure S1. Wave climate comparison for different time periods. Wave characteristics generated from WIS Station 63114[1] for a) a 40-yr time period, b) the period of observation, and c) Hurricane Sandy, including four days before and after landfall. The black bar approximates barrier island orientation.

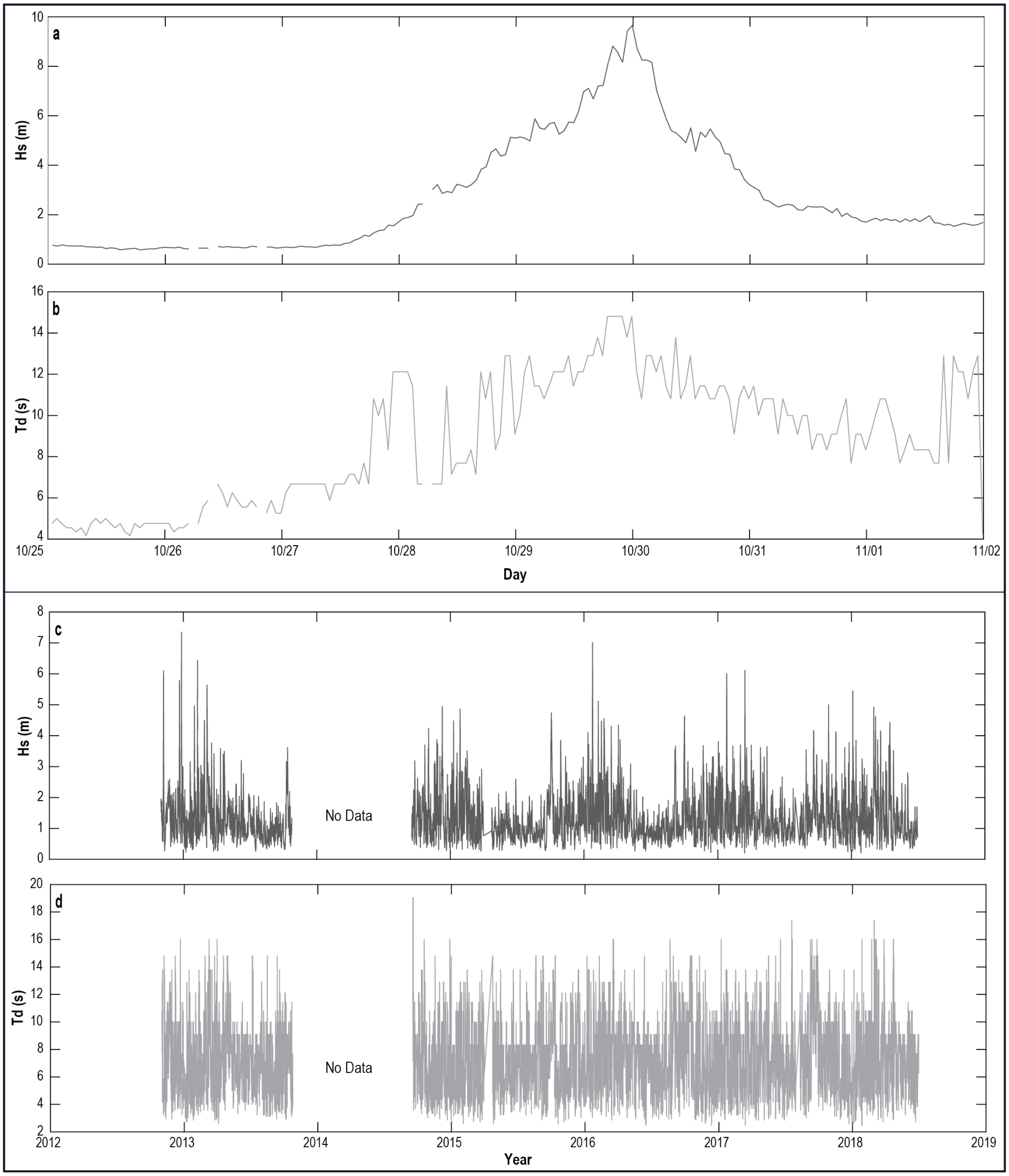

Figure S2. Time series of wave conditions. Time series of observed significant wave height (Hs; a and c) and peak periods (Td; b and d) for Hurricane Sandy, including four days before and after landfall (25 October – 4 November 2012; a and b) and the period of observation (30 October 2012 – 1 July 2018; c and d). Data are derived from National Data Buoy Center (NDBC) buoy 44025[2] and gaps represent when no wave data were available.

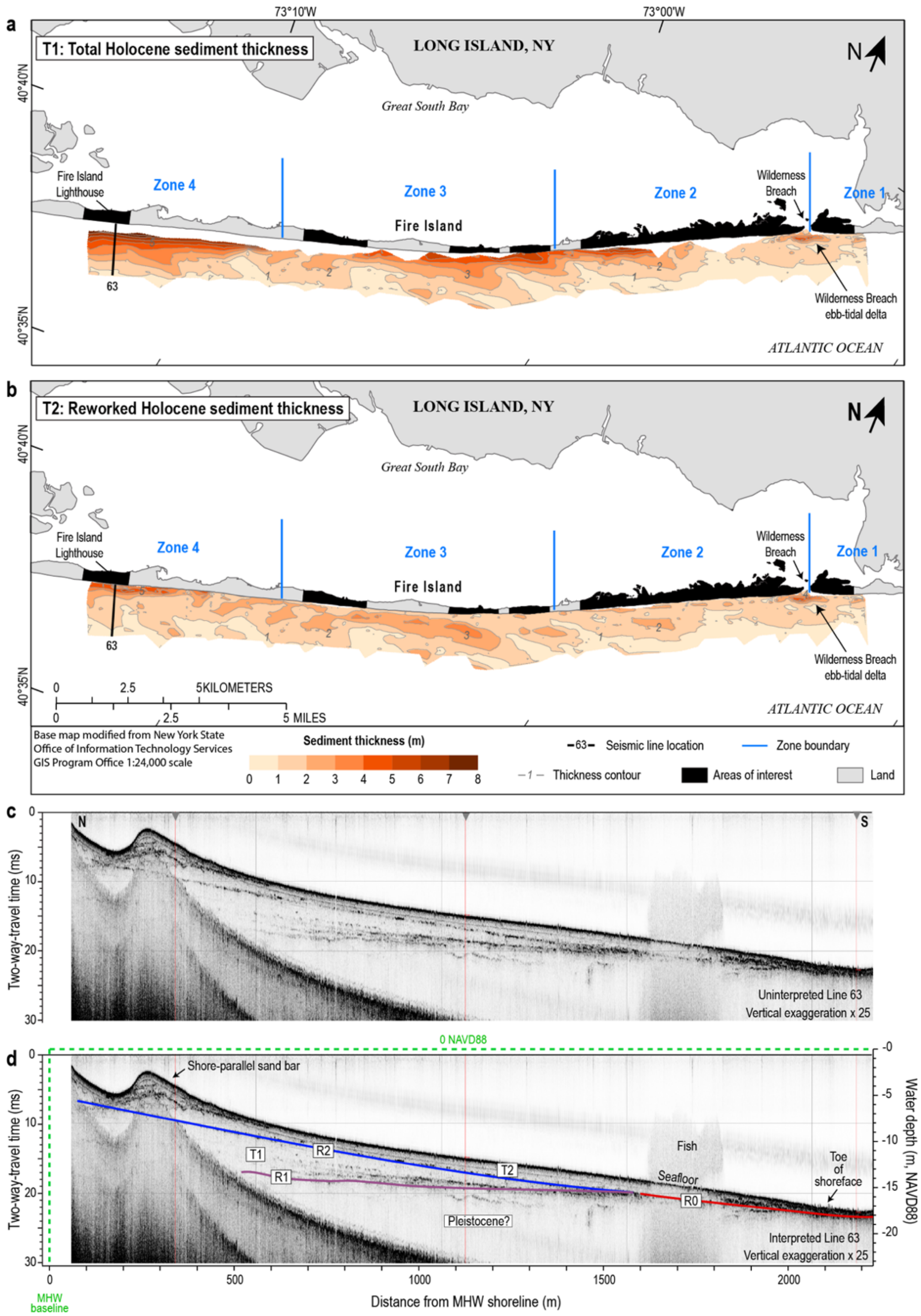

Figure S3. Variations in shoreface sediment availability along Fire Island. Modified from Locker et al., 2017[3]. Thickness of T1 or Holocene shoreface sediment (a) and T2 or reworked Holocene shoreface sediment (b). Basemap modified from New York State Office of Information Technology Services GIS Program Office (1:24,000 scale; https://data.gis.ny.gov). An uninterpreted (c) and interpreted (d) cross-shore seismic profile from the western part of the island (location shown in a and b) showing the stratigraphic relationship between the two sediment packages. R0 denotes the transgressive ravinement surface and R1 and R2 denotes the base of the Holocene and reworked Holocene sediment packages, respectively.

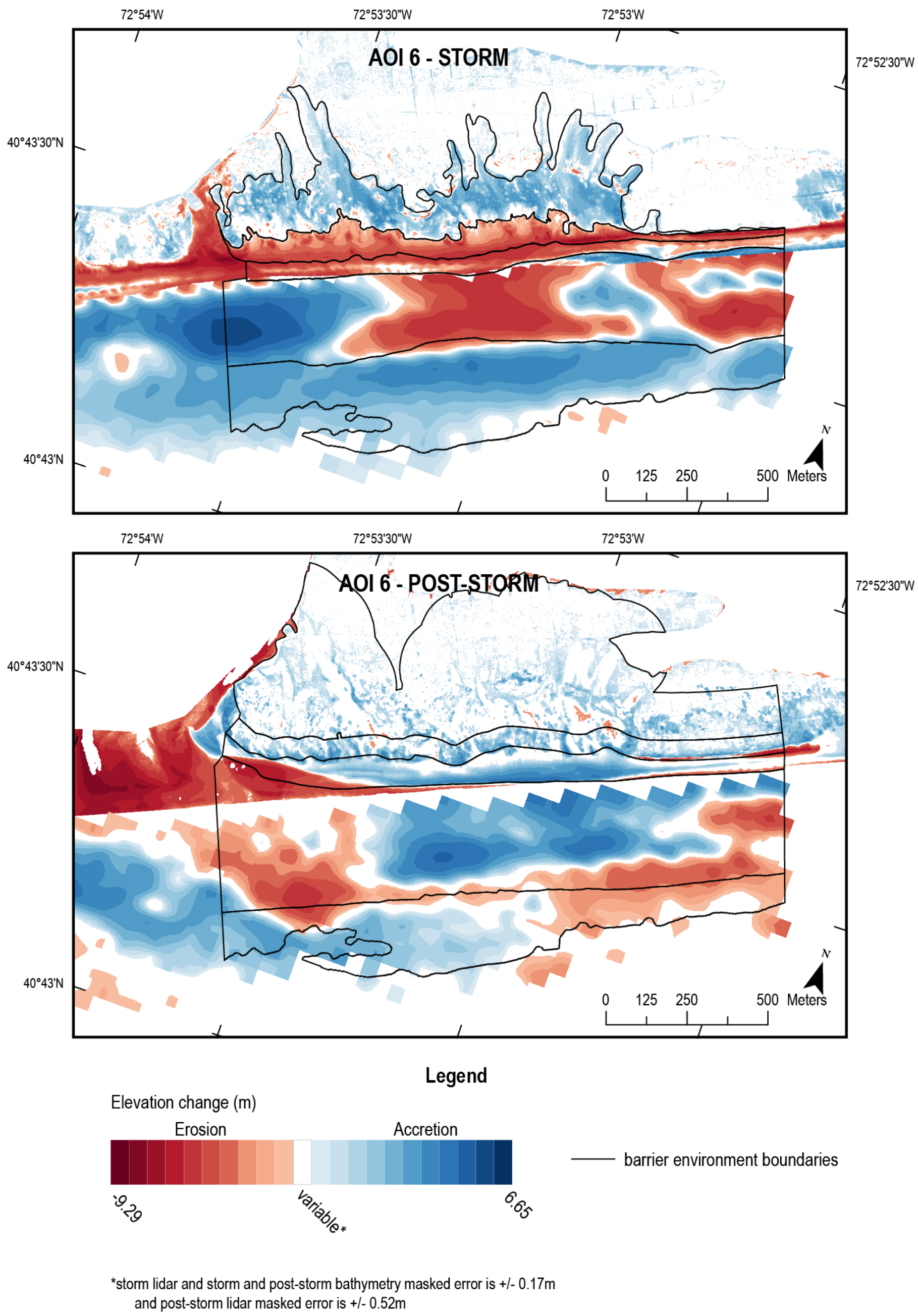

Figure S4. Storm and post-storm period barrier island elevation changes in area of interest (AOI) 6. The AOI is representative of Zone 1. Storm period topographic data from Fredericks et al., 2016[4] and Stockdon et al., 2013[5]. Storm period bathymetry data from Wright et al., 2014[6] and Wilson et al., 2017[7]. Post-storm period topographic data from Stockdon et al., 2013[5] and OCM Partners, 2025[8]. Post-storm period bathymetry data from Wilson et al., 2017[7] and Stalk et al., 2018[9].

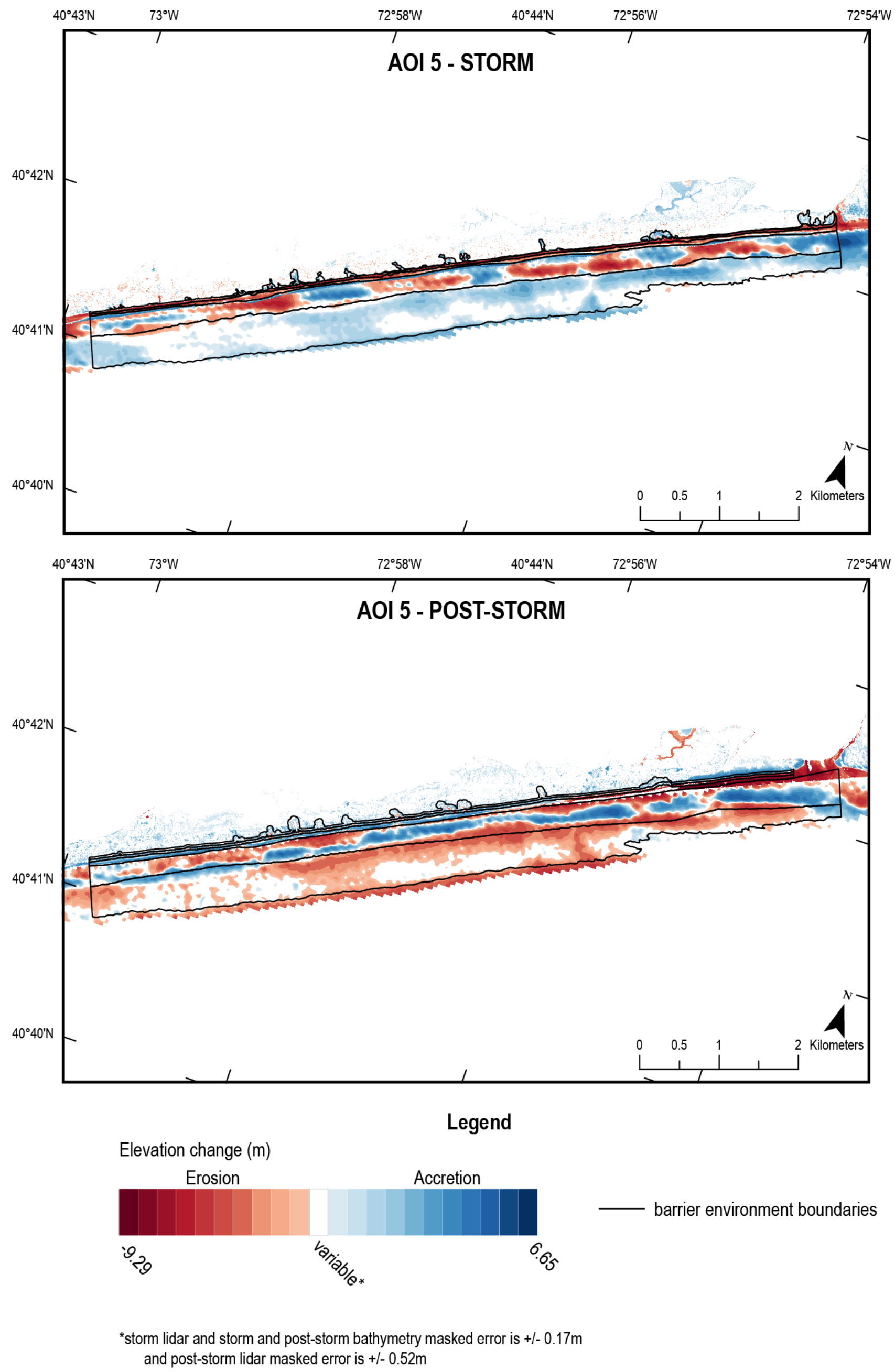

Figure S5. Storm and post-storm period barrier island elevation changes in area of interest (AOI) 5. The AOI is representative of Zone 2. Storm period topographic data from Fredericks et al., 2016[4] and Stockdon et al., 2013[5]. Storm period bathymetry data from Wright et al., 2014[6] and Wilson et al., 2017[7]. Post-storm period topographic data from Stockdon et al., 2013[5] and OCM Partners, 2025[8]. Post-storm period bathymetry data from Wilson et al., 2017[7] and Stalk et al., 2018[9].

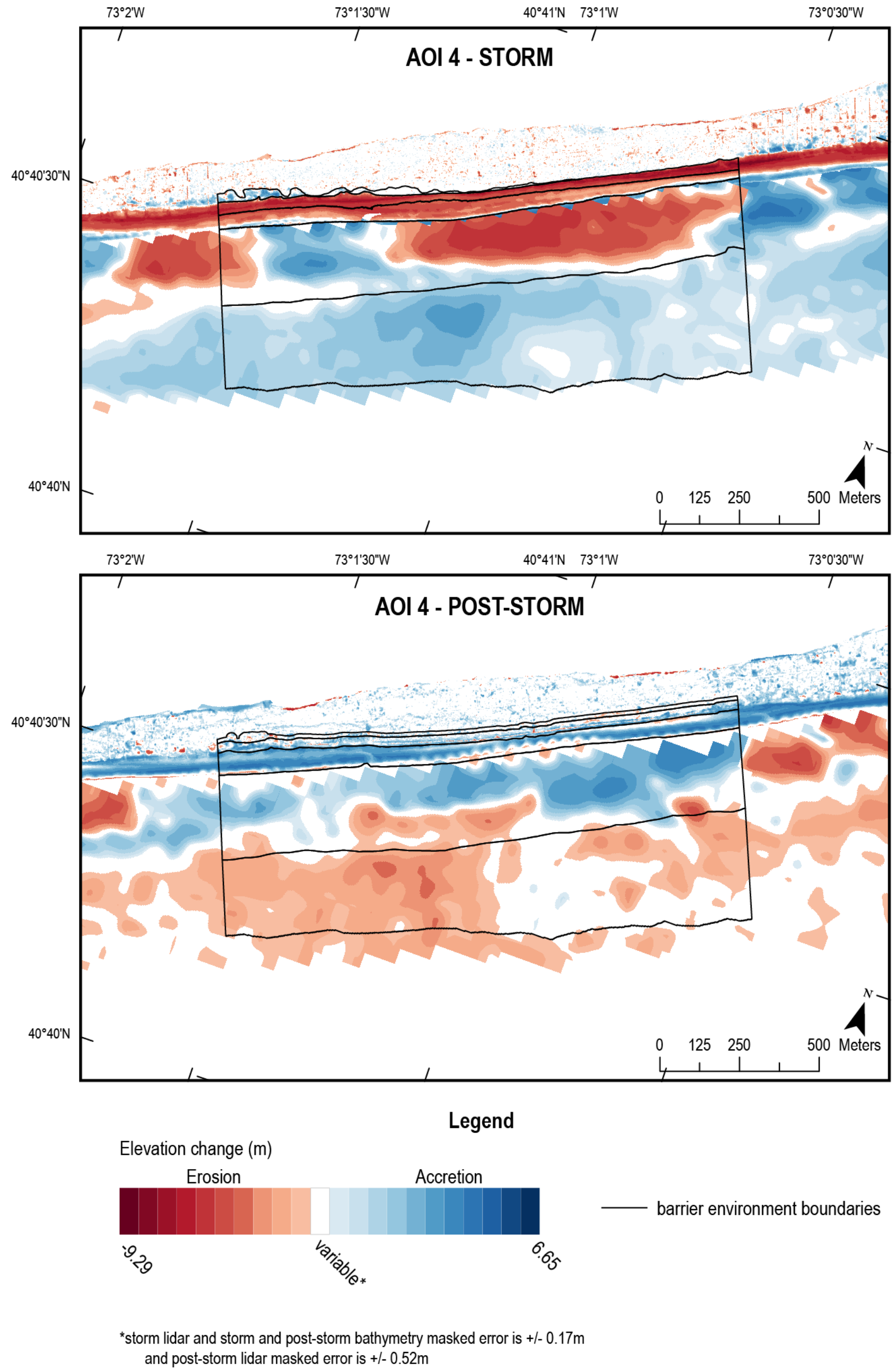

Figure S6. Storm and post-storm period barrier island elevation changes in area of interest (AOI) 4. The AOI is representative of Zone 3. Storm period topographic data from Fredericks et al., 2016[4] and Stockdon et al., 2013[5]. Storm period bathymetry data from Wright et al., 2014[6] and Wilson et al., 2017[7]. Post-storm period topographic data from Stockdon et al., 2013[5] and OCM Partners, 2025[8]. Post-storm period bathymetry data from Wilson et al., 2017[7] and Stalk et al., 2018[9].

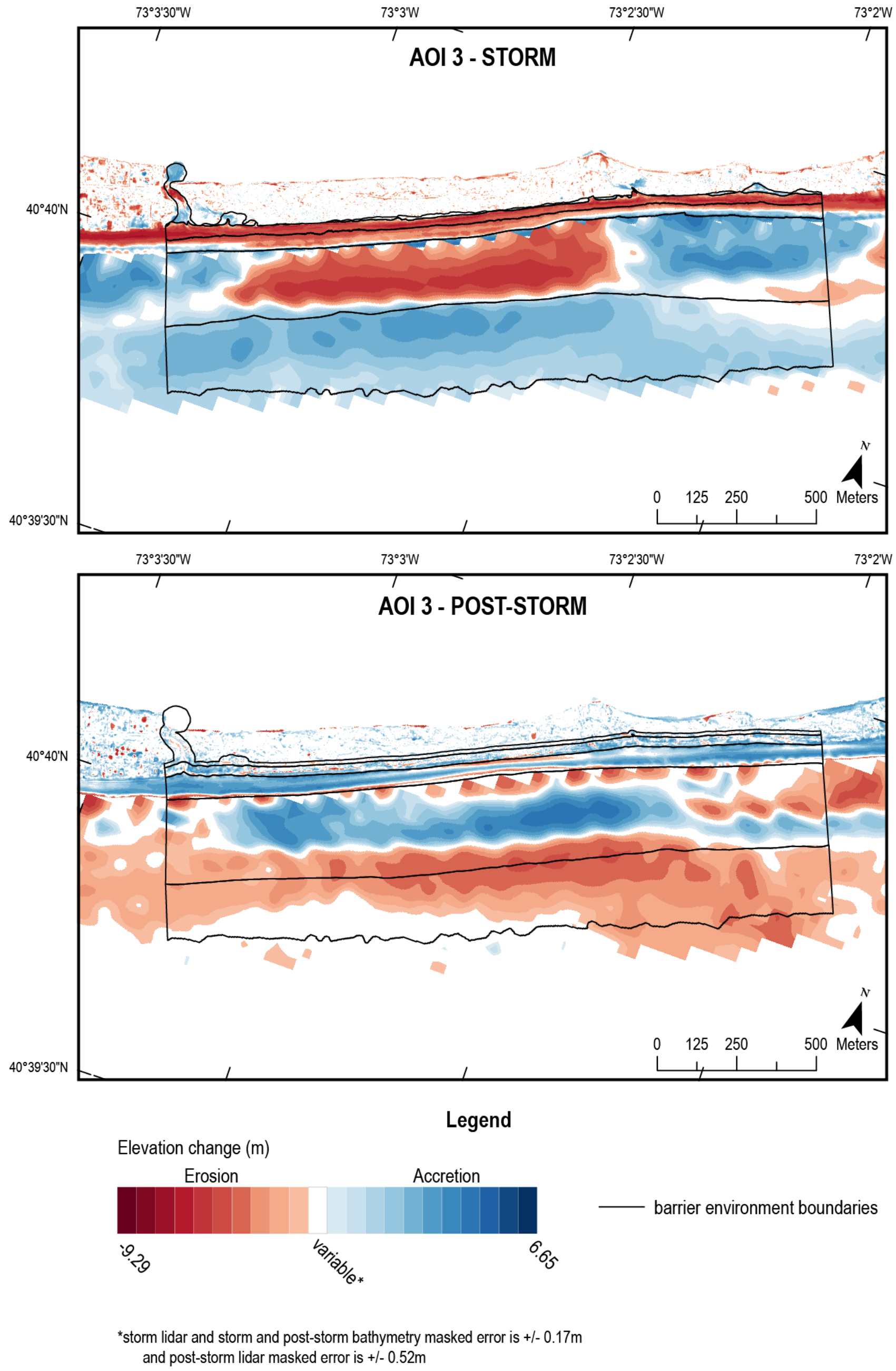

Figure S7. Storm and post-storm period barrier island elevation changes in area of interest (AOI) 3. The AOI is representative of Zone 3. Storm period topographic data from Fredericks et al., 2016[4] and Stockdon et al., 2013[5]. Storm period bathymetry data from Wright et al., 2014[6] and Wilson et al., 2017[7]. Post-storm period topographic data from Stockdon et al., 2013[5] and OCM Partners, 2025[8]. Post-storm period bathymetry data from Wilson et al., 2017[7] and Stalk et al., 2018[9].

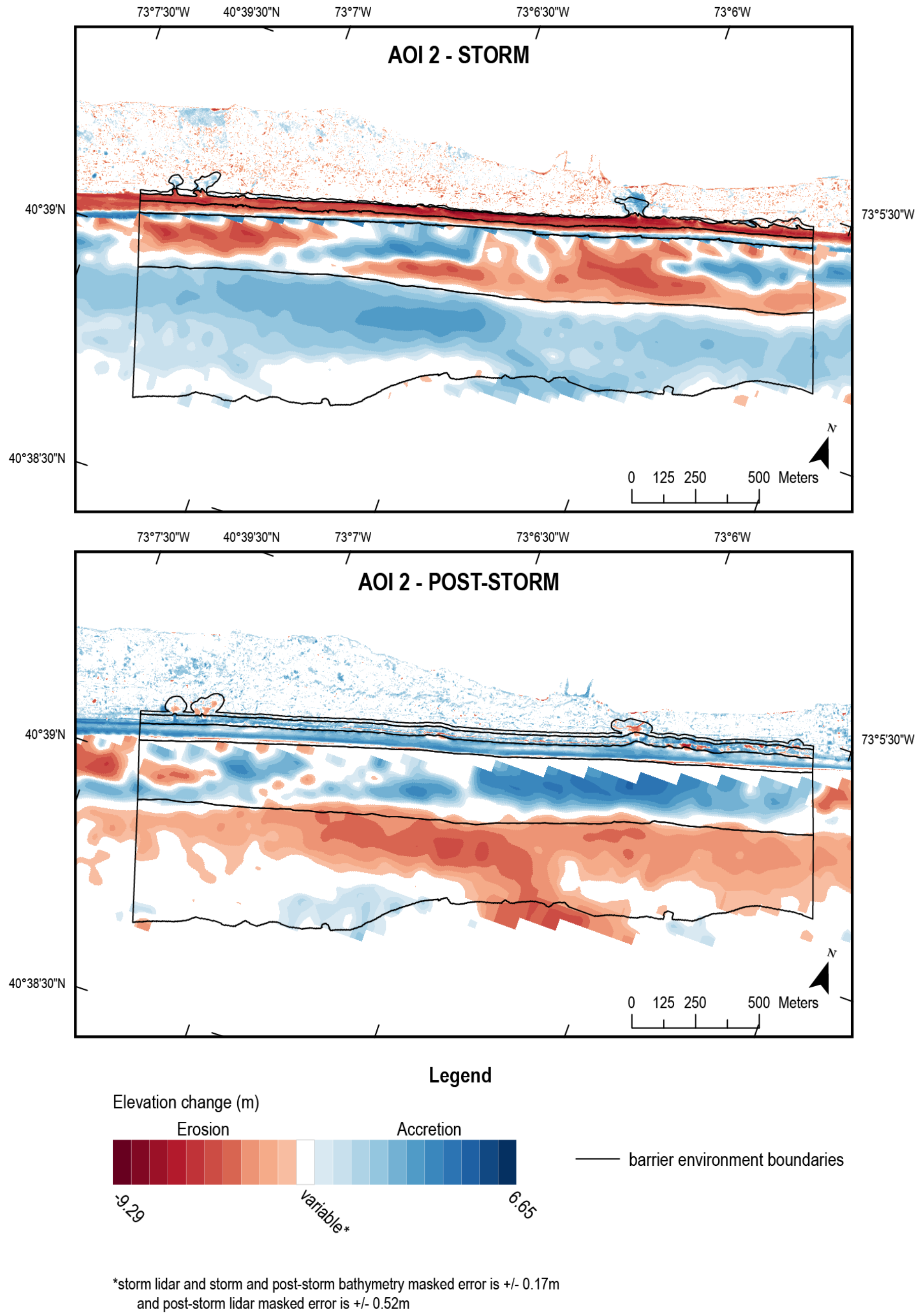

Figure S8. Storm and post-storm period barrier island elevation changes in area of interest (AOI) 2. The AOI is representative of Zone 3. Storm period topographic data from Fredericks et al., 2016[4] and Stockdon et al., 2013[5]. Storm period bathymetry data from Wright et al., 2014[6] and Wilson et al., 2017[7]. Post-storm period topographic data from Stockdon et al., 2013[5] and OCM Partners, 2025[8]. Post-storm period bathymetry data from Wilson et al., 2017[7] and Stalk et al., 2018[9].

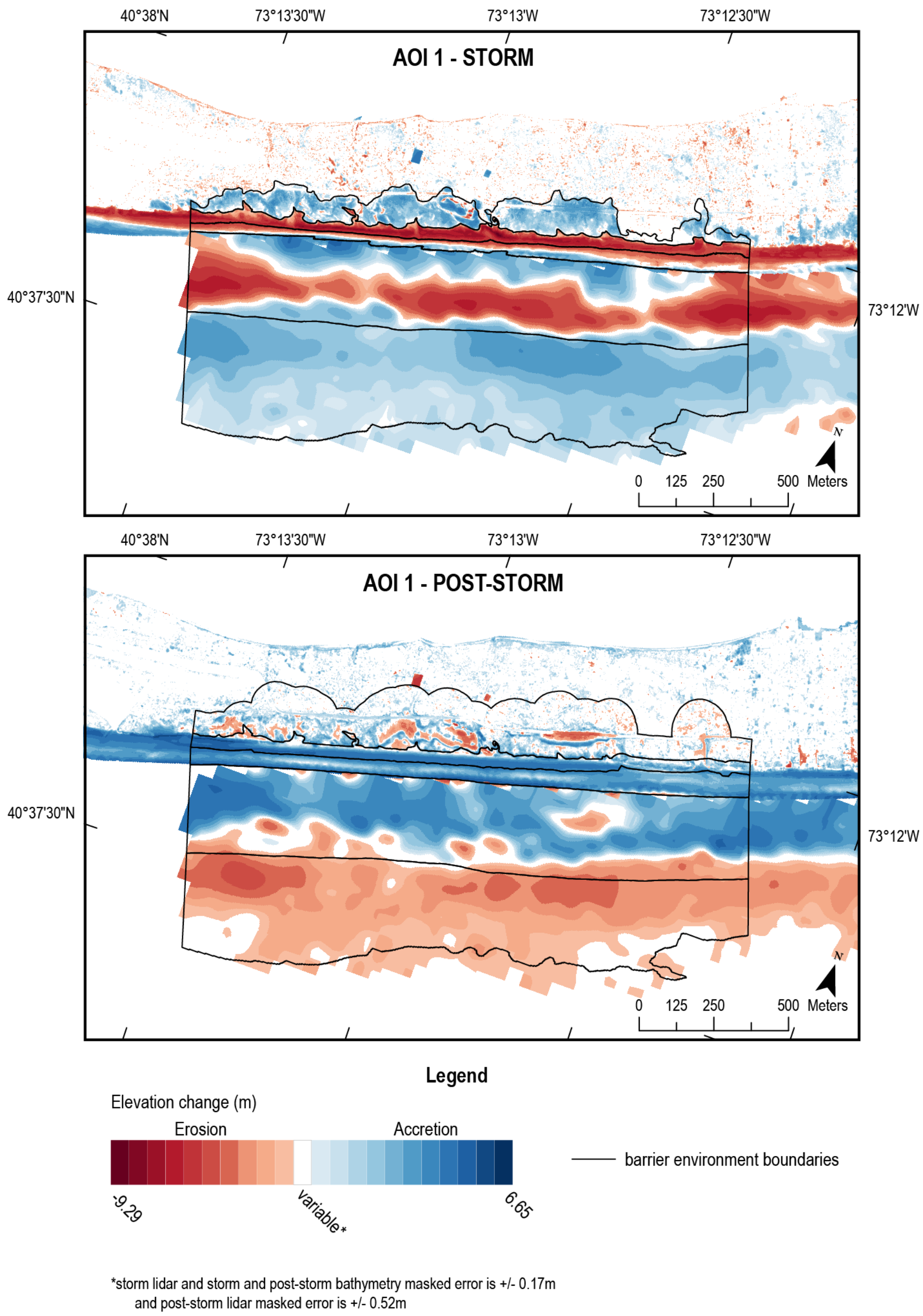

Figure S9. Storm and post-storm period barrier island elevation changes in area of interest (AOI) 1. The AOI is representative of Zone 4. Storm period topographic data from Fredericks et al., 2016[4] and Stockdon et al., 2013[5]. Storm period bathymetry data from Wright et al., 2014[6] and Wilson et al., 2017[7]. Post-storm period topographic data from Stockdon et al., 2013[5] and OCM Partners, 2025[8]. Post-storm period bathymetry data from Wilson et al., 2017[7] and Stalk et al., 2018[9].

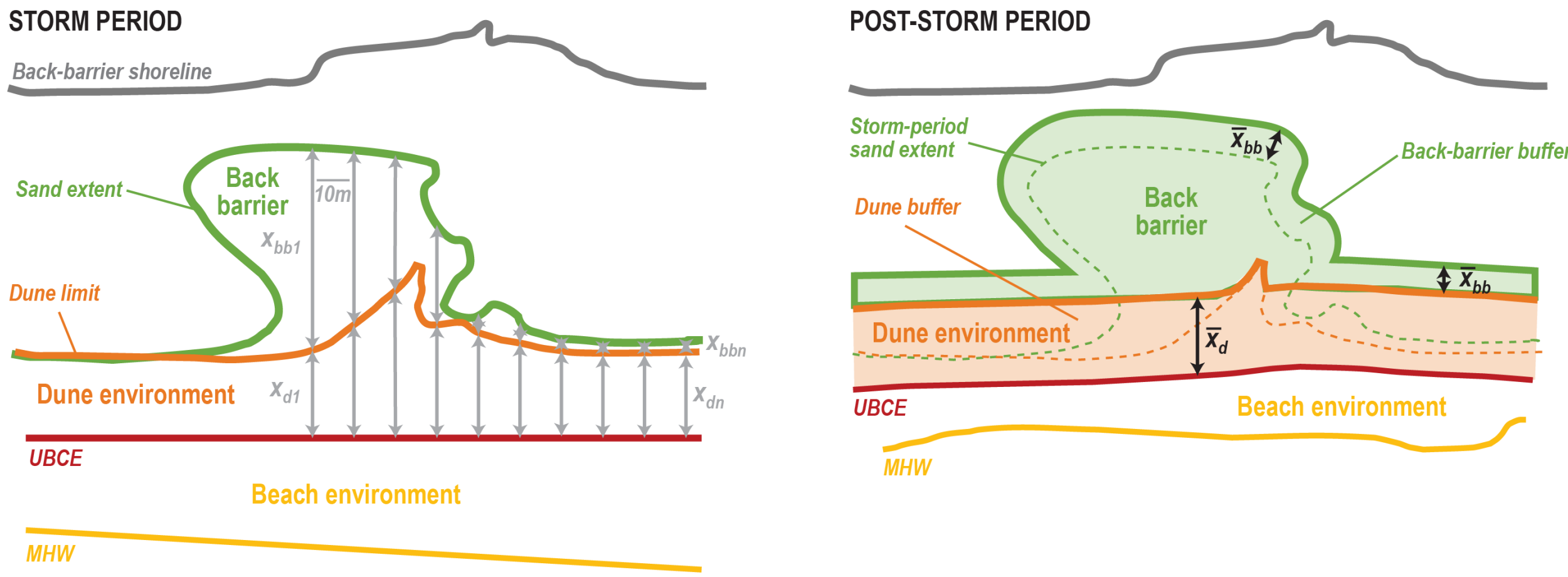

Figure S10. Schematic showing the spatial relationships between terrestrial storm and post-storm-period boundaries and the methods used to derive post-storm dune and back-barrier boundaries. Post-storm period dune limit and back-barrier boundaries were geomorphically derived. We calculated the cross-shore width of the dune (x_d) and back-barrier (x_{bb}) environments every 10m and then calculated the averages ($\bar{x}_d$ and $\bar{x}_{bb}$, respectively). We added a buffer to the post-storm beach boundary (UBCE) equal to $\bar{x}_d$ to derive the post-storm dune limit. We also added a buffer to the post-storm dune limit equal to $\bar{x}_{bb}$ to derive the post-storm period back-barrier boundary.

References

1. U.S. Army Corps of Engineers (USACE). Wave Information Study (WIS) Hindcast Data. Engineer Research and Development Center (ERDC) Coastal Hydraulics Laboratory.
2. NOAA National Data Buoy Center (NDBC). Meterological and oceanographic data collected from the National Data Buoy Center Coastal-Marine Automated Network (C-MAN) and moored (weather) buoys: Station 44025. NOAA Centers for Environmental Information.
3. Locker, S. D. *et al. Nearshore Sediment Thickness, Fire Island, New York*. 29 (2017) doi:https://doi.org/10.3133/ofr20171024.
4. Fredericks, X., Hapke, C. J. & Lentz, E. E. Coastal Topography-Fire Island, New York, 07 May 2012. https://doi.org/https://doi.org/10.5066/F7513WBW (2016).
5. Stockdon, H., Doran, K. S., Sopkin, K. L., Smith, K. E. L. & Fredericks, X. Coastal Topography-Northeast Atlantic Coast, Post-Hurricane Sandy, 2012. https://doi.org/https://doi.org/10.3133/ds765 (2013).
6. Wright, C. W. *et al.* EAARL-B Coastal Topography-Fire Island, New York, Pre-Hurricane Sandy, 2012: Seamless (Bare Earth and Submerged). (2014).
7. Wilson, K. E. *et al.* Post-Hurricane Sandy Beach Profile Survey-Fire Island Inlet to Moriches Inlet 2013. https://doi.org/https://doi.org/10.5066/F7XK8CSR (2017).
8. OCM Partners. 2017 USACE NCMP Topobathy Lidar DEM: East Coast (NY, NJ, DE, MD, VA, NC, SC, GA). NOAA National Centers for Environmental Information (2025).
9. Stalk, C. A. *et al.* Coastal Bathymetry Data Collected in June 2018 From Fire Island, New York: Wilderness Breach and Shoreface. (2018).
