# Supplementary material for: Barrier island geomorphic complexity drives storm and post-storm response
Source: Nat Commun. 2026 Sep 24;17:10172. doi: 10.1038/s41467-026-77143-6 (PMC13612595; doi:10.1038/s41467-026-77143-6)
Supplement: Supplementary file 2 [file 41467_2026_77143_MOESM2_ESM.pdf]

Corresponding author(s): *Double-anonymous peer review submissions: write DAPR and your manuscript number here instead of author names.*

Last updated by author(s): *YYYY-MM-DD*

Reporting Summary

Nature Portfolio wishes to improve the reproducibility of the work that we publish. This form provides structure for consistency and transparency in reporting. For further information on Nature Portfolio policies, see our Editorial Policies and the Editorial Policy Checklist.

Statistics

For all statistical analyses, confirm that the following items are present in the figure legend, table legend, main text, or Methods section.

n/a	Confirmed	
☒	☐	The exact sample size (*n*) for each experimental group/condition, given as a discrete number and unit of measurement
☒	☐	A statement on whether measurements were taken from distinct samples or whether the same sample was measured repeatedly
☒	☐	The statistical test(s) used AND whether they are one- or two-sided<br>*Only common tests should be described solely by name; describe more complex techniques in the Methods section.*
☒	☐	A description of all covariates tested
☒	☐	A description of any assumptions or corrections, such as tests of normality and adjustment for multiple comparisons
☒	☐	A full description of the statistical parameters including central tendency (e.g. means) or other basic estimates (e.g. regression coefficient) AND variation (e.g. standard deviation) or associated estimates of uncertainty (e.g. confidence intervals)
☒	☐	For null hypothesis testing, the test statistic (e.g. *F*, *t*, *r*) with confidence intervals, effect sizes, degrees of freedom and *P* value noted<br>*Give P values as exact values whenever suitable.*
☒	☐	For Bayesian analysis, information on the choice of priors and Markov chain Monte Carlo settings
☒	☐	For hierarchical and complex designs, identification of the appropriate level for tests and full reporting of outcomes
☒	☐	Estimates of effect sizes (e.g. Cohen's *d*, Pearson's *r*), indicating how they were calculated

Our web collection on statistics for biologists contains articles on many of the points above.

Software and code

Policy information about availability of computer code

Data collection	Details about softwares used to acquire and process the elevation data used in this study are provided in the metadata associated with the citations for each data type.
Data analysis	Details about softwares used to analyze the data used in this study are provided in the Methods. No software codes were used.

For manuscripts utilizing custom algorithms or software that are central to the research but not yet described in published literature, software must be made available to editors and reviewers. We strongly encourage code deposition in a community repository (e.g. GitHub). See the Nature Portfolio guidelines for submitting code & software for further information.

Data

Policy information about availability of data

All manuscripts must include a data availability statement. This statement should provide the following information, where applicable:

- Accession codes, unique identifiers, or web links for publicly available datasets
- A description of any restrictions on data availability
- For clinical datasets or third party data, please ensure that the statement adheres to our policy

All elevation source data are publicly available via the citations provided in the methods. All data generated from those source data and analyzed in this study are included in the supplementary information.

Research involving human participants, their data, or biological material

Policy information about studies with human participants or human data. See also policy information about sex, gender (identity/presentation), and sexual orientation and race, ethnicity and racism.

Reporting on sex and gender	N/A
Reporting on race, ethnicity, or other socially relevant groupings	N/A
Population characteristics	N/A
Recruitment	N/A
Ethics oversight	N/A

Note that full information on the approval of the study protocol must also be provided in the manuscript.

Field-specific reporting

Please select the one below that is the best fit for your research. If you are not sure, read the appropriate sections before making your selection.

☐ Life sciences ☐ Behavioural & social sciences ☒ Ecological, evolutionary & environmental sciences

For a reference copy of the document with all sections, see nature.com/documents/nr-reporting-summary-flat.pdf

Ecological, evolutionary & environmental sciences study design

All studies must disclose on these points even when the disclosure is negative.

Study description	Geomorphological analysis of elevation changes from the terrestrial and submerged components of a barrier island located in the United States. Three terrestrial elevation surveys and three submerged elevation surveys were used.
Research sample	The island was divided into 4 geomorphic zones based on a previous analysis of its morphology.
Sampling strategy	Natural areas, or those without human infrastructure and direct sediment placement, greater than 1km in alongshore length were chosen to minimize direct human interference with the elevation changes. We refer to these as areas of interest (AOIs). We had a total of 6 AOIs, with one in each of the 4 geomorphic zones previously identified.
Data collection	Terrestrial elevation data were collected using topographic lidar. Submerged elevation data were collected using topo-bathymetric lidar and acoustic, boat-based soundings. Each survey has a citation associated with it in the methods section of the manuscript.
Timing and spatial scale	Terrestrial data were collected in May 2012, November 2012, and September 2017. Submerged data were collected in October 2012, October 2013, and June 2018. All AOIs were covered by each dataset, roughly spanning ~30 km of the island.
Data exclusions	N/A
Reproducibility	When elevation data are gridded and differenced following the methodology specified in the paper, the same volumes are calculated.
Randomization	N/A
Blinding	N/A

Did the study involve field work? ☒ Yes ☐ No

Field work, collection and transport

Field conditions	We were only involved in the data collection for the June 2018 submerged elevation dataset. The fact that we have a dataset conveys that the surf zone conditions were conducive to collecting data.
Location	Fire Island, NY, USA; Fire Island National Seashore; shoreface water depths from 0-20 m.
Access & import/export	Beach access in wilderness areas was permitted by the U.S. National Park Service. Logistical support for spotting of threatened and endangered shorebirds was provided by staff from Fire Island National Seashore. No physical samples were collected during the June 2018 submerged elevation survey.
Disturbance	N/A

Reporting for specific materials, systems and methods

We require information from authors about some types of materials, experimental systems and methods used in many studies. Here, indicate whether each material, system or method listed is relevant to your study. If you are not sure if a list item applies to your research, read the appropriate section before selecting a response.

Materials & experimental systems

n/a	Involved in the study
x	☐ Antibodies
x	☐ Eukaryotic cell lines
x	☐ Palaeontology and archaeology
x	☐ Animals and other organisms
x	☐ Clinical data
x	☐ Dual use research of concern
x	☐ Plants

Methods

n/a	Involved in the study
x	☐ ChIP-seq
x	☐ Flow cytometry
x	☐ MRI-based neuroimaging

Plants

Seed stocks	N/A
Novel plant genotypes	N/A
Authentication	N/A
