# Supplementary material for: Barrier island geomorphic complexity drives storm and post-storm response
Source: Nat Commun. 2026 Sep 24;17:10172. doi: 10.1038/s41467-026-77143-6 (PMC13612595; doi:10.1038/s41467-026-77143-6)
Supplement: Supplementary file 3 [file 41467_2026_77143_MOESM3_ESM.pdf]

nature portfolio

Peer Review File

Barrier island geomorphic complexity drives storm and post-storm response

Corresponding Author: Dr Jennifer Miselis

This file contains all reviewer reports in order by version, followed by all author rebuttals in order by version.

Version 0:

Reviewer comments:

Reviewer #1

(Remarks to the Author)
This manuscript presents the results of a monitoring program that holistically addresses the response of a barrier island to the impact of a major storm as well as its recovery, although partially. For that, the authors integrate observations on the vertical changes through all barrier compartments from the backbarrier to the outer shoreface. These type of analysis, yet extremely relevant and precious, remain uncommon given the difficulties of mapping shoreface areas or even an entire island, which are in turn key to fully understand barrier dynamics and possible responses. Therefore, the authors successfully quantified magnitudes and directions of storm-driven sediment fluxes across barrier compartments and estimated rates of recovery.

The present work discriminated the magnitude of changes pre- and post-storm across the different compartments or environments within the barrier, informing about the relative importance of each one of those to the response of the barrier to the analysed storm. Through this approach, the authors were able to conclude that washover was not as high as expected. Yet, this result may raise some doubts and could probably be looked into more detail to better understand the differences between the areas with overwash and the areas without, along the zone that most likely register this process, and the characteristics of the waves impacting during the storm to better understand why is this result so different from expected. In addition, if so different, would that mean that the authors were not expecting so much transference of sand seaward?

Another very relevant result relates to the fluxes and trajectories of sediment during the recovery. These results highlight the importance of large scale monitoring through the morphological continuous in order to actually understand how these systems are coping with impacts and adapting to ongoing change conditions. The accepted fact that sediment did not stay within the mapped region is also highly relevant and points to complex recovery processes, limiting our ability to understand/predict this process. In fact, these results stress our limitations to fully understanding sediment exchange across the barrier profile and lateral gradients. In this line, the present work recognises its limitations to fully map or understand sediment trajectories, especially during the recovery phase because of the spatial restrictions.

The authors also mention that they also aim to identify modes of recovery to contribute to better understand island trajectories. However, I believe this has not been addressed by the authors, or it remains obscured because of the way the authors decided to present their results, in fact, there is a lack of discussion about processes or explanations about the observed changes, so I cannot see these modes of recovery in the manuscript in its present shape. Instead, there is a clear effort to focus on the data and avoid speculations.

The present work introduces very relevant results on the impact and subsequent recovery of a barrier island to the impact of Hurricane Sandy in 2012. The relevance of the present work lays on the fact that the authors estimated sediment budgets during the erosion and recovery phases based on a series of topobathymetric surveys that extend the observations five years after the impact of the storm. The results confirm and demonstrate that recovery may be a very slow process, but also leave the possibility that total recovery (in terms of sediment budgets) may not be reached, given the losses of sediment to unknown regions. Therefore, I find this work of high relevance giving the current situation of coastal vulnerability and the lack of fully understanding of how these highly complex processes actually work. This is extremely relevant and highlight the importance of extensive monitoring programs, not very common, but key to actually understanding system response at a large temporal and spatial scale. The work targets knowledge gaps related to data scarcity, namely data integrating changes across and along the morphological continuous.

Overall I believe this is a very important contribution as it addresses a very important problem, not so well understood and proves the importance of this type of work, particularly, it demonstrate the value of monitoring programs and sediment budget estimates to actually understand the response of such complex settings.

This type of work is rather uncommon because of the scarcity of this type of data, through space and time, focusing on the impact and recovery after a storm. Most examples looking into the impact of storms at the scale of an island use to focus on the impact or erosive phase, because of the related risks and loss of geomorphologic capital. Rare are the works monitoring the whole process and in fact, as demonstrated in this work, recovery may be such a long process and has not raised the same attention despite of being equally important. For instance, a work that could compare, because of the type of data and coverage of the cross-shore profile, can be found in Ruiz de Alegria et al. (2025). The authors also focus on understanding exchange between different profile/barrier compartments, extending to the shoreface. Some differences are worth noting between both works that further highlight the relevance of the present work, these are the spatial scale (an attempt to close a morphological continuous was done in the present work), the type of coast, which the former is located in an embayment, the present is located in an open coast, which turns any attempt of sediment budget estimates, far more complicated. Yet, both works find that the outer/offshore limit of their AOI may not be capable to capturing possible exchanges with deeper areas, a pinpointed knowledge gap.

The results do support the conclusions, although they could be presented/discussed in an even simpler way, specially focusing on explaining the implications of the morphological state to the response of the zone to the storm and recovery. The authors claim that the morphological state of each zone of the island is key on the type of response observed. However, the way the results are presented does not shows clearly how the state roles the response and additionally, I see this as a kind of chicken-egg problem. A morphological state is achieved because of a dominant sediment flux and resulting budgets that result from the interaction between the sediment supply and hydrodynamics, so, to which extent the results are only reinforcing a state that results from a lack (excess) of sediment rather than the state controlling that lack (excess) of sediment itself or the fluxes of sediment? Said that, I would like to reinforce the fact that the results are a bit confusing and long, without clear outcomes. The only thing that is clear is that the transgressive zone favours the inland transfer of sand, through overwash and inlet breaching/maintenance, but the relation with the outer shoreface, key to the whole problem, remains unclear. Also, the elongation zone seems to gain sediments faster, which is expected given that it is at the end of the littoral drift. But, what about the progradational zones? How do they respond? It would help if the major behaviours of response (modes) are associated with a morphological type in a simplified way, this could help understand the conclusions of the authors. Another aspect that remains a bit confused is the balance of sediment. From what I understand, a very large part leaves the region, however, given that the authors only calculate the changes for the AOI, could there be a possibility that part of the sediment could be in the areas not calculated but still within the island region?
One more aspect that is not considered by the authors, though estimated, are the errors. Some of the changes seem to fall within the error range but this is not mentioned across the main sections of the manuscript. I believe this is highly important and could help to understand better the limitations of such work, but also, some of the missing volumes?

It is not easy to understand or find how much sediment arriving at the outer shoreface stayed there or leaved from the volume transferred during the impact of the storm. This could be improved so the results can be followed in an easier, more direct way. I find that it seems difficult to generalise responses, but maybe the responses are in general not so different? The main difference is the recovery, I would put emphasis to this and the balances, and the fact that zones with high dunes cannot have overwash, but how this means they are less resilient or that show less resilient responses? Are the zones with high dunes eroding (losing sediments) more than the others? If barriers have wider zones with high dunes, it means they have not yet reached the transgressive state, and this also means that they may eventually reach it, if sediment recovery does not work. Now, if nourishment prevents this, the shift of state is delayed and then, the question is, as the authors also mention, until when humans will be able to hold the line.

Finally, I believe that the authors could give more information regarding the offshore limit or boundary of their analysis domain as this seems to be a possible limitation to the closure of the budgets. In this line, I would appreciate if the authors could explain better how they obtain the estimated DoC they use to delineate the seaward limit of the inner shoreface, or to justify that the offshore boundary of their domain falls within the possible extent of sediment movement. After checking some of the results for this island using WIS stations, the annual averages are far larger than the ones suggested by the authors in the present work, but what is more important, given that Sandy was a storm with a very large return period and quite uncommon, I would expect a deeper DoC, this needs further justification/explanation.

In this line, it is also worth noting the net import that the authors document during the storm, however without a clear clue of the origin of the sand. Did the authors consider that given the relatively small proportion related to the error (6% compared to circa 5% of the error)? To which extent, the fact of not including the whole island could help to explain this mismatch? Yet, this is a very interesting result, once certain that is not obscured by errors, as it would imply that the impact of the storm brought sand to the studied AOI. Not necessarily what one would expect.
The methods are ok, yet, I would appreciate a bit more development of the limitations of the shoreface survey given the boundary limit and the spatial resolution. How certain the authors can be of their results across the shoreface given that the resolution is 50m, is this enough?
A bit of more detail could also be added regarding the approach to estimate the volume changes, namely in the case of the overwash given the presence of channels and bushy vegetation.
It would be also interesting and key to the conclusions, to understand to which extent the results from the few AOI, which cover a very small proportion of the whole island, are representative of the overall changes.

As mentioned earlier, the estimates to the DoC could be better explained given the importance of this parameter to the understanding of the cross-shore sediment exchanges and the very different estimates they get for their study period compared to estimates available online (Atlantic_DOC_yearly_0116.xlsx retrieved from the U.S. Army Corps of Engineers).

Smaller details:
When deciding the best approach to define the compartments, a recent paper could be also useful for the authors, see https://doi.org/10.1016/j.geomorph.2025.109610 from Bauer et al., 2025.
In figure 2.A, some of the % of deposition or erosion do not add 100.
In figure 3.C, does the net change within the shoreface include the outer shoreface?
The authors tried in some cases to generalise some patterns, like in line 145: "Those percentages decreased downdrift in Zones 2-4…", however, this is not what the results indicate, with Z4 being higher than 2 and3 and very close to 1.
There is still one more question that the work raises and the relates to the period of analysis, I imagine that new storms impacted the region over the five years of analysis, sending backward the recovery trend, was this the case? if so, this should be stated and the implications discussed.
It would be very helpful is the outcomes on response patterns are attributed to each morphological state in a simple way, in its present shape, the reader cannot easily see the implications of the state to the response patterns.
I believe that it would be interesting if the authors also consider the possibility of the sediment not returning to the system. Also, the estimates are interesting but based on a linear response, how certain are the authors that the recovery will continue as for the first five years? Again, information regarding possible storms during the "recovery" or post-storm might be worth information to support the low rates of recovery, or not, but valuable information.
The authors mention in Line 319 that Z1 recorded the greatest flux from the beach. I think this should be more carefully explained as this is relative to the % eroded from the rest of compartments, but it does not necessarily mean that the volumes were larger, is this correct? So, the way it is presented it may appear that the beach here provided more sand, but this is not that simple, correct? Also because the larger proportion of eroded sand did not come from the beach but inner shoreface.
Lines 326 to 330 regarding fluxes across Z4. Is the statement correct? Not so easy to see in the figure, and when talking about shoreface, you mean inner one?
Lines 336 to 338, the authors affirm that the results highlight how the shoreface sediment availability may influence sediment fluxes, however I think this could be further discussed/explained.
Line 352, the authors mention that rapidly building dunes may impede transgressive processes. This is a bit confusing, in the first place, if a dune grows rapidly is because it is in a prograding state, in this case, I cannot see the need to become transgressive. On the other hand, if the system is close to a shift of state, I cannot easily imagine a dune building rapidly, what do the authors actually try to state here?
Line 345, the authors mention that alongshore and shoreface sediment supplies are maximised. If so, to which extent is a state control rather than gradients of longshore sediment transport controlling the patterns of barrier response, and even the long-term barrier state?
The authors mention that during the post-storm period, only 33% of the sediment from the shoreface resulted in sedimentation in inner barrier compartments, where is the remaining 67%, still in the shoreface? How much remained?

Reviewer #2

(Remarks to the Author)

Review of 'Barrier Island Geomorphic Complexity…'
By Miselis and others
Reviewed by Brad Murray

First, I enjoyed reading this manuscript, and I thank the authors for doing this valuable work. I think this work should be published, and to increase the paper's impact, I suggest two ways to improve the framing and presentation of the work.

The most important structural change in the way the work is described, it seems to me, involves the conceptual assumptions that seem to underlie the way the analyses are presented. As a brief recap, the Authors have conducted extensive (and valuable) observations in Areas of Interest (AOI) arrayed along the Long Island coastline. These AOI are positioned within zones that differ from each other in their geomorphic characteristics. In an ideal world, the AOIs would be as large as the zones, so that the two terms would be synonymous, although anthropogenic influences and, I suspect, finite resources for the observational work restrict the observations to the AOIs that are subsets of the zones. In the present manuscript, much of the results and discussion implicitly equates the AOIs to the zones, seeming to assume that changes in the AOIs are representative of their respective zones. For example, if the AOI in one zone looses volume during a time period (from one or more of the environments—beach, dunes, etc.) it seems to be assumed that the result would apply to the whole zone (if observations were able to cover the whole zone). It might be true that the findings in each of the AOIs are representative of the whole zone they are meant to represent, but we as readers don't know if that is the case. This ambiguity raises questions about interpretations that are implicitly based on the assumption that observations in the AOIs can be extrapolated to their respective zones. (The sentence beginning on line 156 provides an example: "Every other zone experienced net import, or sediment transport from outside the AOIs, the highest of which occurred in Zone 1.") I can imagine that sediment lost/gained within an AOI could be sequestered/sourced elsewhere within the zone the AOI is mean to represent (e.g. through

alongshore-heterogeneous patterns of shoreline change caused by subtle curvatures, although this is just one possible mechanism). The assumption that observations in AOIs can be extrapolated to the larger Zones should be made explicit, at least, and the bases for that assumption should be clearly described, early in the paper.

I also suggest that the Authors rewrite the paper to highlight the findings that suggest new insights about barrier processes, and reduce relatively vague references to the complexity of barrier responses to storms. For example, I think the paragraph starting on line 359 (about 'surprisingly' small overwash fluxes during a storm with characteristics that would be expected to produce a lot of overwash) describes novel and important findings that could be emphasized by paring down the other statements about what the observations apply. Similarly, the content of the paragraph starting on line 376, about the importance of inlets, the opening of inlets, and interactions with human management practices, would stand out more if the implications were pared down to the clearly novel aspects of the work.

And as an example of a relatively vague statement that could refer to aspects of barrier response to storms that are already understood, the sentence starting on line 157 states: "These results demonstrate that barrier geomorphology influences the magnitude and proportion of volume changes and the balance between cross-shore and alongshore sediment transport processes during storms." I think that statement is true, but it might hinder the paper's effectiveness, because some readers might get stuck wondering what these results suggest in concrete terms, and whether that is different than what is previously understood. For example, statements like this might lead a reader to picture the well-understood dynamic that where dunes are low enough for a storm to produce overwash, cross-shore fluxes are high (and the ration of cross-shore/alongshore transport is high), compared to location where dunes are high and cross-shore flux could be limited to seaward transport to the lower shoreface. Highlighting the most novel contributions will make the paper easier to read and more effective.

The paragraph starting on line 433 is misleading and should be removed. This paragraph makes the point that, compared to what is represented in models of long-term barrier dynamics, the observations indicate a more complicated pattern of sediment fluxes, including both onshore and offshore fluxes. I don't think the observations are relevant to barrier-dynamics models in this way, because the models represent long term sediment fluxes that are meant to average over the timescales of individual storms and post-storm recovery. It is known that the storm/recovery cycle involves alternations of offshore and onshore flux on the shoreface, but the longer-term models don't explicitly represent changes on those timescales. Complicated patterns of flux, including offshore flux during storms, is consistent with the supposition that the net long-term fluxes are all landward. It might not be true that patterns of long term flux are as simple and uniformly onshore as is assumed in models (it almost certainly isn't that simple in some natural systems), but observations over the timescales involved in this study should not be compared to long-term modeled fluxes in the way the paragraph in question does.

Minor comments and suggested edits:
Line 14: "…barrier island trajectories, yet attempts…' (add a comma)
Line 34 and 44: Make the references for 'First' and 'Second' clearer. Perhaps the last sentence of the first paragraph (line 32) could end with '…timescales are changing, for two reasons.'
Line 73: Rework the sentence starting with 'Recent work demonstrates…' to make clear that you are referring to sediment fluxes from the subaerial barrier to the shoreface via 'outwash' from the backbarrier to the front.
Line 111: Although I am not offering specific ways to accomplish this, it seemed to me when I reached the end of the Introduction that that section could benefit from some tightening. Perhaps you could look at that section to see if any reorganization or condensing could improve it?
Line 119: '…is comprised of…'
Line 137: RE 'Accumulation …exceeded losses… by about 6%.': Are you confident that the observations are precise enough to justify this interpretation, or should a caveat be added to point out that the difference could be within the error (based on Figure 2)?
Line178: RE "In Zone 1, the growth of the ebb tidal delta reduced downdrift losses …", please elaborate; what mechanisms envisioned?
Line 190: RE "… and it's position near the littoral end point of the system."—please elaborate about what mechanisms are envisioned.
Line 191: Clarify 'was distinct)
Line 194: RE 'This suggests…', please elaborate on what you mean by 'geomorphic history'. (I can guess, but I don't know if I would be right.)
Line 210: Please elaborate on '…naturally cascading sediment transport'.
Line 295: RE '…volumes of sediment exported from … Zone 2…': Couldn't this also be 'not imported into'? I think all statements like this should be rephrased in terms of net fluxes (rather than explicitly as imports or exports).
Line 308: Change to '…barrier-system scale…'
Line 332-334: The AOI isn't at the end of the system (even if the zone is)—an example of how Zones and AOIs need to be disentangled in the interpretations.
Line 336-338: RE '…these overall findings highling how shoreface sediment availability may influence the magnitude of sediment fluxes…': Please elaborate. What do you mean by 'shoreface sediment availability? Is there a limited veneer of mobile sediment on the shoreface? Or does this refer to limited rates of onshore flux from the lower shoreface?
Line 344: Please clarify 'alongshore cascades in sediment transport and morphology'
Line 371: Please elaborate '…where dune and sediment supplies are low'
Line 396: specify '…the inlet-induced geomorphic state change in Zone 2' (i.e. erosion of dunes)
Line 401-402: Reword '.. a commitment of the barrier to retreat…' which anthropomorphizes barrier dynamics

Reviewer #3

(Remarks to the Author)
Dear authors,

(General comments)
The manuscript 'Barrier island geomorphic complexity drives storm and post-storm response' by Miselis et al. deals with the impact of storms on a barrier system in New York. The paper is well structured and well written, and the topic is very relevant for those who must manage our shorelines.

The novelty of this paper is certainly the inclusion of the shoreface in the volumetric analysis. In their case, this means that they now have better ideas of the total budgets in four specific areas in Long Island, New York.

One of their main conclusions is that the amount of landward transported sediment towards the hinterland is far less than expected from previous studies. This has of course a major impact on the management of the shores on longer time scales (decades). They stated their conclusions at the end of the discussion and in the abstract, but not explicitly in a paragraph with conclusions.

Overall, the manuscript is of good quality. The introduction deals with many aspects related to the topic and the introduction is well embedded in state-of-the-art literature. For clarity to all readers, I would suggest including Figure S1 in the main manuscript. For non-coastal scientists, it immediately explains all terminology used. The discussion is also nice and includes many good points!

After reading the whole paper, there are only three aspects that I miss in this manuscript which have relevance for the study.

The first aspect is the question of what you expect to happen during a storm: overwash or breaching of the barrier. There are barrier islands where breaching is often occurring and where the associated landward fluxes of sediment during the storm are mainly governed by that. Nienhuis et al. 2021 (https://doi.org/10.1029/2021JF006066) give a first attempt to see if your barrier is vulnerable for that.

The second aspect is the influence of dune development on wash-over fans in the post-storm recovery phase. After the initial formation of a wash-over fan, you quite often see dune development on these sand bodies in the period after the storm and this is often with sediment coming from the adjacent beaches. The volumes are significant and can build up a dune area within decades and changes the sensitivity for erosion of coastal stretches. This is for instance written in https://doi.org/10.1002/esp.4610.

The third aspect is the influence of the longshore wave-driven transport on the sediment fluxes, especially during the post-storm phases. The whole manuscript is written from a cross-shore perspective, and I lack the impact of the wave-driven longshore transport on morphologic behavior. The authors do not fully neglect the aspect (there is also a longshore transport arrow in Figure 1 (is it the net wave driven longshore sediment transport vector based on the wave climate?), but they do not quantify it in relation to cross-shore fluxes. Especially in the part where the authors describe the volumetric changes between the 4 distinct areas, I would expect more reflection on that.

Sometimes, I miss some quantification of the hydrodynamic conditions. For instance, the tidal range in the area (looking to Figure 1, it is probably a wave dominated shoreline), the wave climate of the area (will probably immediately point at the net wave driven longshore transport), the maximum wave height, wave period, direction and duration of the storm, etc. The authors presented the storm characteristics, but I prefer the hydrodynamics of the storm to have an idea of the energy coming towards the shore. As a non-local reviewer, I miss the characterization of the storm and general conditions.

(Specific comments)
What do you mean by 'leading to non-uniform adaptation to forcing changes' on page 2?
What do you explicitly mean by 'surprising ways' on page 2?
Can you quantify 'extreme water levels' on page 4?
When did the storm breach occur (you talk about the storm breach Fire Island on page 5)?
You introduced 'inner and outer shoreface' on page 5. Later, reading the methods, I got an idea of the values of the boundaries. Why did you use a boundary based on hydrodynamics (wave climate) and not on morphology (nearshore zone with bars is inner zone, outer shoreface is lower zone). Could you not work with (apply) that general definition?
On page 5, you try to qualitatively say something about the small magnitude of observed net import. A quantification would make this even better.
On page 7, I always ask myself what the normal longshore drift is. You also talk about 'updrift sediment supply' without mentioning the amount, and later on page 8 about 'associated trapping of updrift sediment'. Again, can you quantify the relative importance of longshore transport in relation to cross-shore components?
On page 10, you talk about 10 times less high post-storm sediment fluxes. This is probably valid for most of the fluxes. However, the aeolian fluxes will probably be much higher in the post-storm period?
On page 10, you talk about dune recovery, does that also include dune formation on wash-over fans?
On page 17, I miss the values of the hydrodynamic conditions during the passage of the storm, and general wave (wave

rose) and tide (mean tidal range) conditions.
On pages 19 and 20, you have a grid cell size of 50 m for the bathymetry. How well can this type of raster point at geomorphic features in the inner shoreface like nearshore bars?
On page 21, you have quite nice values for volumetric percent errors!
On pages 22 and 23, you have defined the landward limit on sandy features using satellites. Why did you not do it based on your DEMs? There are some possibilities to do that solely based on that, for example (https://doi.org/10.1002/esp.5066).
On page 25, you talk about 'losses to longshore transport'. How much do you expect?
On page 26, I didn't get the value of 1.4 year for the storm-return interval.
Some references are not complete, like (6), or not proper styled, like (79).

Version 1:

Reviewer comments:

Reviewer #1

(Remarks to the Author)

Comment to manuscript NCOMMS-25-73092A

This new version of the manuscript demonstrates a clear commitment by the authors to the topic, as evidenced by the effort they have made to improve the presentation of their results and discussion. I have again enjoyed reading this work, which I find highly relevant and admirably candid in acknowledging its limitations — limitations that can only be addressed if works like this draw attention to the need for more comprehensive monitoring, tackled holistically to include all morphological units relevant to the evolution of the studied system.
I have only a few comments that the authors may use to address aspects that, from my perspective, remain somewhat underexplored or underexplained.

The authors mention that the interior of the system has gained a very large amount of sediment through the inlet, however, it is not very clear how have they reached to this conclusion given that inlets and deltas are not within the geomorphic units defined by the authors? How was this quantified and which geomorphological unit would gain sediment in this case?
The authors mention that the ebb-delta reorganised after the event, what does this exactly mean? Did the delta loss sediment and then gained again? Did the authors evaluate the changes in the deltas as well?

I really appreciate the conceptualisation of the document changes and processes in Figure 6. However, this still does not answer the question of the definition of the different morphological states. The studied area presents originally 4 morphological types (two progradation types, elongation and transgression), these are not what later the authors refer as states? I ask because the authors mention in lines 364 and 365 that morphological states control system response, which is demonstrated with their data. But, later in lines 498 and 499 they mention shifts in alternate states. What are these states, did you document a shift across any of the studied islands? To which extent a change in elevation of the dune from high to medium is a change? A clear statement about what are these alternate states is still missing. I would think this carefully as a change in state may not be driven by a change in the height only, it means more things, see for example the work by Duran and Moore (2015) or Zinnert and Stallins. In this line, in line 506, the authors mention a shift in system state, what is this new state? How do you identify the states? What are the thresholds? What is the name of this new state?

Minor comments:

In the introduction, consider using the same nomenclature for what you refer to geomorphological zones avoiding changing to environments, which is used later in the document, for instance, in the methods section, the authors use environment boundaries. Are these different from the geomorphological units?

Would it make sense introduce in the introduction section already the morphological types that are represented in your study site?

In the results (line 250), when the authors mention dune volume recovery. Have they detected the formation of new embryo dunes or was this recovery through the formation of an aeolian sand ramp? To which extent this recovery is only "dune" or could be beach? The later related to the difficulty of isolating both units and to which extent the limit of the max runup is the best option.
In the discussion, line 350, would not this result imply that washover accumulation accounted for less than one third?

The text in lines 365 to 368 may need a bit of more explanation. Do the authors suggest here or further stress the role of inlets contributing to barrier island resilience?
Lines 369 to 370: I am not sure i agree or understand. It seems that there is a bit of mixing of things here. A large immobile dune, if not changing, is because it is maintaining its resilience, correct? Only when a threshold is passed that the dune is not big anymore washover enters into action, and the dune is gone, from the point of view of the barrier it may be negative, but if you think on the time these processes take to evolve not so negative as once the dune is low it will allow and contribute to grow the backbarrier, correct? Why do we have to see high dunes as negative to the resilience of the system? It may only

happen at a different moment in time.. what I am trying to stress here is that I would not mix the fact that a breaching can be beneficial with the fact that large dunes are not, resilience may have different dimensions so it should not be considered such a straight forward consequence or simplified vision.

Line 370: not only human-stabilised inlets but also urbanised areas within the barrier islands, which prevent breaching or dunes to roll over.

Line 381 and 382: and in the end, the outer shoreface was gaining sediments, correct, maybe also worth stressing it.

Line 385: I am not sure I get "…that even washover events can drive large volumes of sediment seaward…". Washover event drive sediment inland…do you mean the same events that may induce washover are even more efficient transferring sediment offshore?

Line 390: when the authors refer to the lack of efficiency transporting sediment onshore…it seems a bit awkward, are the sediments supposed to move onshore? This might depend on the processes, but also, how do you define the limit between efficiency in the process of moving onshore and not?

Lines 429-431: this las sentence is very interesting but, i would suggest a bit more explanation to connect the previous with this conclusion given its relevance in connecting temporal scales and processes.

Lines 439-443: this part seems a bit too elucubrate, the documented differences are not so big, when you also see net import in zone 3, with zone 2 clearly lower, they are like in a gradient. However, my issue here is how can you know that one is functioning as a sink and the other as a remobilisation from deeper parts?

In figure 6. It is not clear how in zone 4 the dune is large originally and then becomes very tiny and then it remains the larger in the system? I see that the recovery in zone 4 is large, but does the conceptualisation actually reflects what you have seen?

Reviewer #2

(Remarks to the Author)
Re-reviewed by Brad Murray

The authors have addressed my comments satisfactorily, and the improved paper makes valuable contribution. Thank you for the opportunity to read about this work.

Reviewer #3

(Remarks to the Author)
Comments on the resubmission of the manuscript 'Barrier island geomorphic complexity drives storm and post-storm response', by Miselis et al.

The authors did a great job in the review of the first manuscript. The rebuttal to the first review comments was very extended (21 pages). I went trough the comments on all four reviewers and I really like the discussion on the topics addressed and the changes they made.

The introduction is now very well structured, and I like the part where they explicitly sketch two critical ways of how and why the drivers of barrier island formation and resilience are going to change. The additional Figure 1 fits also very well and gives a nice summary of a barrier island system.

It is good that the present manuscript has information about the hydrodynamics in the area. That helps to have an idea of the exposure of the barrier island to tides, waves and extreme events.

Figures 3-6 do include a lot of information, but together with the text it contains very valuable results. The results are now easy to follow and Figures 3-5 are very helpful to interpret the changes observed. The extra Figure 6 is very nice for the discussion; it really gives a conceptual explanation and is well embedded in the discussion.

The revised manuscript is worth publishing.

Rev#4 Aart Kroon

Version 2:

Reviewer comments:

Reviewer #1

(Remarks to the Author)
I am satisfied that the authors have addressed my comments, and the revised manuscript provides a valuable contribution to the field. Thank you for the opportunity to review this interesting work.

Open Access This Peer Review File is licensed under a Creative Commons Attribution 4.0 International License, which permits use, sharing, adaptation, distribution and reproduction in any medium or format, as long as you give appropriate credit to the original author(s) and the source, provide a link to the Creative Commons license, and indicate if changes were made.
In cases where reviewers are anonymous, credit should be given to 'Anonymous Referee' and the source.
The images or other third party material in this Peer Review File are included in the article's Creative Commons license, unless indicated otherwise in a credit line to the material. If material is not included in the article's Creative Commons license and your intended use is not permitted by statutory regulation or exceeds the permitted use, you will need to obtain permission directly from the copyright holder.
To view a copy of this license, visit https://creativecommons.org/licenses/by/4.0/

We thank the reviewers for their kind support of our paper and their constructive suggestions. Since they provided quite a bit of context for their comments, we have highlighted in yellow the passages in the reviewers' comments to which we responded. Our responses are directly below in gray font.

Reviewer #1 (Remarks to the Author):

This manuscript presents the results of a monitoring program that holistically addresses the response of a barrier island to the impact of a major storm as well as its recovery, although partially. For that, the authors integrate observations on the vertical changes through all barrier compartments from the backbarrier to the outer shoreface. These type of analysis, yet extremely relevant and precious, remain uncommon given the difficulties of mapping shoreface areas or even an entire island, which are in turn key to fully understand barrier dynamics and possible responses. Therefore, the authors successfully quantified magnitudes and directions of storm-driven sediment fluxes across barrier compartments and estimated rates of recovery.

The present work discriminated the magnitude of changes pre- and post-storm across the different compartments or environments within the barrier, informing about the relative importance of each one of those to the response of the barrier to the analysed storm. Through this approach, the authors were able to conclude that washover was not as high as expected. Yet, this result may raise some doubts and could probably be looked into more detail to better understand the differences between the areas with overwash and the areas without, along the zone that most likely register this process, and the characteristics of the waves impacting during the storm to better understand why is this result so different from expected. In addition, if so different, would that mean that the authors were not expecting so much transference of sand seaward?

The reviewer makes a good point here. We didn't make a good enough case as to why we would have expected more overwash. To address this, we have added a description of the hydrodynamic characteristics at the study site and during the storm to the methods section (lines 520-529) and plots of the wave conditions to the Supplemental Information (Figures S1 and S2). Further, we also reference observations from other work that show ~47% of the island experienced washover (lines 529-532). Additionally, we have backed off framing our observations as surprise in the revised discussion, which we believe makes for a more compelling presentation.

Another very relevant result relates to the fluxes and trajectories of sediment during the recovery. These results highlight the importance of large scale monitoring through the morphological continuous in order to actually understand how these systems are coping with impacts and adapting to ongoing change conditions. The accepted fact that sediment did not stay within the mapped region is also highly relevant and points to complex recovery processes, limiting our ability to understand/predict this process. In fact, these results stress our limitations to fully understanding sediment exchange across the barrier profile and lateral gradients. In this line, the present work recognises its limitations to fully map or understand sediment trajectories, especially during the recovery phase because of

the spatial restrictions.

The authors also mention that they also aim to identify modes of recovery to contribute to better understand island trajectories. However, I believe this has not been addressed by the authors, or it remains obscured because of the way the authors decided to present their results, in fact, there is a lack of discussion about processes or explanations about the observed changes, so I cannot see these modes of recovery in the manuscript in its present shape. Instead, there is a clear effort to focus on the data and avoid speculations.

While we worked hard to avoid overextending our results, the reviewer makes a good point that we did not define what we mean by “recovery modes.” To address this, we have added text to describe the different ways we quantified recovery to the results section (lines 240-246). Additionally, we have revised the discussion (lines 498-513) and added a summary figure (Figure 6), the latter of which visually integrates the results of the paper and (we believe) makes it easier to understand how recovery manifested along the island.

The present work introduces very relevant results on the impact and subsequent recovery of a barrier island to the impact of Hurricane Sandy in 2012. The relevance of the present work lays on the fact that the authors estimated sediment budgets during the erosion and recovery phases based on a series of topobathymetric surveys that extend the observations five years after the impact of the storm. The results confirm and demonstrate that recovery may be a very slow process, but also leave the possibility that total recovery (in terms of sediment budgets) may not be reached, given the losses of sediment to unknown regions. Therefore, I find this work of high relevance giving the current situation of coastal vulnerability and the lack of fully understanding of how these highly complex processes actually work. This is extremely relevant and highlight the importance of extensive monitoring programs, not very common, but key to actually understanding system response at a large temporal and spatial scale. The work targets knowledge gaps related to data scarcity, namely data integrating changes across and along the morphological continuous.

Overall I believe this is a very important contribution as it addresses a very important problem, not so well understood and proves the importance of this type of work, particularly, it demonstrate the value of monitoring programs and sediment budget estimates to actually understand the response of such complex settings.

This type of work is rather uncommon because of the scarcity of this type of data, through space and time, focusing on the impact and recovery after a storm. Most examples looking into the impact of storms at the scale of an island use to focus on the impact or erosive phase, because of the related risks and loss of geomorphologic capital. Rare are the works monitoring the whole process and in fact, as demonstrated in this work, recovery may be such a long process and has not raised the same attention despite of being equally important. For instance, a work that could compare, because of the type of data and coverage of the cross-shore profile, can be found in Ruiz de Alegria et al. (2025). The authors also focus on understanding exchange between different profile/barrier

compartments, extending to the shoreface. Some differences are worth noting between both works that further highlight the relevance of the present work, these are the spatial scale (an attempt to close a morphological continuous was done in the present work), the type of coast, which the former is located in an embayment, the present is located in an open coast, which turns any attempt of sediment budget estimates, far more complicated. Yet, both works find that the outer/offshore limit of their AOI may not be capable to capturing possible exchanges with deeper areas, a pinpointed knowledge gap.

We thank the reviewer for pointing out this relevant work. We have incorporated it into the revised discussion (lines 378-380; lines 408-411).

The results do support the conclusions, although they could be presented/discussed in an even simpler way, specially focusing on explaining the implications of the morphological state to the response of the zone to the storm and recovery. The authors claim that the morphological state of each zone of the island is key on the type of response observed. However, the way the results are presented does not shows clearly how the state roles the response and additionally, I see this as a kind of chicken-egg problem. A morphological state is achieved because of a dominant sediment flux and resulting budgets that result from the interaction between the sediment supply and hydrodynamics, so, to which extent the results are only reinforcing a state that results from a lack (excess) of sediment rather than the state controlling that lack (excess) of sediment itself or the fluxes of sediment? Said that, I would like to reinforce the fact that the results are a bit confusing and long, without clear outcomes. The only thing that is clear is that the transgressive zone favours the inland transfer of sand, through overwash and inlet breaching/maintenance, but the relation with the outer shoreface, key to the whole problem, remains unclear. Also, the elongation zone seems to gain sediments faster, which is expected given that it is at the end of the littoral drift. But, what about the progradational zones? How do they respond? It would help if the major behaviours of response (modes) are associated with a morphological type in a simplified way, this could help understand the conclusions of the authors.

There is a lot to unpack here, so we've attempted to distill the comments down to address the underlined sections:

1) *"The authors claim that the morphological state of each zone of the island is key on the type of response observed. The way the results are presented does not shows clearly how the state roles the response"*

Prior to submitting the first version of the manuscript, we played around with a number of different ways to organize our very dense results, so I think we can confidently say that the way they are currently organized makes the most sense to us. In general, we have tried to discuss the overall island response we observed (e.g., "All AOIs") and then how the responses varied for each geomorphic zone. For example, for the storm period volume changes, we talk about the overall island response (first paragraph; lines 137-148) and then how the responses within each geomorphic zone differed from that overall response (second paragraph; lines 150-167). We follow the same formula with the post-storm volume changes, talking about the overall response of the island first (lines 171-179), followed by differences between zones (lines 181-200). We follow that with our presentation of net volume changes (not

separating the storm and recovery periods) and post-storm recovery. Since we calculate “recovery” differently than just quantifying the post-storm volume changes, we wanted to make sure these stood apart and we combined them with the net losses because the overall loss of sediment from the AOIs (net loss) likely influenced the lack of recovery we observe. Lastly, we are careful to distinguish between volume changes and fluxes since they are also calculated differently.

2) *"I see" the morphologic state controlling the response "as kind of a chicken-egg problem. A morphological state is achieved because of a dominant sediment flux and resulting budgets that result from the interaction between the sediment supply and hydrodynamics, so to which extent the results are only reinforcing a state that results from a lack (excess) of sediment rather than the state controlling that lack (excess of sediment itself or the fluxes of sediment?"*

Barrier island geomorphic state is absolutely the product of sediment fluxes that occur over decades or longer (lines 27-28). Our ability to know what the dominant fluxes of the past may have been depends on how well flux-generated morphology is preserved (e.g., beach ridges; Ciarletta et al., 2019 https://agupubs.onlinelibrary.wiley.com/doi/full/10.1029/2018JF004908). Storms, though more instantaneous, have the ability to move the barrier between morphologic states, by leveling dunes to create more opportunities for washover or by breaching the island to build up back-barrier elevations (lines 28-31). Whether modern, storm-related flux pathways reinforce the pathways of the past would depend on whether the physical drivers of those fluxes are the same through time, which is rare (lines 31-32). In completely natural systems, shoreface fluxes may change due to the opening and closing of inlets (Ciarletta et al., 2024) or the availability of shoreface sediment which is not uniformly distributed (Miselis and McNinch, 2006; Locker et al., 2017; Wei and Miselis, 2019). But now, particularly when climate change is altering background sea level and storm conditions and when humans are passively (Ciarletta et al., 2024) and actively altering sediment transport pathways (Anarde et al., 2024), it may not be a good assumption to assume that modern storm events reinforce past sediment transport pathways, particularly if the geomorphic state of the barrier itself is altered by the storm. So, the reviewer has identified the very basis for what we are attempting to explore in this paper: *how* morphology preserved from past fluxes influences modern storm and post-storm fluxes (lines 41-44).

3) *“The results are a bit confusing and long, without clear outcomes. The only thing that is clear is that the transgressive zone favours the inland transfer of sand, through overwash and inlet breaching/maintenance, but the relation with the outer shoreface, key to the whole problem, remains unclear."*

Though we will acknowledge that the results are very dense, we disagree with the reviewer that the only finding is that the transgressive zone favors the inland transfer of sand. Since the reviewer doesn’t offer specifics, we assume that they want to see something similar to what Reviewer 2 suggested, which (to paraphrase) was to better describe the novel and important findings and pare down statements about what the observations imply. We believe the revisions to the discussion and a new summary figure will satisfy the reviewer’s confusion, since we have better framed the role of overwash in the context of landward and seaward fluxes (lines 342-411) and have expanded the discussion of the role of the shoreface where we put it in the context of sediment availability (lines 413-449).

4) *“It would help if the major behaviours of response (modes) are associated with a morphological type in a simplified way, this could help understand the conclusions of the authors."*

It would be lovely if the results were straightforward enough to assign specific responses to specific morphological types! But the very nature of our results makes that complicated. For example, we observed the most washover deposition in the transgressive zone, but even so, 71% of the storm-period deposition in that zone occurred in the outer shoreface. So, it would not seem appropriate to call that behavior “washover-dominated.” Further, because Zone 2 was so heavily impacted by the inlet, it would also not seem appropriate to say that the behavior we observed would be typical of a historically

progradational barrier. But the reviewer's point is well taken that we say "response modes" without ever defining what we mean by that exactly. And since the biggest differences in the response were measured in the post-storm period, we have defined what we mean by overall recovery, recovery distribution, and rates of recovery in the second to last section in the results section (lines 240-246)). Further, we have added a summary figure (Figure 6) that shows the volume changes we've measured in the context of dune volume changes throughout the survey period and we think this captures the morphologic effects of our observations in a much clearer way.

Another aspect that remains a bit confused is the balance of sediment. From what I understand, a very large part leaves the region, however, given that the authors only calculate the changes for the AOI, could there be a possibility that part of the sediment could be in the areas not calculated but still within the island region?

Yes, because we analyzed only changes in mostly natural areas, it is possible that sediments missing from the AOIs could be found beyond the AOI boundaries (either downdrift or offshore of them), and we acknowledge this at several points throughout the paper (lines 146-148; lines 177-179; lines 187-189; lines 354-357; lines 396-398). Unfortunately, it would be impossible to parse natural recovery from human-facilitated recovery outside of our AOIs given the volume of sediment placed in the system and the fact that humans may move it around more after it is in place. That said, our measurements spanned almost all of Zone 2 and we had 3 different AOIs in Zone 3, so coverage of those zones was pretty extensive. Natural areas were very limited in Zones 1 and 4. In Zone 1, our AOI was at the westernmost boundary of the zone, making it unlikely that sediments would escape updrift, against the direction of net longshore transport. Our AOI in Zone 4 did not share a similar positioning and it is the most likely Zone where we would expect sediments to accumulate, particularly given Zone 4's position at the littoral end point of the island. But even if much of the sediment missing from our AOIs is there (or out of the system altogether in Fire Island Inlet), it will not be that helpful to the recovery of the rest of the island since it would be nearly impossible for that sediment to be transported against the net longshore transport direction in significant enough volumes to reverse the losses updrift.

One more aspect that is not considered by the authors, though estimated, are the errors. Some of the changes seem to fall within the error range but this is not mentioned across the main sections of the manuscript. I believe this is highly important and could help to understand better the limitations of such work, but also, some of the missing volumes?

We initially felt that reporting the percent errors was preferred over adding to what were already complicated figures, but we see now that the lack of error bars impedes comprehension of the results. We have now added propagated percent volume errors to all of the figures. It should be noted that the percentages in Figure 2 are not related to specific volume change measurements, but rather the relative percent of the total erosion or deposition measured as denoted by the font color (as described in the figure caption).

It is not easy to understand or find how much sediment arriving at the outer shoreface stayed there or leaved from the volume transferred during the impact of the storm. This could be improved so the results can be followed in an easier, more direct way.

The reviewer's point is well taken here. Though we did say on lines 174-175 that only 33% of the sediment eroded from the outer shoreface can be accounted for by deposition in other AOI environments, we did not specifically state, across all AOIs, whether any of what was deposited on the shoreface remained there. We have revised the results section on lines 175-179 to indicate that, across all AOIs, about 18% of the storm-generated outer shoreface deposit was not eroded from the outer shoreface in

the post-storm period. This means that almost half of the outer shoreface storm deposits are unaccounted for within our AOIs. We discuss the implications of this in the revised discussion (see lines 390-411). Sounds like there is more work to do!

I find that it seems difficult to generalise responses, but maybe the responses are in general not so different? The main difference is the recovery, I would put emphasis to this and the balances, and the fact that zones with high dunes cannot have overwash, but how this means they are less resilient or that show less resilient responses? Are the zones with high dunes eroding (losing sediments) more than the others? If barriers have wider zones with high dunes, it means they have not yet reached the transgressive state, and this also means that they may eventually reach it, if sediment recovery does not work. Now, if nourishment prevents this, the shift of state is delayed and then, the question is, as the authors also mention, until when humans will be able to hold the line.

The reviewer is correct that it is difficult to generalize responses…there is a lot going on! The way we did so was to combine all the measured changes across all AOIs, which is presented at the far left of Figures 2, 3, and 4. And when we introduce the differences between zones, we often do so by pointing out how the individual zones differ from that generalized response. We also think that the revisions to the discussion (see lines 498-513) and the addition of the summary figure (Figure 6) helps address the reviewer's questions about whether dune size impacts fluxes and how and whether morphologic states are changing.

Finally, I believe that the authors could give more information regarding the offshore limit or boundary of their analysis domain as this seems to be a possible limitation to the closure of the budgets. In this line, I would appreciate if the authors could explain better how they obtain the estimated DoC they use to delineate the seaward limit of the inner shoreface, or to justify that the offshore boundary of their domain falls within the possible extent of sediment movement. After checking some of the results for this island using WIS stations, the annual averages are far larger than the ones suggested by the authors in the present work, but what is more important, given that Sandy was a storm with a very large return period and quite uncommon, I would expect a deeper DoC, this needs further justification/explanation.

We agree with the reviewer that we could do a better job of explaining how we arrived at the justifications for our shoreface boundaries and how we calculated the estimated depths of closure (DoCs). We did not choose the seaward boundary of the outer shoreface. It was chosen for us by the offshore extent of the pre-storm lidar survey, which extended to an average water depth of -8.6m. Since collecting data from the shoreface is so difficult, it is common that a target depth is not always achieved. This simple fact was acknowledged in the citation suggested by the reviewer above (e.g., Ruiz de Alegría-Arzaburu et al., 2025), where the deepest limit considered for that volume analysis was 8m despite many of the profiles extending to 10m. So, despite all of our post-storm shoreface surveys extending out to about -10m water depth or deeper, we can only track sediment where we have data, so the pre-storm lidar footprint is our volume limitation and de facto offshore boundary. We have revised the text to expand upon our previous direct acknowledgement of this limitation on lines 646-650 in the Methods and provide more details on how we tailored our estimated DoC for the period of observation (lines 650-665). We find that the average offshore boundary depth is about 2 m deeper than our estimated DOC for the period of observation and about 1.5 m shallower than an estimated DOC for Hurricane Sandy. However, we frequently do not see much significant change beyond -8.5m in profiles, likely due to the limits of the resolution of the instrumentation. Where we do see possibly significant changes, they are much smaller in magnitude than

the changes in shallower water, so we feel confident we are capturing the majority of the resolvable volumetric change within our outer shoreface boundaries. Furthermore, if we didn't have this limitation, outer shoreface volumes would be likely to increase even beyond what we already measured during the storm period, which would reinforce the patterns we are seeing with the data limitations we have.

For the inner shoreface boundary, which we use to separate inner and outer shoreface environments, we use the same wave time series for the period of observation and use ALL of the waves to calculate the DOC, not just the ones above 2 m (lines 668-671). We believe this contrast is clearer now with the existing revisions to the preceding paragraph. This approach is justified because the majority of the assessment captured much calmer wave conditions during the post-storm period. So, using only extreme wave conditions associated with the storm to define the boundary between the inner and outer shoreface may have impacted our ability to resolve shallow-water focused post-storm changes.

In this line, it is also worth noting the net import that the authors document during the storm, however without a clear clue of the origin of the sand. Did the authors consider that given the relatively small proportion related to the error (6% compared to circa 5% of the error)? To which extent, the fact of not including the whole island could help to explain this mismatch? Yet, this is a very interesting result, once certain that is not obscured by errors, as it would imply that the impact of the storm brought sand to the studied AOI. Not necessarily what one would expect.

As we noted above, we initially felt that reporting the percent errors was preferred over adding to what were already complicated figures, but we see now that the lack of error bars impedes comprehension of the results. We have now added propagated percent volume errors to all figures. For figure 4b specifically, they still do not exceed the measured percentages of import/export. That said, we were expecting that erosion and deposition would be more closely balanced across barrier island environments during the storm period, since cross-shore processes are more dominant during storms and our analysis design is setup to capture that nicely. And we fully expected that within-Zone imbalances would be particularly evident during the post-storm period when alongshore transport likely dominates and humans were adding sediments all along the barrier that would result in significant morphologic adjustments. But we agree that it is interesting that the storm resulted in a net import of sediment in some of the zones, most significantly in the transgressive and elongational zones. And we're pretty excited about the now revised parts of the discussion where we link morphological differences in dune heights and geologic differences in shoreface sediment availability to the observed responses (lines 433-449).

The methods are ok, yet, I would appreciate a bit more development of the limitations of the shoreface survey given the boundary limit and the spatial resolution. How certain the authors can be of their results across the shoreface given that the resolution is 50m, is this enough?

Shoreface survey resolution is never enough! We would always love to have more data from the shoreface and we see our paper as a(n imperfect) demonstration of the value of these data that will underscore the need for them and hopefully lead to analyses using more dense, higher resolution shoreface data in the future. And we also acknowledge, as the reviewer points out, how critical this information is for understanding storm and post-storm processes, so we sought to present a kind of "reduced-complexity" observational approach that could match the resolution of the data we had. The analysis essentially explores how much sediment is in one box or another, which does not require detailed morphologic information (e.g., sand bar and trough shape) that might be expected from higher resolution data. So, the nature of the analysis lends itself well to sparse data, possibly increasing the

portability of the methodology since shoreface data are so difficult to acquire. We have added a few sentences to the methods to acknowledge this (lines 577-583).

A bit of more detail could also be added regarding the approach to estimate the volume changes, namely in the case of the overwash given the presence of channels and bushy vegetation.

Back-barrier vegetation is a known source of lidar elevation error. The lidar metadata did not report a separate error value for vegetated areas, so we are left to quantify uncertainties with the information available to us. And though the presence of vegetation would skew pre-storm elevations higher, possibly resulting in underestimates of washover volumes, there are a few reasons that pre-storm elevation uncertainties may be less of a concern in our study area versus others. First, the pre-storm survey (May 2012) occurred in the spring, when vegetation would not be in full leaf, likely helping to reduce uncertainties. Further, the characteristically low, scrub shrub vegetation (as opposed to forests or marsh) is often bisected by bare sandy paths, which could provide bare-earth elevation verification within the vegetated zones. Lastly, we were able to measure deposition in the storm-period back-barrier environments, suggesting that the storm signals were large enough to capture with the sensor despite any inherent errors. Notably, our observed washover volumes were the same order of magnitude as field-based measurements of washover thickness (Hapke et al., 2013; https://doi.org/10.3133/ofr20131231), despite different sources of uncertainty associated with that dataset.

For the post-storm period, when we expect back-barrier elevation changes to be much smaller and therefore harder to resolve, our methodology is tailored to reduce elevation uncertainties. Because we use the presence of sandy areas (e.g., overwash extents) in post-storm satellite images to define the landward boundary of the back-barrier environment, the issue of vegetation is much less of a concern for the post-storm period. So, even though the post-storm changes should be harder to resolve, elevation uncertainties due to vegetation should be minimized because we restrict the environment to the sandy overwash areas where bare earth lidar accuracies apply. We have added a sentence to the methods (lines xx-xx) to point this out and to satisfy a comment from a third reviewer.

It would be also interesting and key to the conclusions, to understand to which extent the results from the few AOI, which cover a very small proportion of the whole island, are representative of the overall changes.

This was one of Reviewer #2's comments as well. See more detailed response there. We added a brief justification for our assumption that the AOIs are representative of each zone to the introductory paragraph of the results section (lines 694-697).

As mentioned earlier, the estimates to the DoC could be better explained given the importance of this parameter to the understanding of the cross-shore sediment exchanges and the very different estimates they get for their study period compared to estimates available online (Atlantic_DOC_yearly_0116.xlsx retrieved from the U.S. Army Corps of Engineers).

See response to a similar comment above (p. 6).

Smaller details:
When deciding the best approach to define the compartments, a recent paper could be

also useful for the authors, see https://doi.org/10.1016/j.geomorph.2025.109610 from Bauer et al., 2025.

We thank the reviewer for this suggestion. It's interesting to see that other authors have also landed on a "zero-change" boundary to help get around the subjectivity associated with identifying dune toes and heels to make methodologies more portable. We cited this work to help support our choice for our dune limit boundary (lines 680-683).

In figure 2.A, some of the % of deposition or erosion do not add 100.

This occurs in Figure 2a (All AOIs; Zone 2) and 2b (All AOIs; Zones 2 & 4) due to rounding to avoid decimal percentages. A sentence has been added to the caption to explain this.

In figure 3.C, does the net change within the shoreface include the outer shoreface?

Yes, it includes everything within our AOI boundaries for the shoreface. This is specified on lines 780-786 of the text in the Methods section.

The authors tried in some cases to generalise some patterns, like in line 145: "Those percentages decreased downdrift in Zones 2-4...", however, this is not what the results indicate, with Z4 being higher than 2 and3 and very close to 1.

The reviewer is correct that the way this sentence was written is misleading. We have updated it to read (lines 154-156): "Lower percentages were observed in Zones 2-4 (Figure 4a) leading to alongshore differences in the proportion of storm-period washover deposition from as high as 29% and as low as 1% of total deposited volume (Figure 3a)."

There is still one more question that the work raises and the relates to the period of analysis, I imagine that new storms impacted the region over the five years of analysis, sending backward the recovery trend, was this the case? if so, this should be stated and the implications discussed.

Yes, the reviewer is correct that several storms impacted the region during what we define as the post-storm period, but none were as strong or as long in duration as Hurricane Sandy. All were typical of storms that affect the study site from November to April (extratropical storms, locally known as nor'easters) so can be considered part of the seasonal signal in the data. Therefore, we consider the period of observation typical of the conditions in which recovery would usually occur in this system. Because wave conditions were of interest to another reviewer as well, we have added some figures to the supplemental information showing observed significant wave heights and dominant periods spanning our observations (Figures S1 and S2). We should also point out that we know from earlier work that beach elevations started rebounding around Sept. 2013 and, with a few exceptions, continued to recover through Sept. 2015 (see Figure 8 from Brenner et al., 2018), suggesting that recovery signals can be observed outside of any changes affected by seasonal storms. It is possible that these seasonal storms mobilize sediment otherwise trapped on the outer shoreface and move it into the terrestrial system (as observed by Sherwood et al., 2023 in Core Banks, NC, USA), but this is just speculation since our data is not of the temporal resolution to resolve those processes.

It would be very helpful is the outcomes on response patterns are attributed to each

morphological state in a simple way, in its present shape, the reader cannot easily see the implications of the state to the response patterns.

The reviewer brought this up in their general response and we have addressed it there (pg. 4).

I believe that it would be interesting if the authors also consider the possibility of the sediment not returning to the system. Also, the estimates are interesting but based on a linear response, how certain are the authors that the recovery will continue as for the first five years? Again, information regarding possible storms during the "recovery" or post-storm might be worth information to support the low rates of recovery, or not, but valuable information.

In our revised discussion, we more explicitly discuss the implications of the fact that during the post-storm period, much of the sediment eroded from the outer shoreface is not returning to the island environments (lines 390-411). As for whether recovery is a linear process, our observations make that seem unlikely (see new Figure 6). We would expect that recovery would taper off as the system worked it's way back to a new equilibrium, but with humans actively manipulating sediment supplies, this is impossible to discern with our data.

The authors mention in Line 319 that Z1 recorded the greatest flux from the beach. I think this should be more carefully explained as this is relative to the % eroded from the rest of compartments, but it does not necessarily mean that the volumes were larger, is this correct? So, the way it is presented it may appear that the beach here provided more sand, but this is not that simple, correct? Also because the larger proportion of eroded sand did not come from the beach but inner shoreface.

Here, it seems the reviewer is incorrectly interpreting Figure 2. The size of the bars indicates the magnitude of the volume change (normalized by alongshore length because our AOIs are different sizes), whereas the percentages indicate the percent distribution of either volume losses or gains. So, the bigger the bar, the larger the alongshore normalized volume change. Comparing the orange bars between all of the zones, the biggest orange bar is observed in Zone 1 and is negative, indicating that Zone 1 experienced the largest flux of sediment from the beach.

Lines 326 to 330 regarding fluxes across Z4. Is the statement correct? Not so easy to see in the figure, and when talking about shoreface, you mean inner one?

This is a good catch by the reviewer. Even with all of the revisions to the discussion, the statement has been corrected and now reads (line 468-470): "Conversely, in elongational Zone 4, we measured the lowest storm-period fluxes from the beach and the highest fluxes from the inner shoreface and to the outer shoreface (Figure 5a). However, we don't see similarly elevated shoreface fluxes in Zones 2 and 3 (Figure 5a) despite the similarity in dune heights between the AOIs representing Zones 2-4 (Figure 2e)."

Lines 336 to 338, the authors affirm that the results highlight how the shoreface sediment availability may influence sediment fluxes, however I think this could be further discussed/explained.

We appreciate this suggestion from the reviewer. In response, we have expanded the discussion of shoreface sediment availability in the discussion (lines 413-449) and we have added a figure to the supporting information (Figure S3) that we hope will provide some much-needed context.

Line 352, the authors mention that rapidly building dunes may impede transgressive processes. This is a bit confusing, in the first place, if a dune grows rapidly is because it is in a prograding state, in this case, I cannot see the need to become transgressive. On the other hand, if the system is close to a shift of state, I cannot easily imagine a dune building rapidly, what do the authors actually try to state here?

We can understand the reviewer's confusion here. The connection between management practices in the first part of the sentence does not convey to the second part of the sentence, where we meant that human-built dunes that are created rapidly after storms, not natural rapid dune building processes. The sentence has been rewritten to convey this more clearly (lines 489-492).

Line 345, the authors mention that alongshore and shoreface sediment supplies are maximised. If so, to which extent is a state control rather than gradients of longshore sediment transport controlling the patterns of barrier response, and even the long-term barrier state?

This appears to be a time scale issue. There is no question that gradients in longshore sediment transport shape barrier response and the long-term barrier state. The point we make here is that antecedent state transcends to shorter term behavior as well, such as recovery processes, and that this is important to consider when managing coastal sediment supplies.

The authors mention that during the post-storm period, only 33% of the sediment from the shoreface resulted in sedimentation in inner barrier compartments, where is the remaining 67%, still in the shoreface? How much remained?

The reviewer brought this up in their general comments as well and it is addressed there (pg. 5).

Reviewer #2 (Remarks to the Author):

Review of ‘Barrier Island Geomorphic Complexity…’
By Miselis and others
Reviewed by Brad Murray

First, I enjoyed reading this manuscript, and I thank the authors for doing this valuable work. I think this work should be published, and to increase the paper’s impact, I suggest two ways to improve the framing and presentation of the work.

The most important structural change in the way the work is described, it seems to me, involves the conceptual assumptions that seem to underlie the way the analyses are presented. As a brief recap, the Authors have conducted extensive (and valuable) observations in Areas of Interest (AOI) arrayed along the Long Island coastline. These AOI are positioned within zones that differ from each other in their geomorphic characteristics.

In an ideal world, the AOIs would be as large as the zones, so that the two terms would be synonymous, although anthropogenic influences and, I suspect, finite resources for the observational work restrict the observations to the AOIs that are subsets of the zones. In the present manuscript, much of the results and discussion implicitly equates the AOIs to the zones, seeming to assume that changes in the AOIs are representative of their respective zones. For example, if the AOI in one zone looses volume during a time period (from one or more of the environments—beach, dunes, etc.) it seems to be assumed that the result would apply to the whole zone (if observations were able to cover the whole zone). It might be true that the findings in each of the AOIs are representative of the whole zone they are meant to represent, but we as readers don't know if that is the case. This ambiguity raises questions about interpretations that are implicitly based on the assumption that observations in the AOIs can be extrapolated to their respective zones. (The sentence beginning on line 156 provides an example: "Every other zone experienced net import, or sediment transport from outside the AOIs, the highest of which occurred in Zone 1.") I can imagine that sediment lost/gained within an AOI could be sequestered/sourced elsewhere within the zone the AOI is mean to represent (e.g. through alongshore-heterogeneous patterns of shoreline change caused by subtle curvatures, although this is just one possible mechanism). The assumption that observations in AOIs can be extrapolated to the larger Zones should be made explicit, at least, and the bases for that assumption should be clearly described, early in the paper.

We thank the reviewer for pointing out that we didn't explicitly state how or why the AOIs are representative of the larger geomorphic zones. In panels D and E in Figure 1, we did show how the average and range of dune crest elevations compare between the geomorphic zones (Figure 1d) and the AOIs (Figure 1e), but we never explicitly discussed this comparison to defend this very important assumption of the paper. To address this, we have added an introductory paragraph to the results section where we briefly describe the geographic and storm characteristics, the zonal geomorphology, and now our justification for our assumption that the AOIs are representative of each zone (lines128-133).

I also suggest that the Authors rewrite the paper to highlight the findings that suggest new insights about barrier processes, and reduce relatively vague references to the complexity of barrier responses to storms. For example, I think the paragraph starting on line 359 (about 'surprisingly' small overwash fluxes during a storm with characteristics that would be expected to produce a lot of overwash) describes novel and important findings that could be emphasized by paring down the other statements about what the observations apply.

We have reorganized and rewritten this section of the discussion to better link the overwash observations to the sizeable seaward fluxes we observed (lines 342-411). We believe the comparison makes the paper stronger and we thank the reviewer for the suggestion.

Similarly, the content of the paragraph starting on line 376, about the importance of inlets, the opening of inlets, and interactions with human management practices, would stand out more if the implications were pared down to the clearly novel aspects of the work.

This discussion has been shortened and incorporated into a discussion of landward and seaward fluxes (lines 353-371). We think this improves the messaging of this section considerably and thank the reviewer for their suggestion.

And as an example of a relatively vague statement that could refer to aspects of barrier response to storms that are already understood, the sentence starting on line 157 states: “These results demonstrate that barrier geomorphology influences the magnitude and proportion of volume changes and the balance between cross-shore and alongshore sediment transport processes during storms.” I think that statement is true, but it might hinder the paper’s effectiveness, because some readers might get stuck wondering what these results suggest in concrete terms, and whether that is different than what is previously understood. For example, statements like this might lead a reader to picture the well-understood dynamic that where dunes are low enough for a storm to produce overwash, cross-shore fluxes are high (and the ration of cross-shore/alongshore transport is high), compared to location where dunes are high and cross-shore flux could be limited to seaward transport to the lower shoreface. Highlighting the most novel contributions will make the paper easier to read and more effective.

The point the reviewer makes is well taken—in our attempt to summarize our very dense results paragraph by paragraph, we may have erred a bit toward oversimplification in some places. We have edited the sentence in question to read (lines 165-167): These results demonstrate that alongshore variations in barrier geomorphology influence how sediment reservoirs in barrier environments behave and interact during storms.

The paragraph starting on line 433 is misleading and should be removed. This paragraph makes the point that, compared to what is represented in models of long-term barrier dynamics, the observations indicate a more complicated pattern of sediment fluxes, including both onshore and offshore fluxes. I don’t think the observations are relevant to barrier-dynamics models in this way, because the models represent long term sediment fluxes that are meant to average over the timescales of individual storms and post-storm recovery. It is known that the storm/recovery cycle involves alternations of offshore and onshore flux on the shoreface, but the longer-term models don’t explicitly represent changes on those timescales. Complicated patterns of flux, including offshore flux during storms, is consistent with the supposition that the net long-term fluxes are all landward. It might not be true that patterns of long term flux are as simple and uniformly onshore as is assumed in models (it almost certainly isn’t that simple in some natural systems), but observations over the timescales involved in this study should not be compared to long-term modeled fluxes in the way the paragraph in question does.

In the revised discussion, this paragraph naturally fell out as unnecessary. Kudos to the reviewer for seeing that first.

Minor comments and suggested edits:
Line 14: “…barrier island trajectories, yet attempts…’ (add a comma)

Added.

Line 34 and 44: Make the references for ‘First’ and ‘Second’ clearer. Perhaps the last sentence of the first paragraph (line 32) could end with ‘…timescales are changing, for two reasons.’

Good suggestion. Have added “in two critical ways.” to the end of the sentence on line 32.

Line 73: Rework the sentence starting with ‘Recent work demonstrates…’ to make clear that you are referring to sediment fluxes from the subaerial barrier to the shoreface via ‘outwash’ from the backbarrier to the front.

Agreed, have edited this sentence to make this clearer (line 75-77).

Line 111: Although I am not offering specific ways to accomplish this, it seemed to me when I reached the end of the Introduction that that section could benefit from some tightening. Perhaps you could look at that section to see if any reorganization or condensing could improve it?

The introduction has been lightly revised, particularly looking to streamline the language.

Line 119: ‘…is comprised of…’

Revised.

Line 137: RE ‘Accumulation …exceeded losses… by about 6%.’: Are you confident that the observations are precise enough to justify this interpretation, or should a caveat be added to point out that the difference could be within the error (based on Figure 2)?

As we noted above in our response to Reviewer 1, we initially felt that reporting the percent errors was preferred over adding to what were already complicated figures, but we see now that the lack of error bars impedes comprehension of the results. We have now added propagated percent volume errors to all figures. For figure 4b specifically, they still do not exceed the measured percentages of import/export.

Line178: RE “In Zone 1, the growth of the ebb tidal delta reduced downdrift losses …”, please elaborate; what mechanisms envisioned?

We were seeing this from an alongshore transport perspective, where there is more sediment coming in (updrift nourishment) and less sediment going out because the presence of the ETD disrupts “normal” LST pathways in favor of slower inlet bypassing pathways. We’ve edited this section to make this clearer (lines 184-193)).

Line 190: RE “… and it’s position near the littoral end point of the system.”—please elaborate about what mechanisms are envisioned.

We have edited the sentence to read (lines 213-215): “…it’s position near the littoral end point in the system, where it is more likely to benefit from sediments derived from other zones.”

Line 191: Clarify 'was distinct)

Changed from "was distinct" to "varied spatially." (line 216)

Line 194: RE 'This suggests…', please elaborate on what you mean by 'geomorphic history'. (I can guess, but I don't know if I would be right.)

This sentence has been edited for clarity (lines 218-221).

Line 210: Please elaborate on '…naturally cascading sediment transport'.

We have added language to this sentence to clarify (line 234).

Line 295: RE '…volumes of sediment exported from … Zone 2…': Couldn't this also be 'not imported into'? I think all statements like this should be rephrased in terms of net fluxes (rather than explicitly as imports or exports).

In this case it does not make sense to rephrase in this way, because the point we're making is that we are NOT seeing recovery that we might expect in Zone 3 despite the losses measured in Zone 2. In other words, despite the net flux out of Zone 2, we are not seeing a comparable net flux into Zone 3 (at least in the dune environment).

Line 308: Change to '…barrier-system scale…'

Good catch. Corrected (line xx).

Line 332-334: The AOI isn't at the end of the system (even if the zone is)—an example of how Zones and AOIs need to be disentangled in the interpretations.

This was part of the reviewer's general comments and has been addressed above (pg. 12).

Line 336-338: RE '…these overall findings highling how shoreface sediment availability may influence the magnitude of sediment fluxes…': Please elaborate. What do you mean by 'shoreface sediment availability? Is there a limited veneer of mobile sediment on the shoreface? Or does this refer to limited rates of onshore flux from the lower shoreface?

We agree that we were expecting the reader to know a lot more about this than was appropriate. We have happily rewritten the discussion to include more information about shoreface sediment availability (lines 413-449), including a new figure in the supporting information that provides further context (Figure S3).

Line 344: Please clarify 'alongshore cascades in sediment transport and morphology'

This language has been edited out.

Line 371: Please elaborate '...where dune and sediment supplies are low'

This statement has been edited out of the discussion.

Line 396: specify '...the inlet-induced geomorphic state change in Zone 2' (i.e. erosion of dunes)

In the revised discussion, this sentence now reads (lines 362-365): "Second, inlet-induced dune erosion in Zone 2 resulted in almost 4 times as much washover volume in the post-storm period compared to the storm period (Figures 3,6) demonstrating how changing barrier geomorphic state dramatically influences barrier-system sediment flux magnitudes."

Line 401-402: Reword '.. a commitment of the barrier to retreat...' which anthropomorphizes barrier dynamics

This statement has been edited out of the discussion.

Reviewer #3 (Remarks to the Author):

Dear authors,

(General comments)
The manuscript 'Barrier island geomorphic complexity drives storm and post-storm response' by Miselis et al. deals with the impact of storms on a barrier system in New York. The paper is well structured and well written, and the topic is very relevant for those who must manage our shorelines.

The novelty of this paper is certainly the inclusion of the shoreface in the volumetric analysis. In their case, this means that they now have better ideas of the total budgets in four specific areas in Long Island, New York.

One of their main conclusions is that the amount of landward transported sediment towards the hinterland is far less than expected from previous studies. This has of course a major impact on the management of the shores on longer time scales (decades). They stated their conclusions at the end of the discussion and in the abstract, but not explicitly in a paragraph with conclusions.

We have rewritten the discussion to put our main conclusions in better context, including our discussion of landward (e.g., overwash) and seaward fluxes (lines 342-411). Further, we have also added a summary figure (Figure 6) and a paragraph at the end of the discussion sentence as a means to tie the various conclusions together (lines 498-513).

Overall, the manuscript is of good quality. The introduction deals with many aspects related to the topic and the introduction is well embedded in state-of-the-art literature. For clarity to all readers, I would suggest including Figure S1 in the main manuscript. For non-

coastal scientists, it immediately explains all terminology used. The discussion is also nice and includes many good points!

We thank the reviewer for their kind support of our paper. We agree that Figure S1 provides good background and will make the content more accessible. We have removed it from the Supporting Information and made it Figure 1.

After reading the whole paper, there are only three aspects that I miss in this manuscript which have relevance for the study.

The first aspect is the question of what you expect to happen during a storm: overwash or breaching of the barrier. There are barrier islands where breaching is often occurring and where the associated landward fluxes of sediment during the storm are mainly governed by that. Nienhuis et al. 2021 (https://doi.org/10.1029/2021JF006066) give a first attempt to see if your barrier is vulnerable for that.

Both breaching and overwash occurred at our study site, but we agree with the reviewer that we could be more explicit about this. In response, we have added some text in the introduction (and the suggested citation) to point out that future conditions might change the likelihood of overwash versus breaching (lines 37-38). Also, we have added some text to clarify the occurrence of overwash and breaching in the introductory paragraph of the methods (lines 529-532). And in response to the suggestions of other reviewers, we have revised the discussion to address the partitioning of landward and seaward fluxes more carefully (lines 342-411), which we hope helps to clarify the role of each process in our observations.

The second aspect is the influence of dune development on wash-over fans in the post-storm recovery phase. After the initial formation of a wash-over fan, you quite often see dune development on these sand bodies in the period after the storm and this is often with sediment coming from the adjacent beaches. The volumes are significant and can build up a dune area within decades and changes the sensitivity for erosion of coastal stretches. This is for instance written in https://doi.org/10.1002/esp.4610.

Yes, the possibility that new foredunes might form landward of where they were before the storm was why we developed a methodology to extend both the dune and back barrier environment boundaries landward in the post-storm period by establishing a morphology-based buffer and extending it from the storm-period boundaries (Figure S10). Any foredune formation that might have occurred between the post-storm upper beach change envelope (representing an estimate of the upper limit of wave incursion) and within some distance of the landward extent of the washover fans would be accounted for by our methodology. We do measure positive post-storm volume changes in the dune and back-barrier environments (Figure 3b) though it is not clear from the change maps whether a coherent foredune is appearing (Figures S4-S9). But dunes often build over decadal time scales, so it is possible that these positive volume changes represent some early or intermediate phase of dune building as detailed in the citation provided. We have added a sentence and a citation to the methods (lines 718-719) to make this clearer.

The third aspect is the influence of the longshore wave-driven transport on the sediment fluxes, especially during the post-storm phases. The whole manuscript is written from a cross-shore perspective, and I lack the impact of the wave-driven longshore transport on

morphologic behavior. The authors do not fully neglect the aspect (there is also a longshore transport arrow in Figure 1 (is it the net wave driven longshore sediment transport vector based on the wave climate?), but they do not quantify it in relation to cross-shore fluxes. Especially in the part where the authors describe the volumetric changes between the 4 distinct areas, I would expect more reflection on that.

First, the arrow in Figure 2 (formerly Figure 1) is indeed the net alongshore transport vector which we have now labeled "Net Longshore Transport" for clarity. We do acknowledge the role of alongshore transport in that 1) alongshore transport driven morphological continuums are the framework of the analysis and 2) that "connectivity in the alongshore domain dominates in the post-storm period" (lines 453-454). We thought better of more explicitly trying to quantify the longshore contributions because we don't know the exact input and outputs from each AOI or how much sediment is being lost to the lagoon/sound (e.g., the formation of the flood tidal delta) or what (if any) sediment might be moving further offshore. What we do know is when erosion and accumulation within a given AOI are in balance, and if not, we can use that information to *infer* longshore sediment contributions/losses to each AOI, which we did in Figures 4b and 4c. Further, in the revised manuscript, we have synthesized our findings in a summary figure (Figure 6), in which we attempt to graphically depict our inferences about the magnitudes of longshore contributions. We think this makes what we know about those contributions clearer without overstepping the limitations of the data.

Sometimes, I miss some quantification of the hydrodynamic conditions. For instance, the tidal range in the area (looking to Figure 1, it is probably a wave dominated shoreline), the wave climate of the area (will probably immediately point at the net wave driven longshore transport), the maximum wave height, wave period, direction and duration of the storm, etc. The authors presented the storm characteristics, but I prefer the hydrodynamics of the storm to have an idea of the energy coming towards the shore. As a non-local reviewer, I miss the characterization of the storm and general conditions.

We thank the reviewer for catching this very important oversight on our part. We didn't directly provide any information about the typical hydrodynamic setting of the study area or the storm characteristics. We have added this information to the first paragraph of the methods (lines 520-532) and have also added WIS wave roses for time periods of 40-yrs, the period of observation, and Hurricane Sandy and 4 days on either side of landfall (Figure S1), in addition to plots of significant wave height and period during the study period and the storm (Figure S2) to the Supplemental Information to provide better hydrodynamic context for our observations.

(Specific comments)

What do you mean by 'leading to non-uniform adaptation to forcing changes' on page 2?

We mean that barriers will adapt to changes in their foundational forcings in non-uniform ways. For example, as sea-levels increases, some parts of the barrier may breach/drown whereas other parts of the barrier may transition to new geomorphic states but still remain above sea level. We have changed this to read (lines 40-41): "resulting in alongshore-variable barrier responses to changes in sea level rise, storm climatology, and sediment supply."

What do you explicitly mean by 'surprising ways' on page 2?

We agree that this is vague. We have edited this to "may manifest as near-term stability in geomorphic state at the expense of dynamic behavior that builds long-term resilience." (lines 55-56)

Can you quantify 'extreme water levels' on page 4?

We have added new information to the methods section to address the reviewer's very good point above. Here, we refer to the reader to the methods (line 116).

When did the storm breach occur (you talk about the storm breach Fire Island on page 5)?

During Hurricane Sandy. We have updated the sentence on page 5 to make this clearer (lines 122-125): "Extreme water levels and wave energy associated with Hurricane Sandy breached Fire Island in several places although only Wilderness Inlet, located at the downdrift boundary of Zone 1 and updrift boundary of Zone 2 (Figure 2), was left open to evolve naturally."

You introduced 'inner and outer shoreface' on page 5. Later, reading the methods, I got an idea of the values of the boundaries. Why did you use a boundary based on hydrodynamics (wave climate) and not on morphology (nearshore zone with bars is inner zone, outer shoreface is lower zone). Could you not work with (apply) that general definition?

We could have defined the boundary between the inner and outer shoreface any number of ways. We chose the method we did so that it was repeatable (less subjective…where does the bar end?)) and would allow us to quantify change for storm and non-storm wave conditions. In response to suggestions from another reviewer, we have also edited the methods to clarify how we arrived at that value (lines 667-671).

On page 5, you try to qualitatively say something about the small magnitude of observed net import. A quantification would make this even better.

This was quantified in the previous sentence (e.g., gains exceeded losses by about 6%) and in Figure 3b. No change made.

On page 7, I always ask myself what the normal longshore drift is. You also talk about 'updrift sediment supply' without mentioning the amount, and later on page 8 about 'associated trapping of updrift sediment'. Again, can you quantify the relative importance of longshore transport in relation to cross-shore components?

We don't directly measure longshore drift, but because our analysis is set up with 4 geomorphic zones connected by longshore drift, we invoke that transport process quite a number of times in the discussion to help explain our observations. We can't directly quantify the importance of cross-shore vs alongshore transport, but we get a sense of the dominance of the two from Figure 4b. For the storm-period, when we would expect cross-shore processes to dominate, we see a much better balance between erosion and accretion within a given AOI. In contrast, during the post-storm period, we observe much greater imbalances between erosion/accretion within each AOI, suggesting that cross-shore processes are being interrupted by alongshore transport processes (which human actions are amplifying).

On page 10, you talk about 10 times less high post-storm sediment fluxes. This is probably valid for most of the fluxes. However, the aeolian fluxes will probably be much higher in the post-storm period?

Our analysis is unable to distinguish between the aeolian and overwash contributions to our measurements. For example, in the back-barrier environment, where one might expect aeolian fluxes to dominate, the temporal resolution of our data does not allow us to be certain that overwash fans created during Sandy were not reactivated during the post-storm period, thereby contributing overwash sediment.

On page 10, you talk about dune recovery, does that also include dune formation on wash-over fans?

Yes, see comment above for explanation (pg. 17).

On page 17, I miss the values of the hydrodynamic conditions during the passage of the storm, and general wave (wave rose) and tide (mean tidal range) conditions.

See response above (pg. 18).

On pages 19 and 20, you have a grid cell size of 50 m for the bathymetry. How well can this type of raster point at geomorphic features in the inner shoreface like nearshore bars?

As stated in a response to Reviewer 1 (pg. 7): Shoreface survey resolution is never enough! We would always love to have more data from the shoreface and we see our paper as a(n imperfect) demonstration of the value of these data that will underscore the need for them and hopefully lead to more dense, higher resolution analyses in the future. And we also acknowledge, as the reviewer points out, how critical this information is for understanding storm and post-storm processes, so we sought to present a kind of "reduced-complexity" observational approach that could match the resolution of the data we had. The analysis essentially explores how much sediment is in one box or another, which does not require detailed morphologic information that might be expected from higher resolution data (e.g., sand bar and trough shape). So, the nature of the analysis lends itself well to sparse data, possibly increasing the portability of the methodology since shoreface data are so difficult to acquire. We have added a few sentences to the methods to acknowledge this (lines 577-583).

On page 21, you have quite nice values for volumetric percent errors!

Thank you, we were quite happy with them!

On pages 22 and 23, you have defined the landward limit on sandy features using satellites. Why did you not do it based on your DEMs? There are some possibilities to do that solely based on that, for example (https://doi.org/10.1002/esp.5066).

In comparison to the morphology of the back-barrier environments shown in the reference provided, our study site has considerably more complex morphology (e.g., not flat and often consisting of secondary and tertiary dune ridges. This may have may have made a DEM-based approach used in the reference provided challenging. Furthermore, we wanted to use existing methodologies/published boundaries, where possible. We'd been developing the satellite-derived dataset for a regional project that covered all of New York and New Jersey in parallel with this analysis. Lastly, updated elevation data isn't always available everywhere, so we liked that the satellite methodology could be transferred more easily to other study areas.

On page 25, you talk about 'losses to longshore transport'. How much do you expect?

The revisions to the discussion provide more specifics about the nature of these losses (see lines 390-411) and in terms of how much we would expect, less for the storm period than the post-storm period, which is what is shown in Figure 4b.

On page 26, I didn’t get the value of 1.4 year for the storm-return interval.

We borrow the value from previous work at the study site, hence the citation, but we agree that it is worth spelling out for clarity. In Brenner et al., 2018, they identify 5 storms of record (including Sandy) over 7 years. 7 years/ 5 storms = 1.4 y return interval Some of these are hurricanes and some of these are big extratropical storms, but because some of these bigger storms would generate overwash and offshore directed transport, they need to be accounted for. We have added this text to the methods to provide the appropriate context (lines 796-799).

Some references are not complete, like (6), or not proper styled, like (79).

We thank the reviewer for catching this. Since the paper has undergone significant revisions, we have updated the reference list and have attempted to make sure each of the entries in our reference tracking software is complete.

REVIEWER COMMENTS

Reviewer #1 (Remarks to the Author):

Comment to manuscript NCOMMS-25-73092A

This new version of the manuscript demonstrates a clear commitment by the authors to the topic, as evidenced by the effort they have made to improve the presentation of their results and discussion. I have again enjoyed reading this work, which I find highly relevant and admirably candid in acknowledging its limitations — limitations that can only be addressed if works like this draw attention to the need for more comprehensive monitoring, tackled holistically to include all morphological units relevant to the evolution of the studied system. I have only a few comments that the authors may use to address aspects that, from my perspective, remain somewhat underexplored or underexplained.

1) The authors mention that the interior of the system has gained a very large amount of sediment through the inlet, however, it is not very clear how have they reached to this conclusion given that inlets and deltas are not within the geomorphic units defined by the authors? How was this quantified and which geomorphological unit would gain sediment in this case?

We believe the reviewer is referring to the part of the discussion on lines 353-362, where we discussed the proportion of landward fluxes contributed by the inlet versus washover. The reviewer is correct that we did not measure the flood tidal delta (FTD) component of the inlet. However, we likely captured portions the ebb tidal delta in our shoreface AOIs because it formed on the shoreface. We have now explicitly stated this on lines in the Methods (lines 630-632). As for how we determined what might have ended up in the FTD, we leaned into what we did measure to tell us something about what we didn't measure and also relied on some previously modeled estimates to arrive at our conclusions. We have made some strategic changes to the text on lines 336-349 to better convey this. And in doing so, we came across a typo in our calculations. We estimated that 40% of the sediment lost from the island and shoreface contributed to the FTD, not 50%. We have also updated those portions of the paragraph.

The authors mention that the ebb-delta reorganised after the event, what does this exactly mean? Did the delta loss sediment and then gained again? Did the authors evaluate the changes in the deltas as well?

We could not find a reference to the reorganization of the ebb tidal delta (ETD) in the paper, but we did mention the growth of the ETD on lines, 175, 243, and 464, which we assume is what the reviewer is referring to. ETD growth is commonly known to occur after a barrier island breach forms and as that breach evolves into an inlet, a process that occurred during the period of observation discussed in the

paper. Deposition associated with the formation of the ETD and its growth would be captured in our shoreface observations (see response above and text addition on lines 630-632) if it extended into our AOIs. However, note that the AOIs were delineated after the breach had already formed (see Figure 2b), so not all of the ETD may have been captured. Since inlet equilibration spanned a large portion of our analysis, it is likely that the ETD grew over time, possibly entering our AOIs and we lump that deposition with other changes on the inner/outer shoreface environments in our AOIs. But because of the cadence of our surveys and other changes occurring in the shoreface environments simultaneously, it is not possible for us to resolve specific changes to the ETD alone.

I really appreciate the conceptualisation of the document changes and processes in Figure 6. However, this still does not answer the question of the definition of the different morphological states. The studied area presents originally 4 morphological types (two progradation types, elongation and transgression), these are not what later the authors refer as states? I ask because the authors mention in lines 364 and 365 that morphological states control system response, which is demonstrated with their data. But, later in lines 498 and 499 they mention shifts in alternate states. What are these states, did you document a shift across any of the studied islands? To which extent a change in elevation of the dune from high to medium is a change? A clear statement about what are these alternate states is still missing. I would think this carefully as a change in state may not be driven by a change in the height only, it means more things, see for example the work by Duran and Moore (2015) or Zinnert and Stallins. In this line, in line 506, the authors mention a shift in system state, what is this new state? How do you identify the states? What are the thresholds? What is the name of this new state?

Thank you, we really like Figure 6 too. First, yes, we consider the 4 morphological types along the island to be geomorphic states. We have added text in the results and methods to make this clearer (lines 117 and 534, respectively). Second, we believe the confusion around the question about whether a geomorphic state change occurred is because we weren't clear enough about the relevant spatial scale for our statements. We have edited the last paragraph of the discussion (lines 474-494) to correct this and appreciate the reviewer helping us to get to this point. At the scale of the entire island, or across all barrier geomorphologies, our statement from the previous version holds true: "We do not observe an island-scale transition toward one specific behavior." However, the distinction, and what we believe was the point of confusion, is that we absolutely observe a change in geomorphic state at the "zone-scale" (e.g., immediately adjacent to the inlet). We believe the changes we've made to this paragraph communicate this much more clearly. We hope the reviewer agrees.

Minor comments:

In the introduction, consider using the same nomenclature for what you refer to geomorphological zones avoiding changing to environments, which is used later in the

document, for instance, in the methods section, the authors use environment boundaries. Are these different from the geomorphological units?

We appreciate the reviewer's keen eye here. We have been careful to be consistent in our terminology throughout the paper to avoid confusion, but we can understand how the phrasing in the introduction could be confusing given the context in the rest of the paper. The term "geomorphic zone" is used to refer to variations in geomorphology *along* the island representing the "alongshore domain;" they are described briefly in the Results on lines 117-119 and in detail in the Methods on lines 532-550. In contrast, the term "barrier environments" is used to describe the different environments of which a barrier is comprised (e.g., shoreface, beach, dune, back-barrier) and represents the cross-shore domain. They are defined on lines 629-630, followed by a detailed description of how we defined them (lines 632-721). We have added text on lines 64-65, 80-81, 98, and 101 in the introduction to be clearer about this.

Would it make sense introduce in the introduction section already the morphological types that are represented in your study site?

Capturing the responses in alongshore-variable morphologic zones was definitely part of the scientific motivation for the work, but we feel the *specific* morphological type is less so. In other words, we knew it was important to capture different morphologies, but we weren't picky what those different morphologies were. Lots of barriers are comprised of different geomorphologies, but not all of them have datasets like this one. Therefore, we prefer to keep the descriptions where they are now, which is a brief description in the first paragraph of the results section immediately following the introduction (lines 115-119), and a more detailed description in the methods (lines 532-550).

In the results (line 250), when the authors mention dune volume recovery. Have they detected the formation of new embryo dunes or was this recovery through the formation of an aeolian sand ramp? To which extent this recovery is only "dune" or could be beach? The later related to the difficulty of isolating both units and to which extent the limit of the max runup is the best option.

Because of the scale and style of our analysis, we were not concerned with the specific style of dune recovery, just whether what we defined as the dune environment was gaining the volume that it lost. It would be interesting to explore whether or how the style of dune recovery varied amongst the geomorphic zones, but this is beyond the scope of our analysis. Inspecting the post-storm maps in Supplemental Figures S4-9 does seem to suggest that the style of dune recovery varied between our AOIs (the dune environment is the second polygon from the top in each map); we see more hummocky accretion patterns in AOI6 and a more linear accretion patterns in AOI4, for example, both consistent with the pre-storm dune morphology. We also feel that Supplemental Figures S4-9 provide solid evidence that our methodology is capable of distinguishing between beach and dune environments, with the accretion patterns in the beach environment (third polygon from the top) often being mostly featureless with the exception of occasional beach cusps and the dune environment (second polygon from the top) showing accretion patterns geomorphically aligned with what would be expected of dune formation/growth, even if

the details of the growth isn't apparent at the scale of the map. We will also point out that our success in accurately capturing these environment-appropriate changes was likely due to our methodological design, in which we allowed the elevation boundary contours to shift as shown in Figure S10 and as described in the methods on lines 697-721. If we had allowed the boundaries to be static from the storm period to the post-storm period, it is likely that the signals would have been mixed for inner shoreface, beach, and dune environments. Finally, it is worth noting that our boundary between the dune and beach environment is the upper beach change envelope (Brenner et al., 2018), which is a morphologically-determined boundary not a hydrodynamically-determined value (as runup is).

In the discussion, line 350, would not this result imply that washover accumulation accounted for less than one third?

We were attempting to be conservative but understand that it could cause confusion. The text has been updated (line 333).

The text in lines 365 to 368 may need a bit of more explanation. Do the authors suggest here or further stress the role of inlets contributing to barrier island resilience?
Lines 369 to 370: I am not sure i agree or understand. It seems that there is a bit of mixing of things here. A large immobile dune, if not changing, is because it is maintaining its resilience, correct? Only when a threshold is passed that the dune is not big anymore washover enters into action, and the dune is gone, from the point of view of the barrier it may be negative, but if you think on the time these processes take to evolve not so negative as once the dune is low it will allow and contribute to grow the backbarrier, correct? Why do we have to see high dunes as negative to the resilience of the system? It may only happen at a different moment in time.. what I am trying to stress here is that I would not mix the fact that a breaching can be beneficial with the fact that large dunes are not, resilience may have different dimensions so it should not be considered such a straight forward consequence or simplified vision.

We are presenting one response to both of the comments above since they are related. We think there is confusion here because we expected the reader to know more about citation 26 (Ciarletta et al., 2024) than was probably appropriate. We have made changes to correct this on lines 352-361 and thank the reviewer for pointing out this lack of context.

Line 370: not only human-stabilised inlets but also urbanised areas within the barrier islands, which prevent breaching or dunes to roll over.

Good point. The changes made in response to the comments above address this comment as well (see lines 360-361).

Line 381 and 382: and in the end, the outer shoreface was gaining sediments, correct, maybe also worth stressing it.

Given the complexity of the ideas in this paragraph, we worry that stressing this point here would confuse the message. We also like that we have emphasized this point more completely in the next paragraph, so we've made no change here.

Line 385: I am not sure I get "…that even washover events can drive large volumes of sediment seaward…". Washover event drive sediment inland…do you mean the same events that may induce washover are even more efficient transferring sediment offshore?

Yes, we like this phasing better and have updated the text accordingly (lines 372-375). Thank you.

Line 390: when the authors refer to the lack of efficiency transporting sediment onshore…it seems a bit awkward, are the sediments supposed to move onshore? This might depend on the processes, but also, how do you define the limit between efficiency in the process of moving onshore and not?

We believe "efficiently" is the word we want to use here, particularly in the context of the conclusion sentence of the preceding paragraph (e.g., "shoreface storm deposits and how quickly they are worked back into the terrestrial system") and subsequent sentences where we describe the magnitude of the losses from the outer shoreface storm deposit (e.g., "18% of storm-period sediment remained in the outer shoreface."; "only 33% of sediment eroded from the outer shoreface could have resulted in deposition in other barrier environments."; and "almost half of the sediment deposited in the outer shoreface in our AOIs during the storm period was lost to alongshore transport or beyond our boundaries." In other words, this is a leaky, inefficient process! For clarity, we have made some edits to lines 379-381 that we think improves the framing.

Lines 429-431: this las sentence is very interesting but, i would suggest a bit more explanation to connect the previous with this conclusion given its relevance in connecting temporal scales and processes.

We have edited the sentence to be clearer about the association (lines 423-425).

Lines 439-443: this part seems a bit too elucubrate, the documented differences are not so big, when you also see net import in zone 3, with zone 2 clearly lower, they are like in a gradient. However, my issue here is how can you know that one is functioning as a sink and the other as a remobilisation from deeper parts?

We agree with the reviewer that more work is needed to strengthen these arguments. We have edited out this section of the paper and believe it is stronger for it. Thank you.

In figure 6. It is not clear how in zone 4 the dune is large originally and then becomes very tiny and then it remains the larger in the system? I see that the recovery in zone 4 is large, but does the conceptualisation actually reflects what you have seen?

Yes, the conceptualization matches the observations. The highest storm-period dune volume losses were measured in Zone 4 (Figure 3a; Table S3). The highest post-storm period dune volume gains were measured in Zone 4, more than three times the next closest post-storm dune volume gain (Figure 3b; Table S4). The changes in the conceptual figure are proportional to these measured losses and gains. And as stated on line 197 (and in different words elsewhere in the text), the post-storm response of Zone 4 "resulted from direct nourishment and its position near the littoral end point of the system where it was more likely to benefit from sediments lost from other zones." So, the recovery measured in Zone 4 is a combination of natural recovery and *direct* anthropogenic addition of sediment, though we are not able to determine the timing or proportions of those contributions. Interestingly, no sediment was placed in the eastern ~500m of the AOI and the difference in the post-storm period dune environment accretion signal is visible in Figure S9. On the western side, the accretion displays little alongshore variability and is largely featureless, whereas in the eastern ~500m the accretion is more variably shaped, likely indicative of a more natural addition of sediment to the dune environment. Notably, the direct nourishment also explains the ridiculous proportions of recovery in Zone 4 (500% beach volume recovery!!!). Text has been edited on lines 247 to make the direct nature of the nourishment that occurred there clearer to match other references in the text and the figure caption for Figure 6 has also been edited for clarity.

Reviewer #2 (Remarks to the Author):

Re-reviewed by Brad Murray

The authors have addressed my comments satisfactorily, and the improved paper makes valuable contribution. Thank you for the opportunity to read about this work.

Thank you.

Reviewer #3 (Remarks to the Author):

Comments on the resubmission of the manuscript 'Barrier island geomorphic complexity drives storm and post-storm response', by Miselis et al.

The authors did a great job in the review of the first manuscript. The rebuttal to the first review comments was very extended (21 pages). I went trough the comments on all four

reviewers and I really like the discussion on the topics addressed and the changes they made.

The introduction is now very well structured, and I like the part where they explicitly sketch two critical ways of how and why the drivers of barrier island formation and resilience are going to change. The additional Figure 1 fits also very well and gives a nice summary of a barrier island system.

It is good that the present manuscript has information about the hydrodynamics in the area. That helps to have an idea of the exposure of the barrier island to tides, waves and extreme events.

Figures 3-6 do include a lot of information, but together with the text it contains very valuable results. The results are now easy to follow and Figures 3-5 are very helpful to interpret the changes observed. The extra Figure 6 is very nice for the discussion; it really gives a conceptual explanation and is well embedded in the discussion.

The revised manuscript is worth publishing.

Rev#4 Aart Kroon

Thank you.
